# Species‐Specific Bacterial Associations Emerge From Stochastically Assembled Microbiomes in Northeastern American Fireflies

**DOI:** 10.1111/mec.70473

**Published:** 2026-07-18

**Authors:** Benoît Béchade, Sarah E. Lower, Sierra R. Nichols, Tanner J. Dabbert, Alison Ravenscraft

**Affiliations:** ^1^ Department of Biology University of Texas at Arlington Arlington Texas USA; ^2^ Department of Biology Bucknell University Lewisburg Pennsylvania USA

**Keywords:** community assembly, fireflies, microbiome, *Mollicutes*, species‐specificity, symbiosis

## Abstract

Many insects harbour microbial communities that can profoundly influence the biology of their host. Yet, the relative contribution of random exposure (i.e., stochastic) events and deterministic ecological factors in shaping these communities remains unclear for most taxa. We examined microbiome assembly across 344 firefly (*Coleoptera*: *Lampyridae*) specimens from the Northeastern United States, spanning 12 species and species groups, and generating a high‐resolution dataset through deep 16S rRNA gene amplicon sequencing and quantitative PCR. To formally assess the balance between stochastic and deterministic forces, we applied integrative statistical approaches, including an innovative null‐modelling framework based on the normalized stochasticity ratio (NST) index. We hypothesized that firefly microbiome assembly is dominated by stochastic processes driven by unpredictable microbial exposures. Consistent with this, we observed elevated NST values for most bacteria, coupled with high intraspecific variability in bacterial abundance and composition. However, microbiomes were more similar among closely related fireflies and unusually prevalent mollicute strains showed low NST values, species‐specific associations and retention across geography and host development. While adult bioluminescence and diet could not be directly linked to microbiome abundance or composition, considering seasonal factors and intra‐host anatomy within host species revealed patterns explaining some of the intraspecific microbiome variation. These results show that deterministic processes, likely arising from host‐specific microbial filtering mechanisms, act alongside stochastic forces to shape firefly‐microbe associations. By integrating broad field sampling with quantitative bacterial load estimates and comprehensive microbiome analyses, this study clarifies how evolutionary history, ecology and chance jointly govern microbiome assembly in a diverse insect lineage.

## 1 Introduction

Eukaryotes are constantly exposed to a plethora of microbes in their environment but only a small fraction of those microbes recurrently associate with a host (McFall‐Ngai et al. 2013). Both stochastic and deterministic processes can contribute to the assembly of microbial communities (‘microbiomes’) associated with eukaryotic hosts. Stochastic forces include random host‐microbe encounters across space and time and priority effects (i.e., order of microbial colonizers in hosts) (Jones et al. 2022; Meadow et al. 2013; Sloan et al. 2006; Zhou and Ning 2017), whereas deterministic forces involve microbe‐microbe interactions (e.g., competition, facilitation) and host/habitat filtering—the within‐host conditions or microbial traits that favour the establishment of certain microbes (Coyte et al. 2021; Mazel et al. 2018; Oliphant et al. 2019). However, the degree to which any given microbe is host‐restricted versus environmentally acquired varies widely. Highly specialized symbionts form species‐specific associations (i.e., one microbial strain for one host species), evolve adaptations to a stable host‐associated lifestyle, and are maintained through reliable transmission modes (Kwong and Moran 2015; Michalik and Szklarzewicz 2025). These symbionts influence host physiology and ecology (Bäumler and Fang 2013; Dillon and Dillon 2003), and their communities are largely structured by deterministic processes (Bäumler and Fang 2013; Mazel et al. 2018). In contrast, more generalist and environmentally acquired microbiomes offer flexibility to the host but assemble through more hazardous routes where stochasticity is predominant (Perreau and Moran 2022; Ravenscraft and Coon 2026). Despite progress, the criteria for distinguishing deterministically maintained symbionts versus stochastically assembled microbiome members remain poorly defined.

Fireflies (*Coleoptera*: *Lampyridae*) comprise > 2600 species worldwide (Keller 2025), of which 179 have been described in the US and Canada (Fallon et al. 2021; FireflyAtlas 2025). Many species harbour bacteria from the *Mollicutes* class, which were first detected in culture‐based assays (Hackett et al. 1992; Stevens et al. 1997; Tully et al. 1989, 1994; Williamson et al. 1990), and later recovered in sequencing surveys (Fallon et al. 2018; Green et al. 2021). These findings hint that some firefly‐associated mollicutes may represent stable, potentially host‐filtered symbionts. Yet, fireflies also carry common insect endosymbionts (i.e., symbionts residing inside the body or cells of another organism), such as *Wolbachia* (Jeong et al. 2009; Jeyaprakash and Hoy 2000), and diverse environmental bacteria (Green et al. 2021; Zhao et al. 2023), suggesting stochastic acquisition plays a substantial role as well. Importantly, it remains unclear how specialized these mollicutes are to fireflies. Are they lineage‐specific symbionts or flexible associates that track ecology and environment? This largely unexplored, diverse insect group provides a powerful opportunity to evaluate how microbial symbionts vary in specificity and to test when deterministic versus stochastic assembly predominates.

**FIGURE 1:**
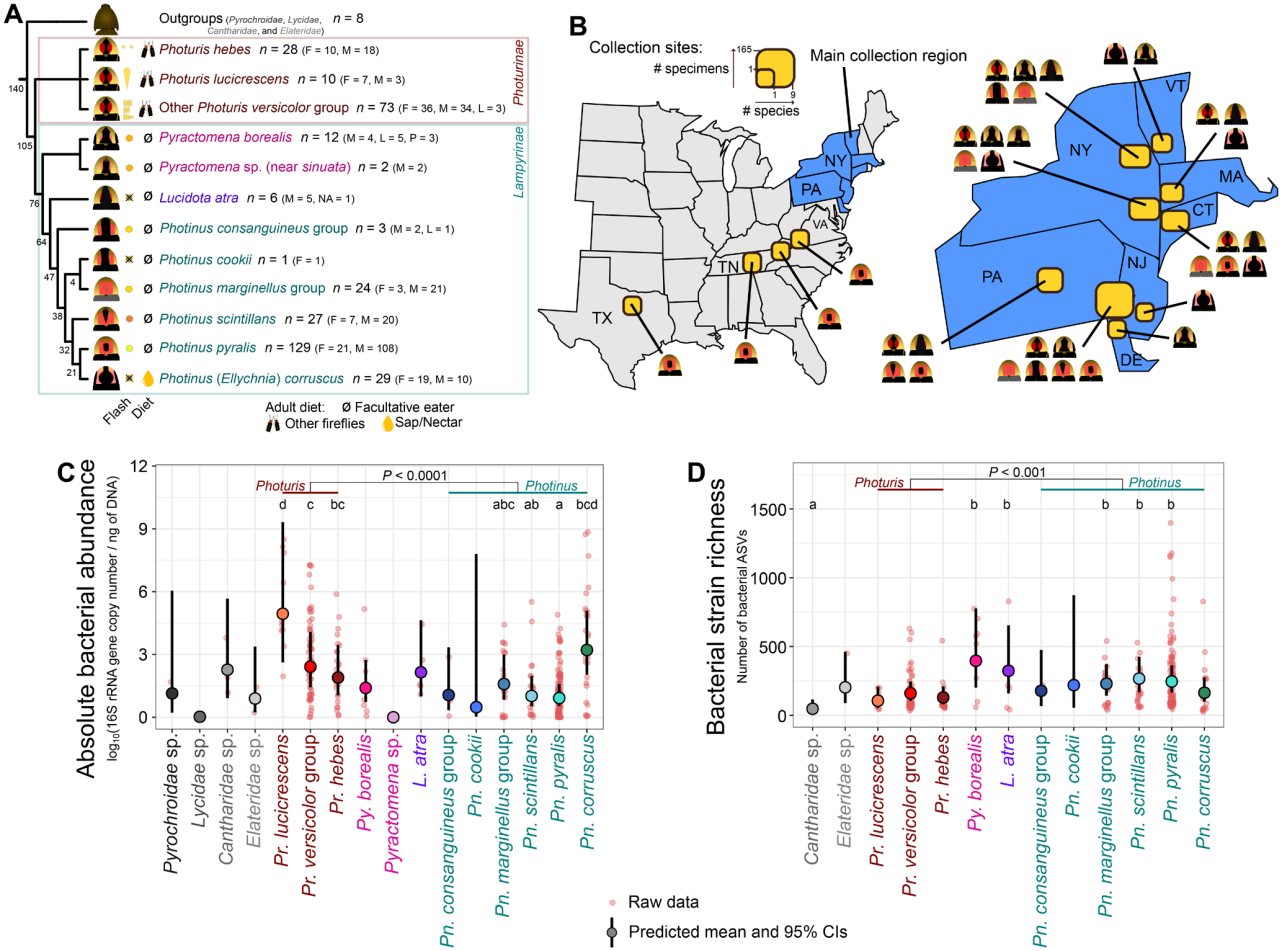
Sampling overview and firefly microbiome abundance and diversity across species. (A) Species sampled and analysed in the present study. The phylogenetic tree is adapted from Catalan et al. (2022) and Martin et al. (2019). Numbers at each node indicate estimated divergence time from Catalán et al. (2025), Höhna et al. (2025), and Powell et al. (2022) in millions of years. The cartoons next to the tree branches are simplified representations of the typical markings visible on the pronotum of different firefly species. Symbols next to these pronotal depictions correspond to the most common male flash patterns for the species or species group, with the frequency of the flashes for *Photuris* species, and whether a firefly is bioluminescent (coloured flash) or not (crossed out). The next symbols correspond to adult diets, with ‘ø’ for species that are not known to eat as adults, other fireflies for predatory diet, and a yellow droplet for sap and nectar drinkers. The number of specimens collected and processed is shown next to species' names, with ‘F’ for females, ‘L’ for larvae, ‘M’ for males and ‘P’ for pupae. (B) Map of the central and eastern United States and Northeastern states showing the location of sampling sites. While a few *Pn. pyralis* specimens were collected in Tennessee, Texas and Virginia, most fireflies were collected in Northeastern states (blue colour). The yellow rectangles give the number (‘#’) of species (width) and specimens (height) collected in the region and the cartoons of firefly pronotum indicate which species were collected, corresponding to the cartoons from panel A. Specimen information can be found in Table S1. (C) Normalized absolute bacterial abundance across all firefly and related insect samples. Values are expressed as the log_10_ of the number of 16S rRNA gene copies in a sample, measured through qPCR, minus the number of 16S rRNA gene copy in the DNA extraction blank sample from the same batch, and then divided by the total DNA concentration in the sample, which was measured via Qubit. Least‐square means (emmeans) post hoc tests on generalized linear mixed models (GLMMs), were used for statistical testing of differences between genera and species, and controlled for sex, day of year, month of capture, time of capture, longitude at capture, local sampling point, and whether the specimen was dissected or not. Lowercase English letters indicate the significance of the difference across firefly species. Letters for species that are not significantly different from any other species (i.e., *p* > 0.05: ‘abcd’) were removed. Balls and error bars correspond to predicted average and 95% confidence intervals obtained from the GLMM. Dots represent raw data points. (D) Bacterial richness, expressed as the number of bacterial ASVs, across firefly species. The *Photinus* microbiome contains, on average, a higher bacterial strain richness than that of *Photuris* (emmeans post hoc tests on GLMMs, controlling for genus, sex, local sampling point, day of year, year of capture and sequencing type). See description of panel D for details on graph components. All alpha diversity metrics can be found in Table S7 and Figure S7.

Fireflies exhibit diverse life histories and ecologies (Faust 2017; Lloyd 2018) that can shape, or be shaped by, associated microbial communities in deterministic ways. First, as holometabolous insects that include larval, pupal and adult stages, their ecology and physiology shift drastically across development, potentially producing stage‐specific microbiomes. Adult lifespan also varies widely, with most species living only a few weeks, while winter fireflies (*Photinus corruscus*) can live up to 10 months as adults (Deyrup et al. 2017; Rooney and Lewis 2000). Age affects susceptibility to inoculated bacterial pathogens (Lower et al. 2023), but its influence on the wild microbiome remains largely unexplored. Second, dietary ecology differs among adults (Figure 1A), which may impose drastically divergent physiochemical conditions upon microbial gut colonizers. While larvae generally prey on soft‐bodied invertebrates (Buschman 1984a, 1984b, 1988; Fu and Benno Meyer‐Rochow 2013; Hess 1920; Vaz et al. 2020, 2021; Yang et al. 2024), adult diets vary from predation on other fireflies in *Photuris versicolor* species group (Lewis et al. 2012; Lloyd 1965), to sap and nectar feeding in *Pn. corruscus* (Rooney and Lewis 2000), and many species may not feed at all (Lloyd 2005; Wing 1989; but see Faust 2017; Faust and Faust 2014; Lloyd 1998; Othman et al. 2018). Third, although all larvae use bioluminescence for anti‐predator defence (Oba et al. 2020; Powell et al. 2022), some lineages have lost adult light production (Figure 1A) (Martin et al. 2017; Stanger‐Hall et al. 2007; Stanger‐Hall and Lloyd 2015; Zaragoza‐Caballero et al. 2023). Bacterial symbionts are often implicated in bioluminescence of invertebrates (Cassells et al. 2024; Jones and Nishiguchi 2004), but nothing is known about microbial involvement in firefly bioluminescence, nor about how microbiomes differ between bioluminescent versus non‐bioluminescent adult hosts. These life history and ecological contrasts create strong opportunities for microbial specialization, but whether firefly‐associated microbes are stable, host‐filtered symbionts or flexible, opportunistic colonizers remains unresolved.

Here, using field‐collected fireflies from 12 species and species groups across the Northeastern United States (Figure 1A,B; Figure S1; Table S1), we leverage natural variation in firefly ecology and phylogeny to ask how evolutionary history and host biology shape microbial assembly. Closely related firefly taxa differ markedly in diet, bioluminescence, habitat, seasonality and life history, providing a unique system in which to disentangle the effects of host lineage, ecology, environment, and their interactions within a single clade. Using quantitative (q)PCR, 16S rRNA gene amplicon sequencing and integrative statistical analyses (Figure S2), we test whether mollicutes represent host‐restricted symbionts structured by deterministic forces, and whether the rest of the microbiomes is largely composed of non‐specific, transient bacteria shaped by stochastic processes. We predict that although total bacterial load and overall community composition vary among individuals and species, most firefly species harbour their own distinct mollicute lineage. By situating microbiome variation within a phylogenetically diverse, yet tractable, insect group, this study addresses the degree of specificity of firefly‐associated mollicutes, establishing fireflies as a powerful system for dissecting how ecological and evolutionary processes maintain host‐microbe associations.

## 2 Materials and Methods

### 2.1 Specimen Collection and Identification

A total of 344 firefly specimens (*Coleoptera*: *Lampyridae*) were collected opportunistically in the continental United States in 2021, 2022 and 2023 (Figure 1A,B; Table S1). We captured specimens by hand or with a net, preserved them in > 95% ethanol upon collection, and froze them within the next 72 h. Firefly specimens were initially identified and sexed via examination of flash patterns, external morphology and genitalia (Faust 2017; Fender 1970; Green 1956, 1957; McDermott 1967). To verify species identifications, up to 27 specimens from each species and species group were DNA barcoded, for a total of 59 specimens. We extracted DNA as described below and amplified fragments of the universal metazoan cytochrome C oxidase subunit 1 (COI) gene marker using four different primer pairs (Folmer et al. 1994; Geller et al. 2013; Rennstam Rubbmark et al. 2018; Simon et al. 1994; Stanger‐Hall et al. 2007). A full description of the barcoding methods is available in Data S1 with results shown in Figure S1. DNA barcoding sequences are available as NCBI GenBank files, with accession IDs PV844642‐PV844700.

The firefly specimens belonged to two subfamilies, four genera and 12 species or species groups (Figure 1A). Among them, 332 were adults, including 104 females, 227 males and one *Lucidota atra* specimen that was not sexed, and 12 were juveniles, with nine larvae and three pupae. Ten adult beetles from other families were also collected as outgroup specimens. They included three soldier beetles (*Cantharidae*), two tiger beetles (*Cicindelidae*) that were already dead upon collection, three click beetles (*Elateridae*), one net‐winged beetle (*Lycidae*) and one fire‐coloured beetle (*Pyrochroidae*).

### 2.2 Dissection and DNA Extraction

To study the bacterial communities of fireflies, we extracted DNA from the whole body of 321 specimens as described in Data S1. To infer microbiome variation across tissues, we randomly selected among our preserved specimens 5–6 adult females and males from each of three well‐represented firefly taxa (*Photinus corruscus*, *Pn. pyralis* and the *Pr. versicolor* group; Table S2). To isolate tissues, firefly specimens were dissected in sterile water using bleach‐sterilized fine forceps following surface sterilization. Separated tissues were placed in pre‐chilled 1.5 mL tubes, either filled with 95%–100% ethanol or empty, and stored at −80°C. During the dissections, we separated the entire gut, the reproductive organs and the lantern (for bioluminescent species) from the rest of the body, which was used as the fourth main tissue (i.e., ‘carcass’). To investigate the possibility of endosymbionts (i.e., symbionts residing inside the body or cells of another organism) and/or reproductive manipulators, for a few *Pn. corruscus* and *Pn. pyralis* specimens, we additionally separated some of these main tissues to obtain enlarged fat tissue (‘globules’), genitalia, ovaries, hindgut, foregut and midgut, spermatheca, spermatophore‐digesting glands and testes. Dissection blank samples consisted of a firefly leg left in a water droplet on the side of the dissection area. To control for contaminants during dissection, we used a new dissection blank for every one to three dissected specimens. In total, we dissected 33 adult fireflies, resulting in 132 firefly tissue samples and 15 dissection blank samples (Figure S2). We extracted DNA from dissected tissue samples as described in Data S1.

We obtained a total of 497 DNA samples, including 321 whole‐body samples, 132 dissected tissue samples, 15 dissection blanks and 29 DNA extraction blank samples.

### 2.3 Bacterial Titre and Total DNA Quantifications

Total bacterial titre per sample was obtained through qPCR (Table S3). CT values were generated with a 7300 Real‐Time PCR System (Applied Biosystems, Foster City, CA, USA). We used the V4 region of the 16S rRNA gene as the target, amplified using the bacterial universal 515F (5′‐GTGCCAGCMGCCGCGGTAA‐3′) and 806R (5′‐GGACTACHVGGGTWTCTAAT‐3′) primers (Apprill et al. 2015; Caporaso et al. 2011; Parada et al. 2016). To obtain absolute bacterial titers measured in 16S rRNA gene copy numbers, we used, in each qPCR plate, a standard curve built on a ten‐fold dilution series of a DNA sample with known 16S rRNA gene copy number. Bacterial titers were then corrected using DNA extraction blank and dissection blank samples for tissue samples. Each sample was run in two or three separate technical replicates, consisting of runs on separate qPCR plates. We calculated the average of blank‐corrected titers across plate replicates and used these values in downstream statistical analyses. A detailed description of our qPCR procedure can be accessed in Data S1. To normalize bacterial titers and for our Illumina amplicon sequencing decontamination process (see subsection 5.1), we quantified the concentration of extracted DNA per sample using a Qubit 4 fluorometer (Invitrogen, Waltham, MA, USA) and Qubit dsDNA HS and BR kits (Thermo Fisher Scientific, Waltham, MA, USA), with two to three technical replicates per DNA sample.

### 2.4 Illumina Library Preparation and Sequencing

A first batch of 20 samples was sequenced at the University of Texas at Arlington Genomic Core Facility in 2021. The library was prepared following Illumina's two‐step amplification strategy using primers 341F (5′‐CCTACGGGNGGCWGCAG‐3′) and 785R (5′‐GACTACHVGGGTATCTAATCC‐3′), which amplify the V3‐V4 hypervariable region of the 16S rRNA gene (Klindworth et al. 2012), as described in Ravenscraft et al. (2024). Each sample was bidirectionally sequenced on a 600 cycle paired‐end run on an Illumina MiSeq platform (Illumina, San Diego, CA, USA). The second batch, containing 477 samples plus one sequencing blank, was sequenced at the Environmental Sample Preparation and Sequencing Facility at Argonne National Laboratory in 2024 (Table S4). The V4 region of the 16S rRNA gene was PCR‐amplified with 515F and 806R primers (Caporaso et al. 2012). Amplicons were sequenced on a 300 cycle paired end run in a NextSeq 2000 instrument (Illumina) using customized sequencing primers and procedures. For both runs, demultiplexed data were then processed in R (R Core Team 2024) for downstream analysis.

### 2.5 Bioinformatic Pre‐Treatment

#### 2.5.1 Pre‐Processing of Sequencing Data

For pre‐treatment and analyses, default parameters were used unless indicated. For the MiSeq sequencing run, priming sites and poor‐quality bases were removed from the 5′ and 3′ ends of the sequences using the cutadapt v3.2 program (Martin 2011). This step was not necessary for the NextSeq sequences, which did not include priming sites. Then, for each sequencing run, we used the *DADA2* v1.32.0 R package (Callahan et al. 2016) to remove low quality reads, merge paired ends and infer the bacterial strains present, generating amplicon sequence variants (ASVs). We merged data from the two sequencing runs, performed *de novo* chimaera removal, and ran taxonomic classification through the RDP classifier and with a modified SILVA SSU v138.1 training dataset (Quast et al. 2013; Wang et al. 2007). Further details on the pre‐processing procedure are available in Data S1. Filtered amplicon sequences were exported in fasta format using the *seqinr* v4.2.36 R package (Charif and Lobry 2007) and are available in our data repository (https://doi.org/10.6084/m9.figshare.32599836). Since ASVs from the 20 samples on the MiSeq run were a longer, partially overlapping region of the 16S rRNA and therefore could not be merged with the NextSeq ASVs, we performed all analyses at higher taxonomic levels (e.g., bacterial genera) and grouped key bacterial taxa phylogenetically (see section 5.2 below).

The ASV count table, the taxonomic classification table and the metadata information (including bacterial titre obtained from qPCR and DNA concentration per sample) were converted into a phyloseq object that contained 99,979 ASVs, using the *phyloseq* v1.48.0 and *speedyseq* v0.5.3.9021 R packages (McLaren 2020; McMurdie and Holmes 2013). Following length filtering and removal of chloroplastic, mitochondrial and blank‐only ASVs (Data S1), we used the *decontam* v1.24.0 R package (Davis et al. 2018), with the stringent ‘either’ frequency or prevalence method to discard contaminant ASVs. With this method, 1555 ASVs were flagged as contaminants and removed (discarding 2% of the remaining sequences; list available in our data repository: https://doi.org/10.6084/m9.figshare.32599836), resulting in 53,165 ASVs being retained in our dataset. While we removed a high number of ASVs classified as common contaminants with this method (e.g., *Klebsiella*, *Pseudomonas*, *Salmonella*, *Staphylococcus*), we note that it discarded an abundant ASV from the *Acinetobacter* genus; similar studies have also sequenced *Acinetobacter* from fireflies and it is possible this is a common environmental colonist of firefly guts (Green et al. 2021). In addition, a list of bacteria that passed our decontamination pipeline but that could conceivably be contaminants, because they were only relatively abundant in specimens that had a very low bacterial titre, is shown in Table S5 with more details in Data S1.

Finally, to control for among‐sample differences in sequencing depth, our data were rarefied to 30,000 sequences per sample using *phyloseq* (Schloss 2023, 2024; Weiss et al. 2017). Visual inspection of rarefaction curves indicated that observed diversity had plateaued in the samples by this depth. Because rarefying would remove important samples in our dissected tissue dataset, we instead used compositional (i.e., proportional) normalization for tissue comparative analyses, computed with the *microViz* v0.12.4 R package (Barnett et al. 2021). Similarly, rarefied data were not used for our multivariate ordination analyses based on Bray‐Curtis distances, since compositional normalization has been shown to be a more robust normalization method for clustering on Bray‐Curtis distance matrices (McMurdie and Holmes 2014) and because rarefying caused some species to be removed from our dataset (data from our two *Pyractomena* sp. near *sinuata* whole body specimens did not pass the rarefaction threshold).

#### 2.5.2 Taxonomic Annotation Refinement Through Phylogenetic Analyses

We next performed phylogenetic analyses to refine the taxonomic identification of abundant and prevalent bacterial clades in fireflies (*Mollicutes*; Figure S3), common arthropod endosymbionts (*Rickettsiaceae*, *Rickettsiella* and *Wolbachia*; Figure S4), and insect pathogens (*Serratia*; Figure S4). For the *Mollicutes*, we used the terminologies from (Gupta et al. 2019), although other authors classify most of the firefly‐associated non‐*Spiroplasma* mollicutes as the *Entomoplasma* genus (Balish et al. 2019; Gasparich and Kuo 2019; Lo et al. 2018; Yan et al. 2024; but see Arahal et al. 2022; Gupta et al. 2018; Gupta and Oren 2020). Because our data came from two sequencing batches that used two different amplicon lengths, hence classifying identical variants as two different ASVs, we used phylogenetic analyses to group together ASVs that were likely from the same microorganism but were classified as different variants due to different amplicon sizes. We use the term ‘bacterial phylotype’ hereafter in this section, approximately equivalent to bacterial species, to refer to this classification, which we performed only for the five aforementioned bacterial clades (*Mollicutes*, *Rickettsiaceae*, *Rickettsiella*, *Serratia* and *Wolbachia*). Details on phylogenetic methods can be found in Data S1.

We manually edited the *phyloseq* taxonomy table to account for this taxonomic refinement (available as a Data Repository: https://doi.org/10.6084/m9.figshare.32599836). Except for analyses on alpha diversity metrics and specific mentions of analyses ran at the bacterial order or genus levels, all downstream analyses were run on microbiome data aggregated at the genus level, except for the aforementioned groups we refined (*Mollicutes*, *Rickettsiaceae*, *Rickettsiella*, *Serratia* and *Wolbachia*), which were aggregated at the ‘phylotype’ level (Figure S2). This combination of genus and phylotype level classification is hereafter referred to as ‘taxon’ level. Data was aggregated at the bacterial order, genus and taxon levels using the *microViz* R package.

### 2.6 Bioinformatic Analyses

#### 2.6.1 Stochasticity Analyses

To infer the relative importance of stochastic versus deterministic processes contributing to microbiome assembly, we calculated the normalized stochasticity ratio (i.e., NST index) for each firefly species that had more than two specimens in our dataset through implementation of null‐models using the *NST* R package v3.1.9 (Ning et al. 2019). We calculated the NST index based on Bray–Curtis dissimilarity metrics using the null model algorithm ‘PF’ (fixed data richness and proportional taxa occurrence frequency), taxon relative abundance and 999 randomizations. An index value between 0.5 and 1 suggests that stochastic processes shape community assembly, whereas a value between 0 and 0.5 indicates higher influence of deterministic processes. Next, to infer the contribution of specific bacterial groups, we first tested how the removal of either all the mollicutes, all putative endosymbionts (*Ca*. Hepatincola, *Rickettsia*, *Rickettsiella* and *Wolbachia*), or both, from the microbiome data of each firefly species affects the NST index value. We then tested whether removing each of 16 highly represented bacterial genera in our amplicon sequencing data set altered the NST index values. We generated 95% confidence intervals via bootstrapping and tested NST variation between the unaltered microbiome of firefly species versus that following removal of the aforementioned bacterial groups through pairwise, two‐tailed Wilcoxon signed‐rank statistical tests (Table S6).

#### 2.6.2 Alpha and Beta Diversity

To assess patterns of diversity, richness, evenness, dominance and rarity in bacterial communities from fireflies, indices of alpha diversity were calculated on rarefied data with non‐aggregated (i.e., ASV level) taxonomic tables, using the *microbiome* v1.26.0 R package (Lahti and Shetty 2017) (Table S7). To compare bacterial composition across firefly species, genera, subfamilies and diverse factors, we used beta diversity metrics, first running ordinations on Bray‐Curtis distance matrices computed from non‐rarefied, proportional abundance (i.e., compositional normalization) data using nonmetric multidimensional scaling (NMDS) analyses performed with *phyloseq*, to infer similarities among fireflies from different taxa, diets, adult use of bioluminescence, regions and tissues. To test how different factors were correlated with bacterial composition across fireflies, we then ran permutational multivariate analyses of variance (PERMANOVAs) on rarefied, taxon‐aggregated data, with the *vegan* v2.6.4 R package (Oksanen et al. 2016), and pairwise PERMANOVAs as post hoc tests using the *pairwiseAdonis* v0.4.1 R package (Martinez 2020).

#### 2.6.3 Host Relatedness, Geographic Distance and Microbiome Dissimilarity

The impact of firefly host phylogeny on bacterial community dissimilarity was assessed using Mantel tests. We first generated a host phylogeny using five previously sequenced firefly genes (Catalan et al. 2022; Martin et al. 2017; Sander and Hall 2015; Stanger‐Hall et al. 2007; Stanger‐Hall and Lloyd 2015) (Figure S1; Data S1). We then used the *ape* v5.8 R package (Paradis et al. 2004) to generate a phylogenetic distance matrix from our tree with the cophenetic.phylo() function. We next produced a matrix of geographic distance among collected firefly specimens calculated from latitude and longitude decimal variables at collection using the *sf* v1.0.17 R package (Pebesma 2018). As proxies for microbiome dissimilarity, we generated two pairwise distance matrices using the Bray‐Curtis distance from rarefied relative bacterial abundance between firefly specimens and the Bray‐Curtis binary distance from bacterial presence/absence (equivalent of Sørensen‐Dice distance) between firefly specimens. Bacterial presence was defined as any bacterial taxon with at least 1% relative abundance in a specimen and absence as less than 1% relative abundance in that specimen. Because the number of pairwise comparisons was not computationally tractable, we applied a filter to our dataset, keeping only bacterial taxa that had more than 0.5% relative abundance in at least 5% of all firefly specimens. These microbiome dissimilarity matrices were compared to a matrix of phylogenetic distance and to geographic distances between firefly specimens through partial Mantel tests using the *vegan* R package, allowing us to control for host phylogeny and geography, respectively.

#### 2.6.4 Contribution of Firefly Ecological Traits to Bacterial Abundance and Diversity

We tested the influence of adult bioluminescence, diet and body size of firefly species on bacterial abundance and diversity, as well as correlations between bacterial abundance and diversity metrics via generalized least square analyses with the *nlme* v3.1.164 R package (Pinheiro et al. 2024). To account for the effects of phylogenetic nonindependence in comparisons of firefly species‐level covariates, we used phylogenetic generalized least square (PGLS) analyses, fitting a Brownian motion model of evolution. The firefly phylogeny was obtained as described in section 6.3 and Data S1 (Figure S1). Bacterial titre and diversity indices were averaged per firefly species. We also averaged body size (i.e., total adult male body length) per firefly species or species groups using ranges from previous studies (Faust 2017; Fender 1970; Green 1956, 1957; Lloyd 1969).

#### 2.6.5 Bacterial Composition and Differential Abundance Analysis

To test whether single bacteria vary in their abundance across fireflies and factors, we used the *MaAsLin2* v1.15.1 R package (Mallick et al. 2021), fitting our rarefied (or non‐rarefied for tissue analyses) data to negative binomial distributions (which fit well to our datasets) and controlling for random effects (see section 6.7. Statistical tests) in multiple generalized linear mixed models. To account for variables with unbalanced discrete levels (e.g., species with high versus low number of specimens sampled), when more than two discrete levels were present in our fixed effect(s), we ran the same *MaAsLin2* models multiple times, changing the reference level each time (Tables S8–S11). We filtered out bacterial taxa detected in less than 1% of all firefly samples being tested, unless the test was run on groups with very low sample size (prevalence threshold lowered to 0.1% instead).

#### 2.6.6 Bacterial Co‐Occurrence Analysis

To investigate positive and negative co‐occurrence patterns among bacteria in adult fireflies, we ran co‐occurrence analyses, within firefly species that had more than eight adult individuals in our dataset (i.e., excluding *Lu. atra*, *Pn. consanguineus* group, *Pn. cookii* and *Py. borealis*) and grouping all *Photuris* together. We used both the *cooccur* v1.3 and *CooccurrenceAffinity* v1.0 R packages (Griffith et al. 2016; Mainali and Slud 2025). A filter was applied before analyses to obtain a manageable number of bacterial taxa to be tested per firefly species. Bacterial taxa were considered present when they had at least 1% relative abundance (absent otherwise) in a specimen and were kept in these analyses when they had more than 10,000 reads—or 20,000 reads for the *Photuris—*in one or more samples for a given firefly species or species group.

#### 2.6.7 Statistical Tests

In addition to the tests described in the previous sections, differences in alpha diversity and qPCR titers across hosts and diverse environmental factors were assessed using linear mixed models (LMM) with the *lme4* v1.1.35.5 R package (Bates et al. 2015), which allowed us to control for random effects that may otherwise bias our interpretation of statistical tests. Some key factors were always kept as random effects in our models because they had a strong influence on the microbiome of fireflies in our datasets. These random factors included the year of collection, the geographic location of sampling (either address, longitude, latitude or region), and collection day or month of year (a rough proxy for firefly age in many species). When they were not among the fixed effects in our models, host sex (which included developmental stages) and species were used as random effects or covariates. The importance of other random effects in our models had been tested through bidirectional model selection with likelihood ratio tests and comparing model's AICs.

We tested the distribution of the residuals, percentage of the dispersion of the data, the presence of outliers, and zero‐inflation using the *performance* v0.12.3 and *DHARMa* v0.4.6 R packages (Hartig 2018; Lüdecke et al. 2021). Our models were often flagged as poor fits due to zero‐inflation, despite normalization and log_10_ transformation. When this was the case, we fitted our data to a negative binomial distribution in generalized LMMs (GLMMs) using *lme4*. We next performed pairwise post hoc tests using the *emmeans* v1.10.4 R package (Lenth 2022) to compute the estimated marginal means (Searle et al. 1980). In doing so, we applied an asymptotic degrees‐of‐freedom method, thus invoking *z*‐statistics, which are computationally faster and recommended with larger sample sizes. A description of all LMMs, GLMMs, PERMANOVAs, pairwise PERMANOVAs, Mantel tests, OLS and PGLS models is available in Table S12.

Additional files, including sequencing OTU and taxonomy tables, list of contaminant variants removed, multiple sequence alignments, photographs of insect specimens, multi‐locus backbone phylogenetic trees, R script and input files and additional Supporting Information, are available in our data repository at https://doi.org/10.6084/m9.figshare.32599836.

## 3 Results

### 3.1 The Microbiome of Fireflies Is Mostly Structured by Stochastic Processes, Except When Bacteria From the *Mollicutes* Are Included

**FIGURE 2:**
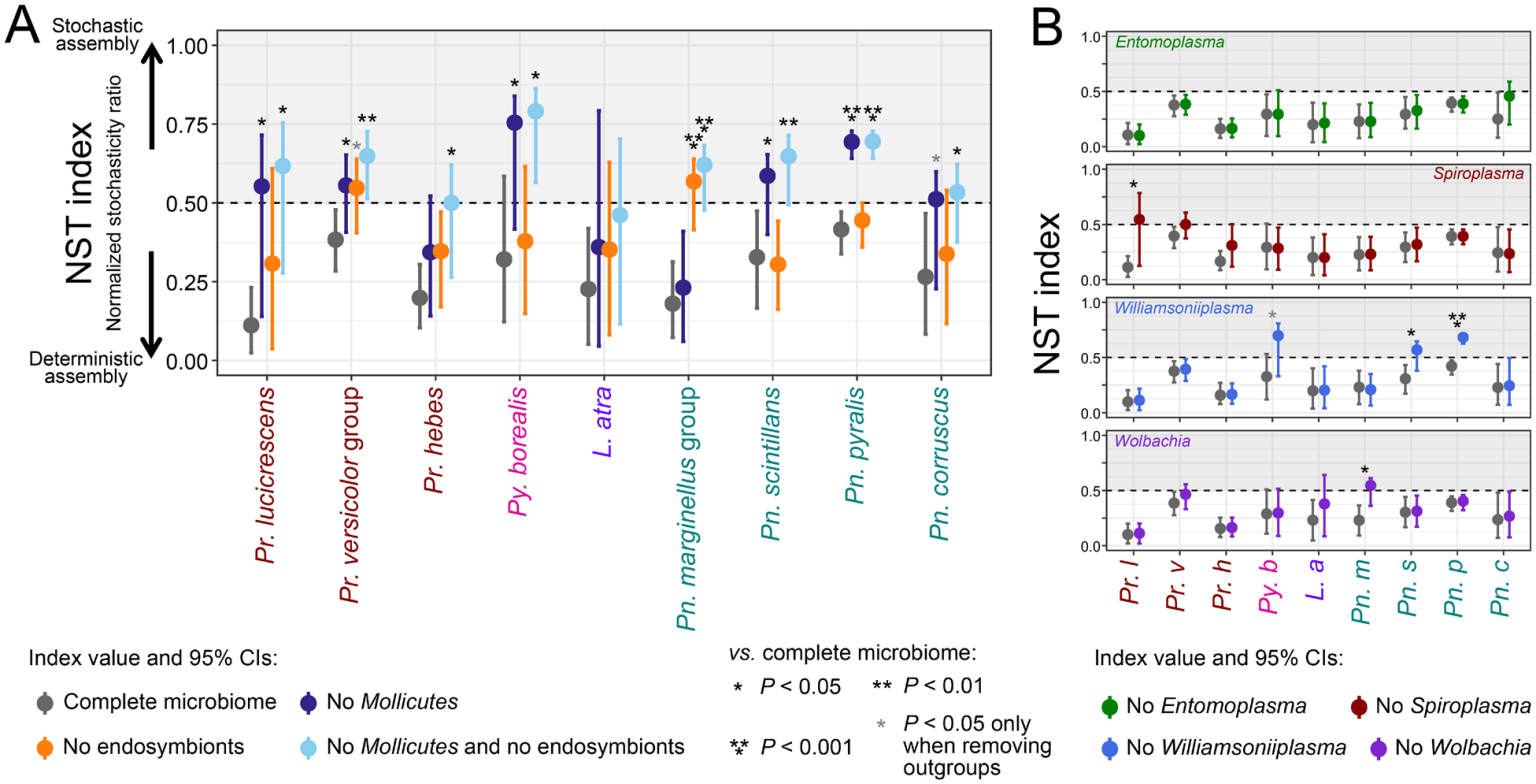
Normalized stochasticity ratio (NST) across firefly species. (A) NST index values higher than 0.5 indicate a higher importance of stochastic forces governing bacterial community assembly, whereas values lower than 0.5 point to deterministic forces as the main drivers of community assembly. For each firefly species or species group the balls correspond to the index value and the error bars are 95% confidence intervals obtained from bootstrapping. Dark grey colour is for the complete, untouched microbiome, dark blue corresponds to microbiomes in which all the mollicutes were removed from the dataset, orange is for microbiomes in which putative endosymbionts (*Candidatus* Hepatincola, *Rickettsia*, *Rickettsiella* and *Wolbachia*) were removed, and light blue corresponds to microbiome datasets in which both mollicutes and endosymbionts were removed. The asterisks depict, for each species, significantly different NST values between the complete microbiome and either of the modified microbiome datasets. Two‐tailed Wilcoxon signed‐rank statistical tests were used to test the significance of these differences. Asterisks in light grey are for tests run on datasets where outlier data points (i.e., values higher or lower than 1.5‐fold of the interquartile range) were removed. (B) Comparison of NST index in the complete microbiome in dark grey versus the same microbiome in which a key bacterial genus was removed. Results for additional bacterial genera are available in Figure S5 and index values and statistical test results can be found in Table S6.

To assess the relative importance of deterministic versus stochastic factors in structuring firefly bacterial communities, we calculated and compared normalized stochastic ratios (NST) (Ning et al. 2019) on our amplicon sequencing dataset, focusing on firefly species that had more than two specimens in our dataset. The microbiome of all nine firefly species tested had an NST index way below 0.5 (Figure 2A; Table S6), indicating that their microbiome is mainly structured by deterministic processes. To infer which bacterial group may be responsible for deterministic community assembly, we first removed all the *Mollicutes* from our dataset and found that the NST significantly increased for five out of nine firefly species (Wilcoxon signed‐rank test for these five species: *W* < 2.3 × 10^4^, *p* < 0.05), reaching index values above 0.5. When removing individual bacterial genera, the mollicute genus *Williamsoniiplasma* appeared to be driving this result in *Photinus scintillans* , *Pn. pyralis* and *Pyractomena borealis* , whereas *Spiroplasma* was involved in *Photuris lucicrescens* deterministic microbiome assembly (Figure 2B; Table S6). Interestingly, for members of the *Pn. marginellus* group, removing mollicutes did not change the NST values, but removing bacteria known as endosymbionts (i.e., symbionts residing inside the body or cells of another organism) of invertebrates, such as *Wolbachia*, instead significantly increased the NST index (Figure 2A,B; Table S6). Other bacterial genera that are prevalent in fireflies did not seem to be involved in deterministic assembly of their host microbiome (Figure S5; Table S6). This suggests that for several firefly species, while stochastic factors are involved in structuring most of the bacterial community, deterministic processes shape the relative abundance of either mollicutes or *Wolbachia*, influencing the whole microbiome.

### 3.2 The Microbiome of Fireflies Is Phylogenetically Structured

#### 3.2.1 Firefly Genera Differ in Their Bacterial Density and Diversity

Using qPCR, we found that *Photuris* fireflies had significantly higher bacterial densities than *Photinus* fireflies (Table S12), despite high intraspecific variability (Figure 1C; Figure S6). The *Pyractomena* and *Lucidota* genera were much less represented in our dataset, and members of these groups appear to have relatively low, but variable, bacterial levels. Within firefly genera, *Pn. corruscus* stood out from other *Photinus* species, with its long‐lived, day‐active adults carrying bacterial loads three orders of magnitude higher, approaching those observed in *Pr. lucicrescens*. The difference between *Pn. corruscus* and other *Photinus* species was, however, only significant when comparing bacterial titers of *Pn. corruscus* to *Pn. pyralis* (emmeans: *z*‐ratio = 3.67, *p* < 0.05). This indicates that, with the exception of *Pn. corruscus*, members of the *Photinus* genus tend to be infected with low to extremely low bacterial densities in the wild, while *Photuris* fireflies have moderately high to very high bacterial titers. Importantly, the median bacterial density was, in each firefly species, lower than the average (Figure S6), indicating that a few firefly specimens harbour a very high bacterial density, whereas most specimens keep low bacterial densities.

In terms of bacterial richness, despite their relatively low bacterial densities, fireflies from the *Photinus* genus harboured significantly more bacterial ASVs than *Photuris* fireflies (Figure 1D; Figure S7; Tables S4 and S12), suggesting that fireflies with higher bacterial densities tend to harbour fewer bacterial species. This is partially supported by the weak negative correlation between absolute bacterial abundance and bacterial richness and diversity (OLS and PGLS: *t* < −2.15, *p* < 0.06; Figure S10). This result highlights a possibly important contrast across firefly species, suggesting that these insects either harbour a low density of diverse bacteria, as for *Lucidota atra* and most *Photinus* species or a bacterial community dominated by a few strains with high absolute abundance, as in *Pn. corruscus*, *Photuris* species and *Py. borealis*.

#### 3.2.2 Bacterial Composition Differs Across Firefly Subfamilies, Genera and Species

**FIGURE 3:**
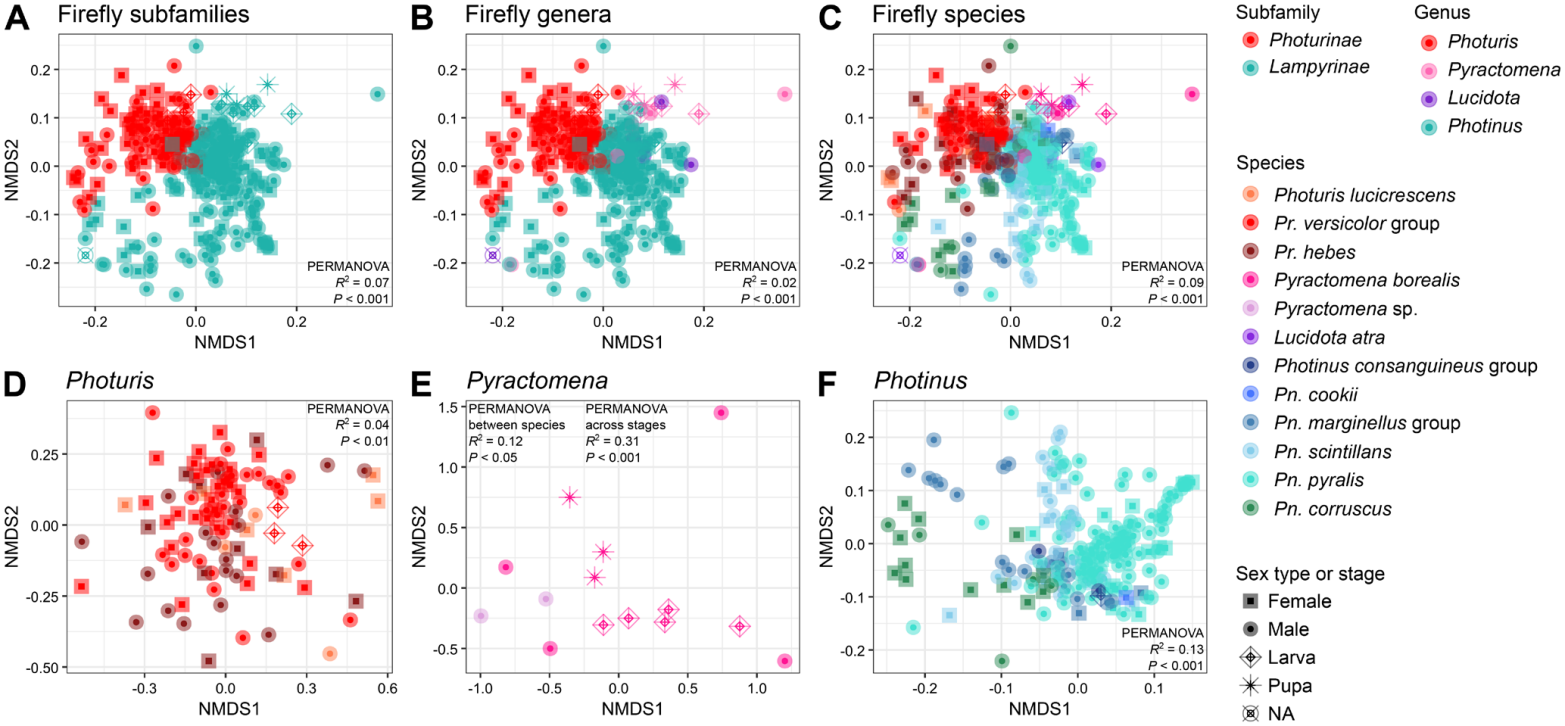
Microbiome divergence across fireflies at different taxonomic levels. (A, B, C) Non‐metric multidimensional scaling (NMDS) analyses showing separation of the bacterial communities based on composition and relative abundance at the firefly subfamily, genus and species levels. Four outgroup specimens were removed from these analyses. The NMDS stress value was 0.262. (D, E, F) NMDS analyses showing variation in the microbiome across species within three firefly genera. The NMDS stress values were 0.276 in D, 0.134 in E and 0.240 in F. All plots are derived from Bray‐Curtis distances calculated on non‐rarefied, proportional (i.e., compositional) relative abundance of taxon‐aggregated bacteria in firefly specimens. Permutational multivariate analyses of variance (PERMANOVAs) were used for statistical testing of group similarities. Nested PERMANOVAs were used for B and C, with genus nested within subfamily and species nested within genus within subfamily, respectively. In panels D and F, the species term was the main predictor in the PERMANOVA models. PERMANOVAs were computed on read depths rarefied at 30,000 reads, except in E where it was computed on non‐rarefied data to be able to compare the two *Pyractomena* species (*Pyractomena* sp. near *sinuata* specimens are filtered out by rarefying) or three developmental stages.

Bacterial composition differed between the *Lampyrinae* and *Photurinae* subfamilies (PERMANOVA: *F*_1,264_ = 22.95, *p* < 0.001; Figure 3A; Table S12) and across genera (nested within subfamilies, PERMANOVA: *F*_2,264_ = 3.87, *p* < 0.001; Figure 3B). Despite high intraspecific variability, bacterial composition also differed significantly across firefly species (nested within genera, within subfamilies, PERMANOVA: *F*_7,264_ = 4.18, *p* < 0.001; Figure 3C–F). To assess whether firefly microbiomes were more divergent in species that were less genetically related, we examined how bacterial community composition varied with degree of host relatedness. Using partial Mantel test controlling for geographic distance, we found a strong positive correlation between pairwise microbiome dissimilarities and host genetic divergence, with closely related firefly species having more similar microbiome composition and abundance (Figure S8), further indicating phylogenetically structured microbiomes in fireflies.

#### 3.2.3 Firefly Species With Bigger Body Sizes and Known to Eat as Adults Tend to Harbour Higher Bacterial Densities

We investigated whether three key characteristics of firefly species—the use of bioluminescence as a mating signal by adults, adult diet and adult body size—influenced firefly microbiomes when taking into account fireflies' degree of relatedness. Through ordinary and phylogenetic generalized least square (OLS and PGLS, respectively) models, we found that bacterial density and richness did not significantly change between bioluminescent and non‐bioluminescent fireflies, although bacterial composition changed marginally (Figure S9; Table S12).

The effect of adult diet was difficult to disentangle in models controlling for phylogenetic relatedness among firefly species because, in our dataset, photurine fireflies were the only predatory taxa and sap/nectar feeding was represented by a single species, thereby confounding dietary and phylogenetic effects. Nevertheless, when not controlling for phylogenetic distance between firefly species, bacterial density was higher in species that eat as adults compared to those not known to eat (OLS: *F* = 16.42, *p* < 0.01; Figure S9), whereas bacterial richness did not vary (Table S12). Bacterial composition was different between eating and non‐eating firefly species and across firefly diets, with the difference being the strongest between non‐eating and the predatory, photurine fireflies (Figure S9; Table S12; Data S1). These results suggest that while fireflies eating as adults may harbour a higher bacterial density and that firefly species diets may influence the composition of their microbiomes, the degree of relatedness is a major factor influencing firefly microbiomes that needs to be accounted for.

We also found that bigger firefly species tend to harbour a higher bacterial density and a lower bacterial richness than smaller species (Figure S10; Data S1). These results show that firefly microbiomes do not appear to be strongly influenced by mating signals, but that species' body size and adult diet, to some extent, may contribute.

### 3.3 Firefly Microbiomes Are Often Dominated by a Few Bacterial Taxa With Signs of Species‐Specific Associations

The most abundant bacterial order associated with all fireflies in our amplicon sequencing dataset was *Entomoplasmatales* (class *Mollicutes*), representing 27.3% of all rarefied bacterial reads from fireflies. Highly represented bacterial orders in fireflies also included *Burkholderiales* (9.5% of all rarefied reads), *Xanthomonadales* (8.6%), *Pseudomonadales* (8.3%), *Enterobacterales* (7.7%) and *Rickettsiales* (7.0%). Compared to non‐lampyrid elateroid beetles, only a few of the abundant firefly‐associated bacterial taxa were similar (Figure S11; Data S1).

#### 3.3.1 Fireflies Harbour a Diversity of Taxa From the *Mollicutes* With Prevalent and Abundant Strains and a Few Endosymbionts

**FIGURE 4:**
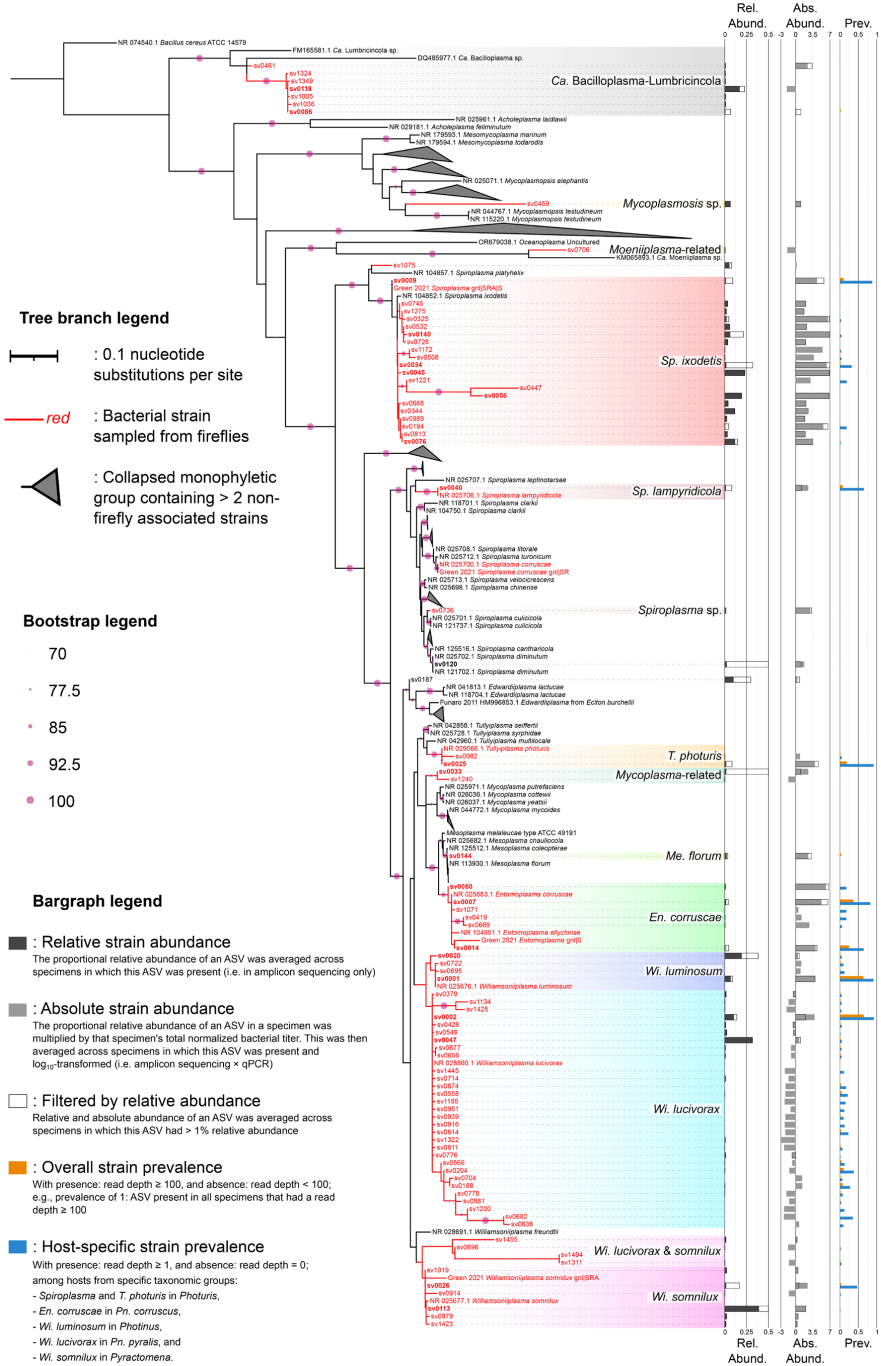
Phylogenetic strain diversity of the *Mollicutes* associated with fireflies. The tree was obtained from a maximum likelihood phylogenetic analysis on the 16S rRNA gene, rooted with a *Bacillus cereus* sequence (NR_074540.1), and contains 230 leaves of which 88 are 16S rRNA amplicon sequences from the present study, four are amplicon sequences from another firefly microbiome study (Green et al. 2021) and four are amplicon sequences from another study on insect‐associated mollicutes (Funaro et al. 2011). All the other leaves are near‐full length 16S rRNA gene sequences that were obtained either through NCBI searches to acquire reference sequences of mollicute species or through NCBI BLASTn runs to find the most similar sequences to our amplicon sequences. Red branches contain bacterial ASVs sampled from fireflies (from the present and previous studies), only focusing on our 1500 most abundant ASVs (SV identifier number lower than 1501). ASV labels in bold are among the most abundant in our dataset (SV identifier number lower than 151). ASV labels in black font correspond to mollicutes obtained from non‐lampyrid beetles (see Figure S11). Grey triangles correspond to collapsed monophyletic groups containing more than two non‐firefly associated leaves. Bootstrap values above 70 are depicted with a pink dot on the tree nodes. Genus and species names are from Gupta et al. (2018, 2019). A detailed version of the tree with uncollapsed leaf labels is shown in Figure S3. The first bargraph on the right with dark grey bars represents ASVs' mean relative abundance (‘Rel. Abund.’) when present, expressed as the proportion of each ASV's non‐rarefied read depth relative to total read depth in a specimen and averaged across specimens that had a read depth > 0 for this ASV. The second bargraph on the right with light grey bars depicts mean absolute abundance (‘Abs. Abund.’) when present, expressed as the proportional relative abundance of each ASV in a specimen multiplied by the specimen's total normalized bacterial titre and averaged across specimens that had both a read depth > 0 for this ASV and a bacterial titre > 0. Black borders correspond to the relative or absolute abundance that were averaged only across specimens in which an ASV was present with at least 1% relative abundance. The last bargraph represents prevalence (‘Prev.’) both across all specimens in our dataset (gold) and within specific firefly groups (blue). Only specimens' whole body was considered for these calculations.

**FIGURE 5:**
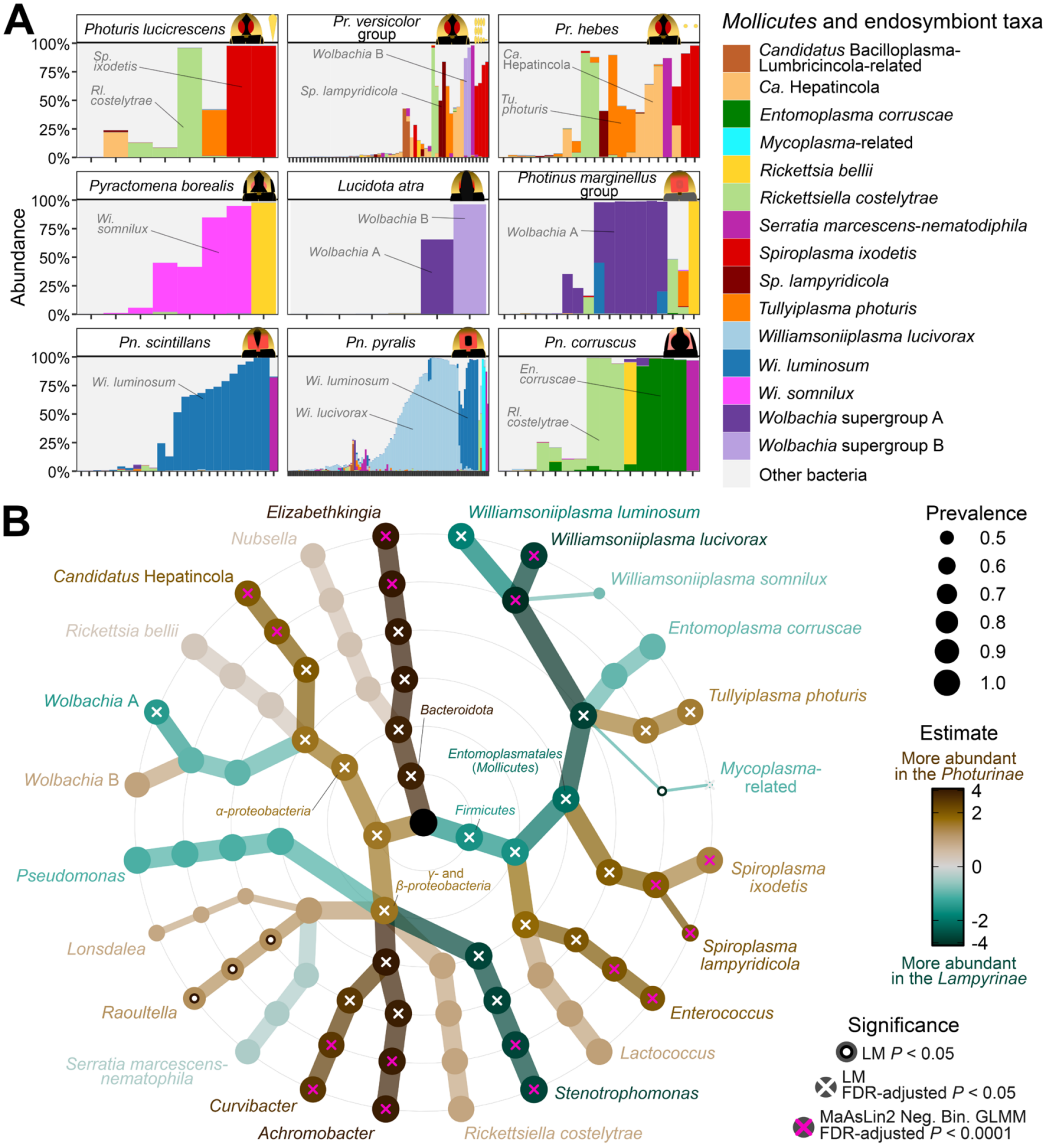
Bacterial composition and differential abundance. (A) Composition of bacterial taxa across firefly species and species groups with more than three specimens in our dataset post rarefying. Each vertical bar (*x*‐axis) represents one firefly specimen. The relative rarefied abundance (*y*‐axis) of the most abundant mollicutes and putative endosymbionts is shown as different colours whereas the abundance of all other bacteria is in light grey and can be seen in Figure S14. (B) Differential bacterial abundance at multiple taxonomic levels across adult fireflies. Only firefly species with more than one specimen in our dataset, post rarefying and only bacterial taxa with rarefied abundance > 20,000 reads in one or more samples were used in this analysis. Moving outwards from the centre, light grey rings show phylum, class order, family and genus (sensu SILVA database); bacterial ‘taxa’ are found on the outermost ring of the tree. The size of the balls depicts prevalence of a clade across samples (proportion of firefly specimens in which it was detected). Results from three statistical tests comparing bacterial abundances in *Lampyrinae* versus *Photurinae* subfamilies are displayed. Following the replacement of zeros by half of the minimum value (to reduce zero‐inflation and improve model fitting) and log_2_ transformation, a linear model (‘LM’) was run on all bacteria at every taxonomic rank using the *microViz* R package. White circles and crosses on balls indicate significantly differential abundance with unadjusted and false discovery rate (‘FDR’)‐adjusted, respectively, at *p* < 0.05. Estimates of bacterial differential abundance from this LM are displayed with shades of brown (darker: Higher abundance in *Photurinae*) and sea green colour (darker: Higher abundance in *Lampyrinae*). At both the bacterial genus and taxon levels, differential abundance was additionally tested through *MaAsLin2* multiple generalized linear mixed models (‘GLMM’) with negative binomial (‘Neg. Bin.’) distribution and controlling for date of capture, geographic origin and sex. Pink crosses show the bacterial taxa that were found to be significantly differentially abundant at FDR‐adjusted *p* < 0.001.

The fireflies we collected harboured strains from almost all the mollicute taxa that have been sampled from fireflies in the past (seven out of eight; Figure 4; Figure S3; Data S1). Both *Spiroplasma ixodetis* and *Williamsoniiplasma lucivorax* species were represented with a high strain diversity in our firefly specimens, with at least 20 and 30 ASVs each, respectively, although only one or two ASVs per mollicute genus or species could be very abundant and prevalent in fireflies, some of which have only been sampled from firefly bodies to date. Several known endosymbionts from other arthropods appeared common in fireflies (Figure 5A; Figures S4, S12 and S13), including bacteria from the *Rickettsia*, *Rickettsiella*, *Wolbachia* and occasionally *Serratia* and *Spiroplasma* genera (Russell et al. 2013; Werren et al. 2008).

#### 3.3.2 *Stenotrophomonas* and *Williamsoniiplasma* Are Highly Prevalent in Lampyrine Microbiomes

The *Stenotrophomonas* (*Xanthomonadales*) and *Williamsoniiplasma* (*Mollicutes*) genera were characteristic members of the microbiome of fireflies from the *Lampyrinae* subfamily (Figure 5B), each detected in more than 98% of lampyrine fireflies (> 57% of lampyrine specimens with more than 1% relative abundance). Compared to photurine fireflies, lampyrines were significantly more associated with bacteria from these two genera (Table S8). While *Stenotrophomonas* could also be detected in *Photuris* fireflies, albeit typically at low abundance, members of the *Williamsoniiplasma* genus were very rare in *Photuris* species (Figure 5A,B; Figures S13 and S14).

Three *Williamsoniiplasma* taxa were commonly detected in the lampyrine *Photinus* and *Pyractomena* genera with differing levels of endemicity (Figure 5A; Figure S13). *Wi. lucivorax* was only found in *Pn. pyralis*, as the most dominant and prevalent bacterium after *Stenotrophomonas*, infecting over 69% of individuals with more than 1% relative abundance, and being significantly more abundant in this firefly species than in any other (Figure S13; Table S8). In contrast, *Wi. luminosum* was detected in both *Pn. pyralis* and *Pn. scintillans*, as well as in two individuals from the *Pn. marginellus* group (Figure 5A; Figure S13), thus appearing less host‐restricted than *Wi. lucivorax*. Yet, *Wi. luminosum* was significantly associated with *Pn. scintillans* (Figure S13), being recovered in all *Pn. scintillans* specimens and 64% of *Pn. scintillans* specimens with 1% relative abundance threshold (Figure 5A). Another species from the *Williamsoniiplasma* genus, *Wi. somnilux*, was significantly more abundant in *Pyractomena borealis* than in other firefly genera (Table S8) and showed endemicity patterns similar to those of *Wi. lucivorax* (Figure 5A; Figure S13). When using the 1% relative abundance threshold, *Wi. somnilux* was present in five out of eight specimens (62%) from this firefly species, including both adults and juveniles, and was the most abundant bacterial taxon (Figure 5A).

Fireflies from the *Pn. corruscus* species were significantly associated with another member of the *Mollicutes*, the *En. corruscae* taxon (Figure 5A; Figure S13), being the most abundant bacterium in all *Pn. corruscus* fireflies, detected at more than 1% relative abundance in nine out of 16 individuals (56%), and showing patterns of endemicity as well. In contrast, four other lampyrine species and species groups rarely harboured mollicutes. Instead, in the *Pn. marginellus* group, *Wolbachia* (*Rickettsiales*) from the supergroup A was the most dominant bacterium (colonizing > 47% of specimens with more than 1% relative abundance).

#### 3.3.3 Photurines Commonly Harbour a Variety of Bacteria, Including a Few Mollicutes

Contrasting with the lampyrines, members of the *Photurinae* were significantly more associated with abundant bacteria from the *Achromobacter* (*Burkholderiales*), *Ca*. Hepatincola (*Rickettsiales*), *Curvibacter* (*Burkholderiales*), *Elizabethkingia* (*Flavobacteriales*), *Enterococcus* (*Lactobacillales*) and *Spiroplasma* (*Mollicutes*) genera (MaAsLin2: coef > 1.87, FDR *p*‐adj < 0.0001; Figure 5B). Despite a lower relative abundance, *Leucobacter* bacteria also appeared significantly associated with photurines (Figures S11, S13 and S14; Table S8). Among the spiroplasmas, two taxa were significantly more abundant in photurine fireflies: *Sp.* *ixodetis* and *Sp.* *lampyridicola* (detected in 14% and 7% of *Photuris* specimens at 1% relative abundance threshold, respectively; Table S8). Another mollicute species, *Tullyiplasma photuris*, was abundant in photurine fireflies, though at lower degrees (Figure 5A; Figure S13), recovered in 13% of *Photuris* specimens at 1% relative abundance threshold.

#### 3.3.4 Other Bacteria May Dominate Firefly Microbiomes Alone or Colonize as a Group

When the endosymbionts *Rickettsia bellii* and *Wolbachia* A and B were harboured by fireflies, both their relative abundance and the specimen's absolute bacterial abundance were very high compared to conspecifics (Figure 5A; Figure S12), making these bacteria largely dominant in these microbiomes, and behaving as rare but potentially overgrowing parasites that can infect multiple species. Along these lines, through our co‐occurrence analysis, we found that these bacteria were more likely to be present on their own in fireflies (Figure S15). *Enterococcus*, *Lactococcus*, *Rickettsiella costelytrae* and *Spiroplasma ixodetis* may also behave as overgrowing parasites depending on the host, sometimes dominating the microbiome, sometimes just being an addition to the community (Figure S12). In contrast, several prevalent bacterial taxa with fluctuating absolute abundance regularly co‐occur within firefly species (Figures S12 and S15). These include *Pseudomonas*, *Stenotrophomonas* and *Rl. costelytrae* in both *Pn. marginellus* group and *Pn. pyralis*, and *Achromobacter*, *Curvibacter* and *Elizabethkingia* in *Photuris* fireflies. This suggests that, beyond the aforementioned near‐consistent presence of mollicutes, fireflies can also harbour either densely infecting endosymbionts that are more likely to occur alone or groups of more common bacterial taxa typical of gut microbiota.

### 3.4 Within‐Species Microbiome Variation Can Be Partially Explained by Geography, Season, Environment, Sex and Development

#### 3.4.1 The Abundance of Some, but Not All, Bacteria Vary Across Geographic Locations

To assess whether geography was a main factor influencing changes in firefly microbiomes, we first used partial Mantel tests on microbiome dissimilarity matrices and geographic distance, while taking into account species' phylogenetic distances. Through this test, we found a weak positive correlation between pairwise microbiome dissimilarities based on relative bacterial abundance and geographic distance (Table S12), indicating that regardless of host species identity, fireflies separated by longer spatial distances tend to harbour more dissimilar microbial communities. We next investigated within‐species microbiome variation for species that were collected in multiple regions.

Depending on the firefly species considered, total absolute bacterial abundance could either remain stable across specimens collected in different regions, such as in *Pn. marginellus* group, *Pn. pyralis* and *Pr. hebes* (emmeans: *z*‐ratio < 1.29, *p* > 0.057) or fluctuate, as in *Pn. corruscus*, *Pn. scintillans*, and unidentified species of the *Pr. versicolor* group (emmeans: *z*‐ratio > 2.03, *p* < 0.05). At the level of the whole bacterial community, while *Pn. corruscus*, *Pn. marginellus* group, *Pn. scintillans* and *Pr. hebes* microbiomes did not differ across regions (PERMANOVA: *R* ^2^ < 0.40, *F* < 1.33, *p* > 0.11), the bacterial composition of *Pn. pyralis* and unidentified species of the *Pr. versicolor* group showed clear variation (PERMANOVA: *R* ^2^ = 0.13, *F* > 1.83, *p* < 0.001; Figure S16; Table S12).

**FIGURE 6:**
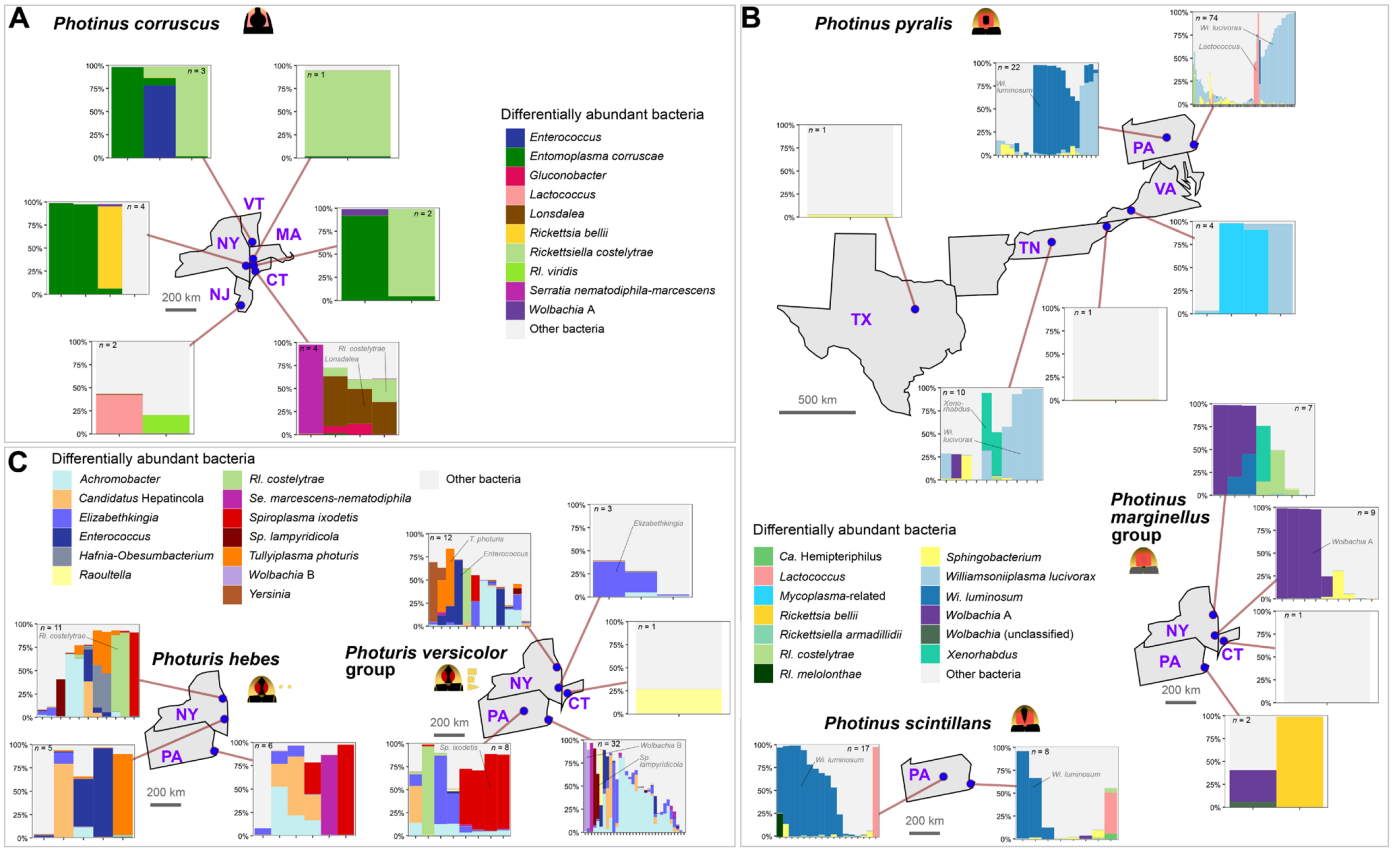
Regional differences in bacterial composition. Bacterial composition, with a focus on the most differentially abundant bacterial taxa across regions. Firefly species are grouped by microbiome similarity, with *Photinus corruscus* in (A), *Pn. pyralis*, *Pn. marginellus* group and *Pn. scintillans* in (B) and *Photuris hebes* and unidentified species from the *Pr. versicolor* group in (C). Bacterial taxon composition and abundance per species per region were obtained from rarefied read depth. The number of specimens is given within each bar plot. In all panels, CT, Connecticut; MA, Massachusetts; NJ, New Jersey; NY, New York; PA, Pennsylvania; TN, Tennessee; TX, Texas; VA, Virginia; VT, Vermont US states. A full list of differentially abundant bacterial taxa across regions can be found in Table S9.

We observed important regional differences at the level of individual bacteria (Figure 6A; Table S9; Data S1). *En. corruscae*, a characteristic bacterial taxon of *Pn. corruscus*, may be preferentially associated with specimens from northern latitudes, since it was less abundant in specimens collected in southwestern Connecticut and South New Jersey compared to specimens collected in northwestern Connecticut, southeastern New York (seNY) and southwestern Vermont (Table S9). For *Pn. pyralis*, the most divergent microbiomes were from specimens collected in central Pennsylvania (cPA) (pairwise adonis: *R* ^2^ > 0.08, *F* > 2.99, FDR *p*‐adj. < 0.05; Figure 6B). Likely contributing to the singularity of cPA *Pn. pyralis* microbiomes, *Wi. luminosum* was mostly present in individuals collected in this region (MaAsLin2: coef > 3.64, FDR *p*‐adj < 0.05; Table S9), where its prevalence at 1% relative abundance threshold was 68% (64% for *Wi. lucivorax* in these specimens) versus 4% in *Pn. pyralis* from all other regions (71% for *Wi. lucivorax* in these other specimens).

Regarding the *Photuris* genus, members of the bacterial taxon *Sp.* *ixodetis* were common and abundant in specimens collected in cPA, but were very rare in specimens from other regions (Table S9). We also identified regional specificities among other common bacterial associates of *Photuris* fireflies, with *T. photuris* being more abundant in northeastern NY (neNY) and seNY compared to southeastern PA (sePA) for *Pr. hebes*, and neNY compared to sePA in unidentified species from the *Pr. versicolor* group.

#### 3.4.2 Seasonal, but Not Annual, Factors Influence Within‐Species Microbiome Variation

We tested whether seasonality, which is linked to firefly adult age, influences firefly microbiome. When focusing on all species in our dataset, bacterial density varied throughout the day of year (GLMM Wald test: *Χ* ^2^ = 124.38, *p* < 0.0001; Table S12), but only for some firefly species, including *Pn. scintillans*, unidentified *Pr. versicolor* group and *Py. borealis* which had higher bacterial titers later in the season (emmeans: *z*‐ratio > 2.63, *p* < 0.05). Bacterial composition also varied throughout the year and across species (PERMANOVA with species and regions as covariates, focusing on the interaction between day of year and species: *R* ^2^ = 0.05, *F*_14,186_ = 1.20, *p* < 0.05). We focused our next analyses only on *Pn. corruscus* specimens given their long adult lifespan (up to 10 months versus < 3 months usually for other species) and since we collected this species throughout the year.

**FIGURE 7:**
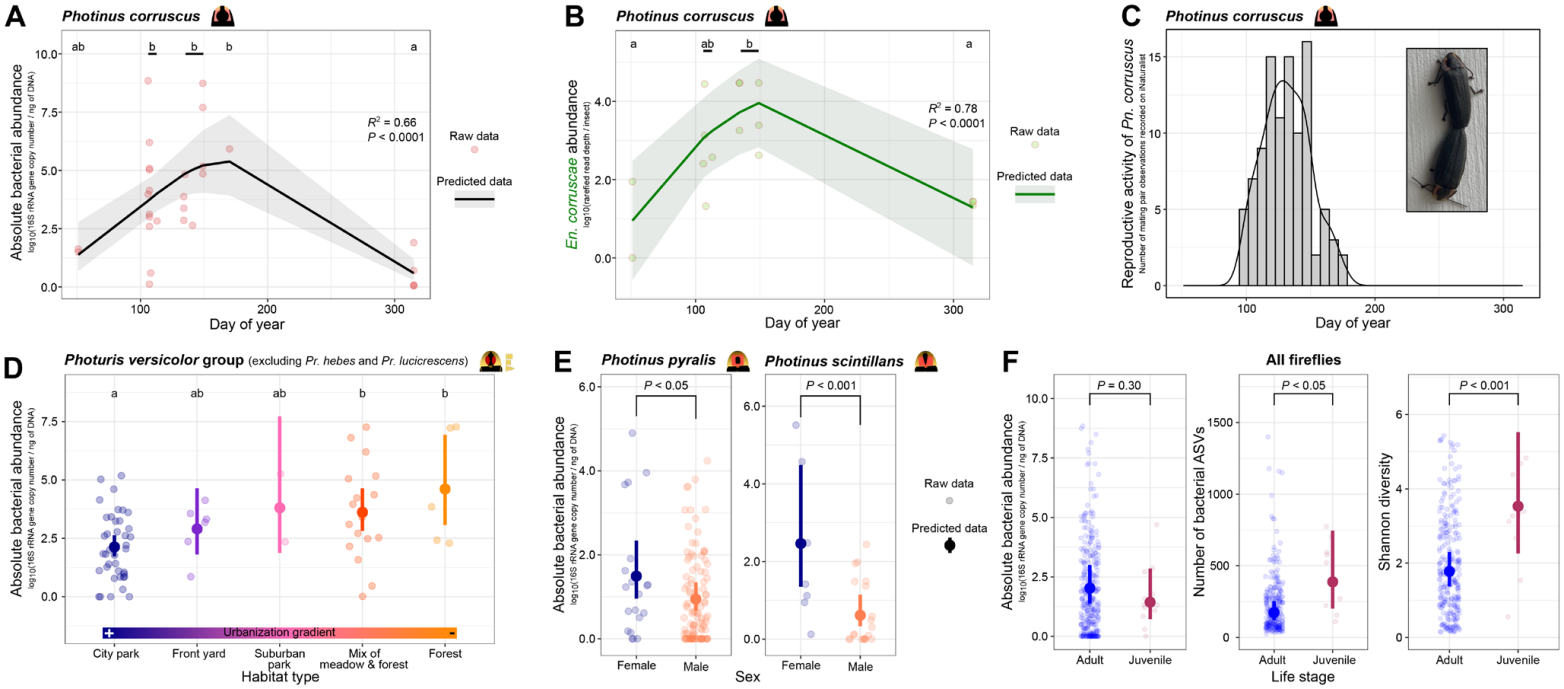
Seasonal, environmental and life history factors influence the microbiome of fireflies. (A) Normalized absolute bacterial abundance in *Photinus corruscus* peaks at the end of spring. A generalized linear mixed model (GLMM) was used to test whether bacterial titre is influenced by day of year, used as a quadratic term, in *Pn. corruscus*, when controlling for sex, latitude, year of capture, and whether the specimen was dissected or not. Change in bacterial titers over the months for this species was also tested with least‐square means (emmeans) post hoc test on a GLMM controlling for the same factors. Significant differences between February, April, May, June and November are shown with lowercase English letters. (B) Abundance of *Entomoplasma corruscae* in *Pn. corruscus* also peaks at the end of spring. *En. corruscae* abundance is expressed as the log_10_ of *En. corruscae* read depth per firefly specimen in our rarefied amplicon sequencing dataset. A GLMM was used to test whether *En. corruscae* abundance is influenced by day of year, used as a quadratic term, in *Pn. corruscus*, when controlling for sex, region of capture, year of capture and specimen identifier. Lowercase English letters indicate significant differences between months, as measured in the previous panel. (C) Reproductive activity in *Pn. corruscus* also peaks in the spring. The bars depict the number of mating pairs observed per week on iNaturalist for this species in Connecticut, New Jersey, New York, Pennsylvania and Vermont, in 2021, 2022 and 2023. The photograph of a type 1 copulation event (Wing 1984) was taken in New York state in 2021 by Benoît Béchade. (D) For unidentified species from the *Photuris versicolor* group, the highest bacterial densities are found in specimens from the least urbanized habitats. A GLMM was used to test whether bacterial titre is influenced by habitat type in these species, when controlling for sex, latitude, year and time of capture, day of year, specimen identifier, and whether the specimen was dissected or not. Lowercase English letters indicate significant differences across habitat types. (E) Bacterial absolute abundance is higher in females than in males of *Pn. pyralis* and *Pn. scintillans*. A GLMM was used to test whether bacterial titre is different between females and males in firefly species (i.e., through the interaction term sex: Species), when controlling for the factors as in the previous panel. (F) Firefly juveniles harbour a higher bacterial diversity than adults, but the two life stages do not differ in their absolute bacterial abundance. GLMMs were used to test whether bacterial titre, bacterial richness and the bacterial Shannon diversity index are different between juveniles (including larvae and pupae) and adults, using firefly genus as a covariate and controlling for region, day of year and year of capture. For panels A, D, E and F, bacterial titre is expressed as the log_10_ of the number of 16S rRNA gene copies in a sample, measured through qPCR, minus the number of 16S rRNA gene copy in the DNA extraction blank sample from the same batch, and then divided by the total DNA concentration in the sample, which was measured via Qubit. For all panels except C, predicted data (curve for A and B and balls for D, E and F) with 95% confidence interval (grey shade for A, B and C and error bars for D, E and F) were obtained from GLMMs.

*Pn. corruscus* specimens collected in the months of April, May and June had higher bacterial titers than those collected in November (Figure 7A; Table S12). At the level of individual bacterial taxa, specimens collected in May and April were typically associated with high relative abundance of *En. corruscae*, but this bacterial taxon was barely detectable in *Pn. corruscus* specimens collected in February and November (Figure 7B; Table S10). Supporting these results, we detected a strong association between the collection day of year used as a quadratic continuous predictor in our LMMs and both total bacterial titers and abundance of *En. corruscae*, with a peak in late May (Figure 7A,B). Using iNaturalist citizen‐science records (INaturalist Community 2025), we found that in the years and states sampled, the reproductive activity of *Pn. corruscus* ran from early April to mid/late June, peaking in May (Figure 7C). This corresponds to the reproductive seasons observed for this species in other states and years (Faust 2012; Rooney and Lewis 2000; Smedley et al. 2017), and implies a potential link between *En. corruscae* abundance and either its host reproduction or seasonal increased food intake (Rooney and Lewis 2000).

To assess microbiome turnover across years, we collected *Pn. pyralis* specimens in the same location, at approximately the same time of year, for three consecutive years. The overall microbiome composition of *Pn. pyralis* changed only marginally over the years (PERMANOVA: *R* ^2^ = 0.06, *F*_2,53_ = 1.76, *p* = 0.051; Table S12). At the level of individual bacterial taxa, while the abundance of *Wi. lucivorax* remained stable from 2021 to 2023, that of *T. photuris* and *Wi. luminosum* increased and that of *Pseudoclavibacter* and *Tumebacillus* dropped (Figure S17; Table S10). This shows that the overall composition of bacteria associated with fireflies may not drastically change over the years, although the abundance of a few bacteria may fluctuate.

#### 3.4.3 The Microbiome of Fireflies From the *Photuris versicolor* Group Changes Across Habitat Types

The interaction between species and habitat types (with different urbanization levels) significantly affected both bacterial composition and density (Table S12), indicating that different species' microbiomes vary in their response to divergent habitat types. In unidentified species from the *Pr. versicolor* group, absolute bacterial abundance was significantly lower for specimens collected in the city than in the forest or in a mix of forest and meadow (Figure 7D). *Rickettsiella costelytrae* may have been a key influence on bacterial titers over the urbanization gradient since it was more abundant in specimens from the forest than in the city (Figure S18; Table S10). These results show that the type of habitat used by *Photuris* fireflies may influence the abundance and composition of their associated bacteria.

#### 3.4.4 *Photinus* Females Harbour a Denser Bacterial Community Than Males

Using qPCR, we found that females of both *Pn. pyralis* and *Pn. scintillans* had a significantly higher bacterial titre than their male conspecifics (Figure 7E; Table S12). In contrast, bacterial evenness was higher for males compared to females in the *Photinus* genus (Figure S19), suggesting that bacterial strains in male microbiomes were represented in more equal proportions, while female microbiomes tended to be dominated by fewer bacteria. Nevertheless, firefly microbiome composition did not significantly differ between sexes, with the only exception of *Pr. hebes* (pairwise adonis: *R* ^2^ = 0.09, *F* = 1.97, FDR *p*‐adj < 0.05).

#### 3.4.5 Juveniles Harbour a More Diverse Bacterial Community Than Adults

We collected a few larvae from three different firefly genera, and pupae from the *Pyractomena* genus. When comparing adults to juveniles (i.e., including both larvae and pupae) from all firefly species, while we detected no difference in terms of absolute bacterial abundance, there was a significant difference in their microbiome composition (PERMANOVA with life stage nested within species within genus within subfamily: *R* ^2^ = 0.01, *F*_3,261_ = 1.35, *p* < 0.05), and a higher bacterial richness and diversity in juveniles compared to adults (Figure 7F; Table S12). These results appear to be driven by the difference between larvae and adults (Figure S20; Data S1).

Juveniles from the *Py. borealis* species did not have a significantly different bacterial community as a whole when compared to adult males from the same species (Figure S21; Table S12). Nevertheless, and despite our low sample size, significant differences in the abundance of single bacteria across larvae, pupae and adult males could be noted. The mollicute *Wi. somnilux* associated with adults and larvae at similar abundances, but was present in lower abundance in pupae (Figure S22; Table S10). Other bacterial genera, including *Rhodococcus*, had a significantly higher abundance in *Pyractomena* juveniles compared with adult males. These results indicate that juveniles harbour a more diverse microbiome, and some of the most prevalent and abundant adult‐associated bacteria are also abundant in larvae and detected in pupae.

### 3.5 Bacterial Tissue Localization Varies Across Firefly Species

We investigated preferential colonization of bacteria in specific tissues of three firefly species and species groups through dissections and tissue separations (Table S2). Our analyses uncovered a significant difference in overall bacterial composition across firefly tissues, but only for unidentified species from the *Pr. versicolor* group, between their lantern and gut tissues (pairwise adonis: *R* ^2^ = 0.12, *F* = 2.36, FDR *p*‐adj < 0.01; Figure S23; Table S12), indicative of major tissue differences only in *Photuris* fireflies.

#### 3.5.1 *Pn. corruscus* Tissues Do Not Differ in Bacterial Titre but Have a Few Differentially Abundant Bacterial Taxa

**FIGURE 8:**
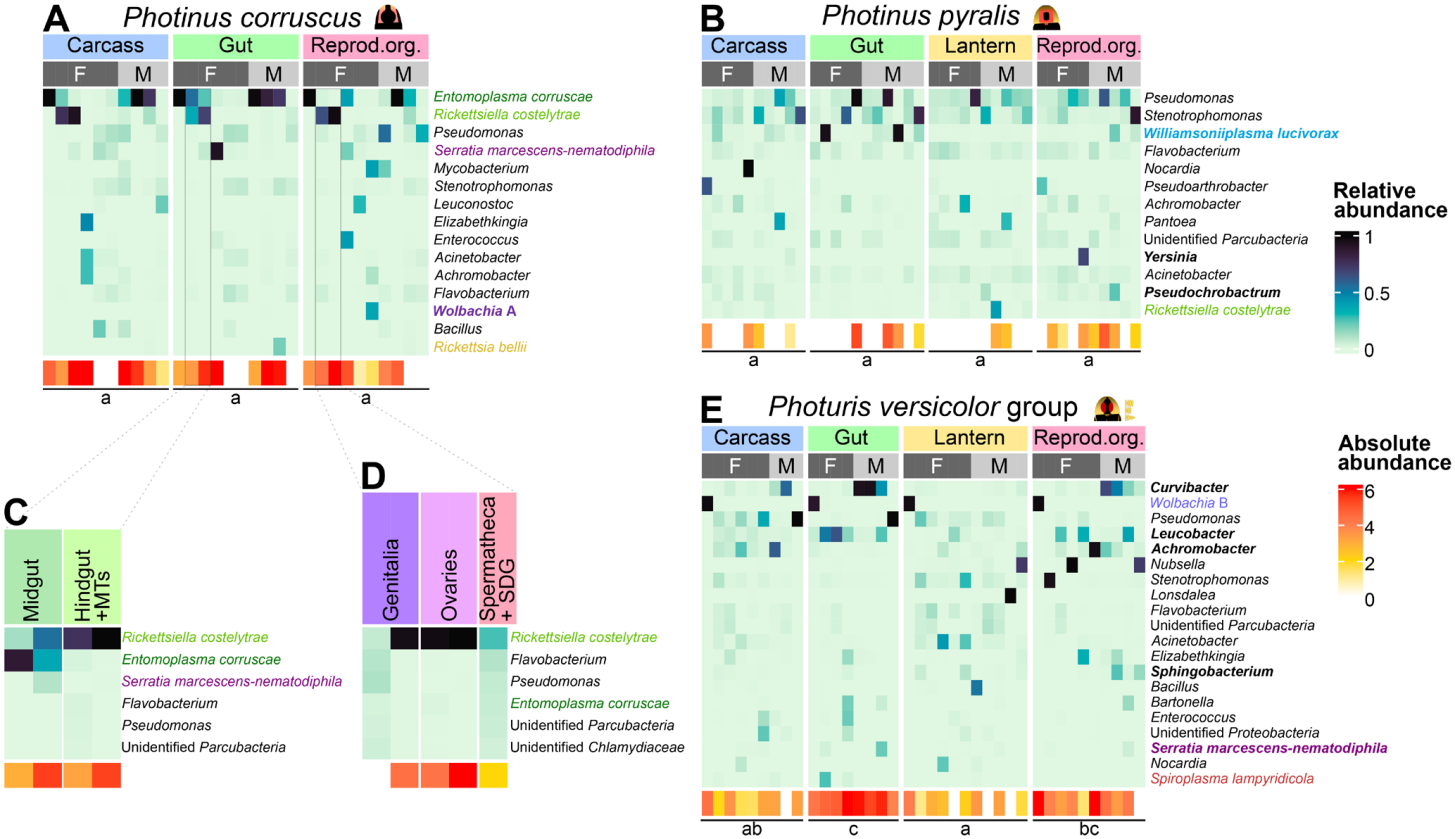
Microbiome changes across tissues in three firefly species. (A, B, E) Bacterial community composition and abundance across the carcass, gut, reproductive organs (‘Reprod. Org.’) and lantern in *Photinus corruscus*, *Pn. pyralis* and unidentified species from the *Photuris versicolor* group. Blue shaded heatmaps show the relative abundance of the most abundant bacterial taxa for each tissue per specimen. The abundance was calculated from non‐rarefied, proportional (i.e., compositional) transformations of read depth relative abundance. Bacterial taxa with bold fonts are significantly differentially abundant between at least two tissues in the species, as tested through *MaAsLin2* multiple generalized linear mixed models (GLMMs), using the cumulative sum scaling normalization method, controlling for geographic location and date of sampling, and using the FDR‐adjusted *p* < 0.05 as significance threshold. All the differentially abundant bacterial taxa across tissues can be found in our Table S11. Red shaded heatmaps show non‐normalized absolute bacterial abundance, expressed as the log_10_ of the number of 16S rRNA gene copies in a sample, measured through qPCR, minus the number of 16S rRNA gene copy in the dissection blank sample, minus the number of 16S rRNA gene copy in the DNA extraction blank sample from the same batch. A linear mixed model (LMM) was used to compare bacterial titre across tissues in these species, by using the species: Tissue interaction term as the fixed effect, controlling for sex, latitude, day of year, year of capture and temperature. For each firefly species, lowercase English letters indicate significant difference in absolute bacterial titers across tissues, as obtained from emmeans post hoc tests on the LMM. (C, D) Total absolute bacterial abundance and relative abundance of the six most abundant bacteria in separated gut compartments and reproductive organs of two *Pn. corruscus* female specimens. MTs. Malpighian tubules; SDG, Spermatophore‐digesting gland. More detailed heatmaps and dissection pictures can be found in Figures S24–S26, and dissection information can be found in Table S2.

Among *Pn. corruscus* specimens, bacterial densities did not vary across tissues (Figure 8A). In terms of composition, while *En. corruscae* was not detected in every specimen, it could colonize all the main tissues of both males and females in high abundance.

Two females had their gut segments and reproductive organs separated through dissection. In these specimens, *En. corruscae* was not detected in ovaries but was present in other reproductive organs, and was very abundant in the midgut (Figure 8A,C,D; Figure S24). In contrast, while *Rl. costelytrae* was not harboured by every specimen, it was very abundant in the carcass, hindgut and ovaries of the two *Pn. corruscus* females dissected, and could also be detected in their genitalia, spermatheca and spermatophore‐digesting gland, and midgut, matching infection patterns typical of symbionts residing inside host tissues (i.e., endosymbionts) (Perlmutter and Bordenstein 2020). Mollicutes and endosymbionts may thus slightly differ in their tissue localization when they associate with *Pn. corruscus*, with *En. corruscae* being mainly present in the midgut but also infecting reproductive organs, and *Rickettsiella* colonizing most tissues, including ovaries.

#### 3.5.2 Bacteria Variably Colonize the Tissues of *Pn. pyralis* but With a Potential Primary Gut Localization of Mollicutes

While there were no significant differences in bacterial titers across *Pn. pyralis* dissected tissues (Figure 8B), the gut and reproductive organs could occasionally be infected with high titers. When present, *Wi. lucivorax* was relatively abundant in the gut, and while it was sometimes detected in the carcass and reproductive organs of males, it appeared virtually absent from the lantern (Figure 8B; Figure S25; Table S11). This suggests that, as for *Pn. corruscus*, mollicutes may have a primary gut localization in *Pn. pyralis*, but with lower bacterial titers overall in this firefly species.

#### 3.5.3 Specimens From the *Photuris versicolor* Group Harbour High Density Gut Bacteria

While bacterial density in unidentified species from the *Pr. versicolor* group whole body was relatively high (Figure 1C; Figure S8), it peaked in the gut, especially compared to the lantern and carcass (Figure 8E; Figure S26; Table S12). All individuals from these species had a consistently high bacterial titre in the gut, regardless of sex, collection date and location. Reproductive organs also had significantly higher bacterial titers than the lantern (Table S12).

When separating the unidentified *Pr. versicolor* group specimens by sex, bacteria from the *Curvibacter* genus dominated the microbiome of male guts and reproductive organs compared to female guts and other male tissues (Figure 8E; Table S11). Otherwise, the gut of both females and males included a high abundance of *Leucobacter* in the gut and reproductive organs compared to the carcass and the lantern (Table S11). Notably, one female harboured *Wolbachia* from the supergroup B in all its tissues and another one had a high abundance of *Sp.* *lampyridicola* in its gut (Figure 8E). *Photuris* fireflies thus harbour high density gut bacteria with compositions that seem to differ between males and females.

## 4 Discussion

We studied the factors shaping firefly microbiomes through a comprehensive characterization of bacterial communities across 12 Northeastern US firefly species. Firefly microbiomes were marked by high variability in bacterial composition and abundance, with a background of low bacterial abundance occasionally punctuated by bacterial proliferations (Figures 1C,D, 5A, 6 and 8; Figures S6, S12 and S21). Despite this intraspecific variability, most firefly species harboured distinct bacterial communities (Figure 3C–F), implying that each host species preferentially associates with its own set of bacteria. Consistent with this, normalized stochasticity ratios (NST) analyses indicated that either or both mollicutes and/or endosymbionts were acquired by fireflies through deterministic factors and often dominated microbiomes when present, whereas the remaining bacterial community was assembled largely through stochastic forces and consisted predominantly of low‐abundance, likely transient taxa (Figure 2; Figure S5). These results highlight the importance of partitioning microbiome analyses based on microbial incidence (e.g., *Mollicutes*) and known functional groups (e.g., endosymbionts), as different ecological processes dictate their acquisition and maintenance by hosts.

### 4.1 Ecological Mechanisms Structuring Firefly Microbiota

Deterministic processes—including habitat/host filtering mechanisms, vertical transmission and microbe‐microbe interactions—appear to shape the retention of mollicutes and certain endosymbionts in fireflies, whereas the remaining bacterial community assembles largely through stochastic exposure. Such duality mirrors patterns seen across animal microbiomes (Chandler et al. 2011; Furman et al. 2020; Hendrycks et al. 2025; Oliphant et al. 2019), where ecological drift (random variation in community structure due to births, deaths and dispersal) governs transient microbial taxa while host‐associated mechanisms stabilize persistent symbionts. Our results provide evidence for each of these assembly processes in fireflies.

Geographic and habitat‐associated variations in bacterial prevalence support stochastic microbial acquisition from local environmental pools. The presence of bacterial taxa such as *Lactococcus* varied across *Photinus* specimens collected in different geographic locations (Figure 6A–B), and *Photuris* microbiomes differed across habitats (Figure 7D; Figure S18). Such intraspecific variability of microbiome composition has been described in a wide range of hosts from butterflies to mammals (Ley et al. 2008; Ravenscraft et al. 2019), and likely reflects unpredictable exposures to environmental microbial pools, which are themselves known to differ in their composition across space (Hosen et al. 2017; Malard et al. 2019; Martiny et al. 2006; Zheng et al. 2024). Stochasticity also plays a major role in the infection with potentially parasitic bacteria (Hussein et al. 2026). *Rickettsia bellii* , which is a known insect parasite (Liu et al. 2024), changed fireflies' whole‐body microbiome and could be present in very high absolute abundances (Figure S12), suggesting advanced levels of infection. Similarly, although in a host species‐dependent manner, *Enterococcus*, *Lactococcus* and *Rickettsiella costelytrae* could occasionally overwhelmingly dominate microbiomes, suggesting their populations might burst when infecting fireflies.

Contrasting with stochastic factors, persistent differences in microbiome composition among sympatric firefly species strongly suggest deterministic host filtering, with host physiology and/or microbial traits likely dictating the establishment of characteristic bacterial taxa. Despite being collected at the same locations, times of day and seasons, sympatric firefly species did not share exactly the same microbiomes, whether they were closely related or not (Figures 3A–F and 6A–C). As previously pointed out by Hackett et al. (1992), even fireflies from the *Photuris versicolor* group, which prey on *Photinus* and *Pyractomena* fireflies, did not harbour any of the *Williamsoniiplasma* bacteria characteristic of their prey (Figure 5A,B). In addition, several firefly‐associated bacterial taxa from the *Mollicutes* may be endemic to fireflies since they have only been detected in firefly tissues to date (Figure 4; Figures S11 and S13). These results are indicative of host restriction, suggesting that fireflies are not entirely permeable to the microbes they are exposed to but instead selectively retain specific bacterial associates.

Vertical transmission is a special case of deterministic habitat/host filtering mechanism, where only specific symbionts are transmitted from parents to offspring and conserved by the host line throughout generations. Many insects harbouring lineage‐specific, obligate symbionts have been shown to rely on strict vertical transmission to preserve key beneficial functions from the symbiont (Baumann 2005; McCutcheon et al. 2009; Sacchi et al. 2000). Yet, several endosymbionts that are common across arthropods, such as *Rickettsia*, *Rickettsiella*, *Spiroplasma* and *Wolbachia*, practice a mix of vertical and horizontal transmission with no strict host specificity (Gu et al. 2023; Liu et al. 2024; Michalik and Szklarzewicz 2025; Perreau and Moran 2022). Here, we confirmed the endosymbiotic nature of *Rickettsiella* and *Wolbachia* in fireflies, which infected several tissues, including reproductive organs (Figure 8A–E). While our NST analysis suggests that the acquisition of *Spiroplasma* and *Wolbachia* was deterministic in unidentified species from the *Pr. versicolor* group and *Pn. marginellus* group, respectively (Figure 2), these bacteria had a more random distribution in other firefly species (Figure 5A), as did *Rickettsia* and *Rickettsiella* (Figure S5), suggesting a stochastic influence, perhaps conditioned by idiosyncratic horizontal jumps across hosts. Although we detected endosymbionts and mollicutes in female reproductive organs (Figure 8A), it is still unclear whether they are vertically transmitted to firefly offspring and whether they play a role in reproductive manipulation, as many endosymbionts do (Doremus and Hunter 2020).

Microbe‐microbe interaction is another deterministic factor shaping microbiomes through the exclusion or facilitation of other bacteria's establishment. When they were detected in fireflies, *Rickettsia* and *Wolbachia* were each likely to dominate bacterial communities and rarely co‐occurred with other bacteria Figure S15, which could be a consequence of competitive exclusion driven by these known endosymbionts (Caragata et al. 2013). It is also possible that these endosymbionts were present at such high densities in firefly tissues that the sequencing reads of other co‐occurring bacteria were barely detectable (Gofton et al. 2015). Conversely, patterns of positive co‐occurrence among bacteria associated with firefly species may suggest facilitation. Chief among them is the *Pseudomonas*‐*Stenotrophomonas* duo found in several *Photinus* fireflies (Figure S15), also known to cooperate during infections of vertebrates (McDaniel et al. 2020, 2023). Future work should investigate whether these bacteria are acquired together by chance (Carpenter et al. 2021), or truly facilitate each other's establishment (Halliday et al. 2024; Zélé et al. 2018) in firefly hosts, and should experimentally test the contribution of each of these stochastic and deterministic mechanisms in structuring firefly microbiota.

### 4.2 Which Factors Influence the Association With Bacterial Symbionts?

Host ecological and life history traits can modulate the balance between deterministic and stochastic microbiome assembly. Although adult firefly bioluminescence showed no consistent microbial signature (Figure S9), diet, seasonality and life stage each influenced bacterial abundance and composition in distinct ways.

Fireflies that feed as adults harboured markedly different microbiomes from those that do not (Figure S9), consistent with the strong influence of diet on animal microbiomes (Degregori et al. 2024; Karasov and Douglas 2013). However, this pattern largely disappeared after controlling for phylogenetic relatedness, which explained much of the interspecific microbiome dissimilarity (Figure 3A–C; Figure S8). Still, contrasting microbiomes in closely related, sympatric species such as *Pn. pyralis* and *Pn. corruscus*—which differ in adult diet, lifespan and reproductive season—suggest that ecological differences can override phylogenetic proximity. This highlights the need for dietary analyses that explicitly separate the effects of host relatedness and ecology on microbial assembly, as well as control for time since last meal which is a main influence on gut microbial density and activity (Estrela et al. 2025; Yang et al. 2021).

Seasonal patterns likely interact with diet (e.g., food availability) and reproduction (e.g., sexual maturity) to shape bacterial abundance. In the long‐lived, adult *Pn. corruscus*, densities of the mollicute *Entomoplasma corruscae* peaked in spring, coinciding with high reproductive activity (Figure 7B–C). Given that this bacterium was sometimes abundant in the midgut but could also be detected in the reproductive organs and carcass (Figure 8A,D; Figure S24), several acquisition routes are possible. First, *Pn. corruscus* may drastically increase food intake during the reproductive season (Rooney and Lewis 2000), thereby acquiring large quantities of this bacterium from food sources—consistent with an environmentally driven, stochastic acquisition mode. Second, as in aphids that transmit facultative endosymbionts during mating (Moran and Dunbar 2006), *En. corruscae* may be sexually transmitted, given its occasional presence in reproductive organs—reflecting a horizontal, stochastic transmission route. Finally, *En. corruscae* may be vertically transmitted from parent to offspring, although this possibility was not supported by a recent study of six *Pn. corruscus* eggs (Green et al. 2021).

Across firefly development, certain bacterial taxa appear to persist through metamorphosis, hinting at a possibly strong deterministic mechanism for microbial maintenance. Larvae harboured a more diverse bacterial community than adults (Figure 7F; Figure S20), with diversity indices and several genera similar to those reported from *Aquatica leii* firefly larvae in China (Zhao et al. 2023). This elevated diversity likely reflects environmental acquisition from the microbe‐rich microhabitats firefly larvae inhabit, such as soil (Torsvik et al. 1990). Yet, several bacterial taxa were retained across life stages. *Py. borealis* larvae from distant localities consistently harboured *Wi. somnilux* (Figures S21 and S22), suggesting deterministic maintenance. The detection of the same bacterium at low abundance in a near‐emergence pupa (Figures S21 and S22), and its previous isolation from *Py. angulata* pupae (Hackett et al. 1992; Williamson et al. 1990), supports the idea that mollicutes can survive metamorphosis, at least in *Pyractomena* fireflies. Although microbial abundance typically declines sharply during the pupal stage in holometabolous insects (Hammer and Moran 2019), symbiont retention through metamorphosis has been observed. For example, the fruit fly *Drosophila melanogaster* maintains the mollicute *Spiroplasma* through pupation (Anbutsu and Fukatsu 2003). Together, these findings reveal that while much of the firefly microbiome is shaped by transient, environmentally acquired bacteria, several mollicute taxa persist across geographical gradients, seasonal changes and developmental transitions, underscoring their deterministic, host‐specific association with fireflies.

### 4.3 Insights Into the Evolutionary History of Firefly‐Associated Mollicutes

Despite their prevalence and apparent host restriction, firefly‐associated mollicutes show little evidence of long‐term co‐diversification with their hosts based on phylogenetic and phylogenomic inferences (Figure 4; Figure S3) (Gupta et al. 2019; Lo et al. 2018), as mollicutes infecting closely related firefly species are rarely each others' closest relatives. For instance, the closely related fireflies *Pn. corruscus* and *Pn. pyralis* commonly harboured *En*. *corruscae* and *Wi. lucivorax*, respectively, from two genera that are phylogenetically separated by other bacterial genera with very different lifestyles (e.g., members of the *Mycoplasma* are vertebrate pathogens) (Fischer et al. 2012; Waites and Talkington 2004). Even within mollicute genera, the preferred host can vary. For example, the *Williamsoniiplasma* genus includes species associated with either lampyrine fireflies or tiger beetles (although very rare) (Tully et al. 1998), which last shared a common ancestor over 300 million years ago (McKenna et al. 2019). It is thus unlikely that the association with mollicute symbionts is the result of ancient domestication in a common host ancestor and conservation through firefly evolution to extant lineages, but two non‐exclusive evolutionary scenarios may explain firefly‐mollicute phylogenetic incongruences.

First, species from the *Mollicutes* may be generalists and associated with multiple host species. These bacteria could switch hosts on multiple occasions over evolutionary time scales, a process that has been documented in other insect‐symbiont systems (Sudakaran et al. 2017). While two *Williamsoniiplasma* species each appear to specifically associate with one lampyrine species or genus (Figure 5A), the *Wi. luminosum* species was detected at moderate to high abundance in three *Photinus* species (Figure 5A; Figure S13) (Fallon et al. 2018; Williamson et al. 1990), suggesting a lower level of host specificity. This is also supported by *En. ellychniae*, a bacterial strain with a 16S rRNA amplicon sequence almost identical to *En. corruscae* (Figure 4), being able to infect *Pn. corruscus* as well as tabanid flies (Wedincamp Jr. et al. 1996); and by members of the *Spiroplasma* genus, with single *Spiroplasma* species being detected in different arthropod lineages (Hackett et al. 1992, 1996; Stevens et al. 1997; Tully et al. 1995). Host switching could conceivably be facilitated by interspecific mating behaviours, which have been documented among *Photinus* fireflies (Lloyd 1967; Vergara et al. 2025). Future microbial transplantation experiments could help discern the extent of species‐specificity versus host‐switching in firefly‐mollicute associations.

A second possible explanation for the discrepancy in firefly‐mollicute phylogenies is that some of these associations may have evolved recently, leading to patterns of co‐phylogeny that may only be detectable at lower taxonomic levels, within single mollicute genera, across species or strains. The *Entomoplasma* and *Williamsoniiplasma* genera, for example, may each include additional firefly‐associated symbiont species that have to be discovered from yet unscreened firefly species. Given that *Williamsoniiplasma* species can be endemic to single firefly host species (e.g., *Wi. lucivorax* to *Pn. pyralis*), unscreened lampyrine species could also plausibly harbour their own endemic mollicute taxa. The microbiome of more firefly species should be characterized to determine whether they harbour new mollicute species, sisters to the ones that have already been identified.

## 5 Conclusion

Together, our results reveal that firefly microbiomes assemble under a composite regime of stochastic acquisition and deterministic retention, reflecting the diverse lifestyles of associated bacteria. First, transient microbes that are acquired through environmental sources and transit quickly through the gut (i.e., intragenerational transience; Ravenscraft and Coon 2026), appear common in fireflies. Stochastic assembly mechanisms dictate the fluctuating presence and abundance of transient microbes, likely including *Acinetobacter*, *Pseudomonas* and *Stenotrophomonas*. Second, several firefly‐associated mollicutes appear to be host‐adapted, specialized symbionts, combining signatures of deterministic retention through persistence across space and host development, and with evidence of stochastic acquisition, as they are not universally present within host species. While the transmission mode of mollicutes is still unknown, given their high prevalence and high abundance when present, they may play a role in the biology of firefly hosts (Data S1). Finally, parasitic microbes potentially involving *Enterococcus*, *Lactococcus*, *Rickettsia*, *Rickettsiella* and *Wolbachia* from the supergroup B, may proliferate to high abundances within the gut and internal tissues of fireflies. The distinction between parasites and specialized beneficial symbionts may be subtle in our study, as both can exhibit similar patterns of host association and establish through a combination of deterministic and stochastic processes. Future work integrating functional characterizations across a range of host and environmental conditions through combinations of targeted experimental antibiotic and fitness assays, genomics, and gene expression analyses will be crucial to elucidate the mechanisms underlying these contrasting dynamics and to test whether similar dual assembly regimes occur across other insect lineages. Fireflies thus emerge as a powerful system for probing how ecological and evolutionary forces jointly structure host‐microbe associations. By advancing our understanding of how deterministic and stochastic forces interact within an ecologically diverse beetle lineage, this research opens a new comparative framework for exploring the origins and maintenance of host‐microbe specificity.

## Author Contributions

B.B. designed the study, performed the dissections, analysed the data and wrote the initial manuscript. B.B. and S.E.L. collected specimens. B.B., S.R.N. and A.R. extracted DNA. B.B. and T.J.D. performed DNA barcoding. B.B. and A.R. processed the amplicon sequencing data. B.B., S.E.L. and A.R. contributed to review and editing of the manuscript. All authors read and approved the final version of the manuscript.

## Funding

This work was supported by the University of Texas at Arlington (RISE 100 Postdoctoral Fellowship to B.B., Research Innovation Grant from the College of Science to B.B. and A.R., and Startup Grant to A.R.) and Bucknell University (Startup Grant to S.E.L.).

## Conflicts of Interest

The authors declare no conflicts of interest.

## Supporting information


**Figure S1:** Host phylogenies. The first tree depicts a maximum likelihood phylogeny of the cytochrome c oxidase subunit 1 (COI) gene among firefly species. The tree was rooted with a click beetle *Melatonus depressus* COI sequence (EF546384.1). The tree contains, in addition to reference firefly COI sequences downloaded from NCBI, COI barcoding sequences from our study, shown here with labels in orange font and identifier starting with ‘FF’. Otherwise, branch and label colours correspond to the firefly clade colour coding used throughout the study. The first three collapsed clades shown as grey triangles correspond to, from top to bottom, the *Luciolinae* subfamily, the *Lamprohizinae* subfamily and the *Cheguevaria* genus. The second tree at the bottom focuses on the main firefly species and species groups represented in our study. It was built from a maximum likelihood 5‐locus phylogenetic analysis and contains more robust distances among firefly taxa. This tree was used to calculate phylogenetic distances among hosts (see Figure S7). The tree scales represent the number of nucleotide substitutions per site. All bootstrap values are shown on the tree nodes.
**Figure S2:** Overview of the approaches used to study firefly microbiomes. Following DNA extraction, samples were used in quantitative PCRs and Qubit DNA quantification to obtain bacterial titers and in Illumina amplicon sequencing of the 16S rRNA gene. The bioinformatic approach to process amplicon sequencing reads is given in rectangles with blue colours. Dark blue rectangles correspond to data processed through targeted taxonomic refinement (bacterial ‘taxon’ level). The main statistical analyses performed are shown on the right in pink rectangles.
**Figure S3:** Classification of strains from the *Mollicutes*. (A) Complete *Mollicutes* 16S rRNA phylogenetic tree. The tree was obtained from a maximum likelihood phylogenetic analysis on the 16S rRNA gene, rooted with a *Bacillus cereus* sequence (NR_074540.1), and contains 230 leaves of which 88 are 16S rRNA amplicon sequences from this study, four are amplicon sequences from another firefly microbiome study (Green et al. 2021), and four are amplicon sequences from another study on insect‐associated *Mollicutes* (Funaro et al. 2011). All the other leaves are full or near‐full length 16S rRNA gene sequences and were obtained either through NCBI searches to acquire reference sequence of mollicute species or through NCBI BLASTn runs to find the most similar sequences to our amplicon sequences. Red branches contain bacterial ASVs sampled from fireflies (from the present and previous studies). ASV labels in bold are among the most abundant in our dataset (SV identifier number lower than 151). Grey triangles correspond to collapsed monophyletic groups containing more than two non‐firefly associated strains. Genus and species names are from Gupta et al. (2019). Genera with dark grey fonts are commonly sampled from fireflies. The tree scale represents the number of nucleotide substitutions per site. All bootstrap values are shown on the tree. (B) Sequence dissimilarities among firefly‐associated ASVs from the *Mollicutes*. The Templeton, Crandall and Sing (TCS) network was produced via the PopART software using an alignment of all firefly‐derived *Mollicutes* ASV sequences with an SV identifier below 1500 (i.e., among the 1500 most abundant ASVs in our dataset). Hatch marks between nodes represent the number of mutations between two ASVs. Population genetics statistics are shown on the upper right of the figure. Identical ASVs (when considering only overlaps across sequences that were obtained from the two sequencing strategies) are shown on the lower left of the figure.
**Figure S4:** Phylogenetic trees of four bacterial groups important to insects including sequences from fireflies. All trees were obtained from a maximum likelihood phylogenetic analysis on the 16S rRNA gene except for the *Wolbachia* tree which was produced through a multi‐locus phylogeny with fragment‐insertion of 16S rRNA amplicon sequencing variants (ASVs) from our sequencing. For all trees, red branches contain bacterial ASVs sampled from fireflies (from the present study). ASV labels in bold are among the most abundant in our dataset (SV identifier number lower than 151). For the *Rickettsiella*, the *Rickettsiaceae* and the *Serratia* trees, all the leaves that are not labelled as ‘sv’ are full, near‐full length, or on a few occasions, fragments of the 16S rRNA gene sequences that were obtained either through NCBI searches to acquire reference sequence of bacterial species or through NCBI BLASTn runs to find the most similar sequences to our amplicon sequences. The *Rickettsiella* tree was rooted with a *Coxiella cheraxi* sequence (NR_116014.1) and contains 63 leaves of which 13 are 16S rRNA amplicon sequences from this study. The *Rickettsiaceae* tree was rooted with a *Candidatus* Endoecteinascidia sequence (DQ482575.1) and contains 163 leaves of which 19 are 16S rRNA amplicon sequences from this study, eight from the *Ca*. Hepatincola genus, four from the *Rickettsia* genus, two from the *Ca*. Hemipteriphilus genus, one from the *Ca*. Trichorickettsia genus, and two unidentified *Rickettsiales*. Note that *Ca*. Hepatincola is not always classified as from the *Rickettsiaceae* family but was through our classifications using the SILVA database. The *Serratia* tree was rooted with a *Yersinia enterocolitica* sequence (NR_041832.1) and contains 161 leaves of which 15 are 16S rRNA amplicon sequences from this study. For the *Wolbachia* tree, a backbone tree containing 160 leaves was first built from a maximum likelihood phylogenetic analysis based on a MAFFT alignment of seven genes (*coxA*, *fbpA*, *ftsZ*, *gatB*, *hcpA*, *recA* and the 16S rRNA gene) from *Wolbachia* and *Anaplasma* (outgroup) genomes (backbone tree available in our Data Repository). Our 16S rRNA amplicon sequences from abundant ASVs in our study were then inserted into the backbone tree which was rooted at the two *Anaplasma* leaves (one from multi‐locus another one from just the 16S rRNA gene (NR_118489.1)). The final tree contains 220 leaves of which 25 are 16S rRNA amplicon sequences from this study. The other leaves that were not part of the multi‐locus, backbone tree are full or near‐full length 16S rRNA gene sequences which were obtained through the same process as for the other trees. For all trees, grey triangles correspond to collapsed monophyletic groups containing more than two non‐firefly associated strains. Due to relatively short branch length, grey triangles my not always appear for collapsed clades, but the absence of labels should indicate that a clade has been collapsed (e.g., collapsed clade in the *Ca*. Hemipteriphilus‐related group from the *Rickettsiaceae* tree). The tree scales represent the number of nucleotide substitutions per site. All bootstrap values are shown on the *Rickettsiella* tree, only bootstrap values above 70 are shown for the *Rickettsia* and *Serratia* trees, and for the *Wolbachia* tree, no bootstrap values were available for fragment‐insertion trees (bootstrap support displayed on the backbone tree available in our Data Repository).
**Figure S5:** Normalized stochasticity ratio (NST) for 12 prevalent bacterial genera across firefly species. NST index values higher than 0.5 indicate a higher importance of stochastic forces governing bacterial community assembly, whereas values lower than 0.5 point to deterministic forces as the main drivers of community assembly. For each firefly species or species group the balls correspond to the index value and the error bars are 95% confidence intervals obtained from bootstrapping. Dark grey colour is for the complete, untouched microbiome and coloured colours correspond to microbiomes in which specific genera were removed. No significantly different NST values between the complete and modified microbiomes were found for either of these 12 bacterial genera. Two‐tailed Wilcoxon signed‐rank statistical tests were used to test the significance of these differences. Index values and statistical test results can be found in Table S6.
**Figure S6:** Normalized and non‐normalized absolute bacterial abundance across all firefly and related insect samples. Values are expressed as the log_10_ of the number of 16S rRNA gene copies in a sample, measured through qPCR, minus the number of 16S rRNA gene copy in the DNA extraction blank sample from the same batch, and then, only for normalized, divided by the total DNA concentration in the sample, which was measured via Qubit. Balls, diamonds and error bars represent the average, median and standard deviation, respectively. Raw qPCR data can be found in Table S3.
**Figure S7:** Alpha diversity indices calculated across firefly species, stages and sexes. The indices include the ASV richness, the Chao1index, the Shannon diversity index, the inverse Simpson diversity index, the Smith and Wilson's Evar evenness index, the Pielou evenness index, the Berger‐Parker dominance index, the dominance of core bacterial ASVs (those with detection level of 0.1% in over 20% of the samples) and the proportion of the least abundant bacterial ASVs (those below detection level of 0.2% determined separately in each sample) for each firefly species and across sexes and stages. Large size shapes and error bars represent the average and standard deviation, respectively, whereas the dots are the individual index values for each specimen. All alpha diversity metrics can be found in Table S7.
**Figure S8:** Microbiome dissimilarity increases with genetic and geographic distances. The graphs at the top show the positive correlation between pairwise phylogenetic distance across firefly species on the *x*‐axis and pairwise microbiome dissimilarity across firefly specimens on the *y*‐axis, calculated from relative rarefied abundance (graph on the left) and presence absence (graph on the right). Colours and shapes of data points represent the level of species comparisons: between individuals of the same species in down‐pointing grey triangles, between species from the *Photuris* genus in up‐pointing red triangles, between species from the *Photinus* genus in sea green diamonds, between species from the *Lampyrinae* subfamily in purple squares, and between species from different subfamilies in pink circles. The graphs at the bottom show the weak positive correlation between pairwise geographic distance across firefly collection sites on the *x*‐axis (expressed in kilometres) and pairwise microbiome dissimilarity across firefly specimens on the *y*‐axis, calculated from relative rarefied abundance (graph on the left) and presence absence (graph on the right).
**Figure S9:** Adult diet, not bioluminescence, influences the microbiome of fireflies. (A) Absolute bacterial abundance is not different between bioluminescent and non‐bioluminescent species. The *y*‐axis shows absolute bacterial abundance (expressed as in Figure 1C) and against firefly species grouped as bioluminescent at the adult stage or not on the *x*‐axis. All values were averaged per firefly species. Opaque balls and error bars correspond to predicted averages per group and 95% confidence intervals, respectively, obtained from ordinary least square (OLS) on the left graph and phylogenetic generalized least square (PGLS), which corrects for phylogenetic similarity biases due to the degree of genetic relatedness between firefly species, on the right graph. (B) Non‐metric multidimensional scaling (NMDS) analysis showing separation of the bacterial communities based on composition and relative abundance between bioluminescent and non‐bioluminescent fireflies. Four outgroup specimens were removed from these analyses. The NMDS is derived from Bray‐Curtis distances calculated on non‐rarefied, compositional relative abundance of taxon‐aggregated bacteria in firefly specimens. Permutational multivariate analyses of variance (PERMANOVA) were used for statistical testing of group similarities, computed on read depths rarefied at 30,000 reads. (C) Absolute bacterial abundance (expressed as in Figure 1C) vs. firefly species grouped as eaters (*Photinus corruscus*, *Photuris hebes* , *Pr. lucicrescens* and *Pr. versicolor* group) or non‐eaters (all other species). (D) Absolute bacterial abundance and bacterial richness vs. firefly species grouped based on their adult diet: nectar (*Pn. corruscus*), eating other fireflies (*Pr. hebes*, *Pr. lucicrescens* and *Pr. versicolor* group), or not eating (all other species). (E) NMDS analysis showing separation of the bacterial communities based on composition and relative abundance across firefly diets. In B and E, the NMDS stress was 0.262.
**Figure S10:** Relationships between firefly body size, absolute bacterial abundance, richness and diversity. The first four graphs (from left to right) show the correlation of absolute bacterial abundance (expressed as in Figure 1C) and bacterial richness vs. total firefly body length. The other graphs show how absolute bacterial abundance, bacterial richness and bacterial diversity correlate with one another in fireflies. All values were averaged per firefly species. Predicted correlation lines and 95% confidence interval shades were obtained from ordinary least square (OLS) and phylogenetic generalized least square (PGLS) models, which correct for phylogenetic similarity biases due to the degree of genetic relatedness between firefly species. Dot colours correspond to firefly species.
**Figure S11:** Bacterial composition in non‐lampyrid beetles. The bar graphs at the top represent non‐rarefied bacterial composition at the order level in five beetle groups, in addition to two firefly subfamilies. Abundance is the proportion of bacteria per specimen, obtained from compositional transformation of read depth. The composite heatmap at the bottom shows bacterial composition and relative abundance at the ‘taxon’ level, along with total absolute bacterial abundance per specimen (expressed as normalized bacterial titre, see Figure 1C) and prevalence of single bacteria across these beetles, calculated without a filter and with a filter at 100 raw reads (i.e., bacterial taxa with an abundance lower than 100 reads in a specimen were considered absent in the calculation of prevalence). Note that the two tiger beetles (*Cicindelidae*) were already dead at the time of collection.
**Figure S12:** Absolute abundance of bacterial taxa across firefly species. In each bargraph the absolute abundance of the top 10 most abundant bacterial taxa per firefly specimen (*x*‐axis) is represented on the *y*‐axis. Absolute abundance was calculated as the relative bacterial taxon abundance (rarefied read depth proportion) was multiplied by total bacterial titre in a specimen (log_10_ of 16S rRNA gene copy number divided by total DNA concentration in specimens).
**Figure S13:** Abundance of 33 bacterial taxa across firefly species and other elateroid beetles. These bacteria are among the most abundant and prevalent in fireflies from our dataset. For each of them, rarefied read depth (at 30,000 reads) is shown on the *x*‐axis. Firefly species are on the *y*‐axis. Balls and error bars represent the per species average and standard deviation, respectively. The asterisks indicate when a bacterium was considered as a characteristic bacterial taxon in a given firefly species. To account for prevalence, abundance and other random effects, we classified bacterial taxa as characteristic of a firefly species if (1) *MaAsLin2* multiple generalized linear mixed models indicated that they were significantly more abundant in a given species than in at least another one with a *p* < 0.001, and (2) they were found with an abundance of over 15,000 rarefied read depth in at least two specimens from the focal species. A summary of the *MaAsLin2* results is available in Table S8.
**Figure S14:** Composition of bacterial taxa across firefly species after excluding mollicutes and putative endosymbionts. Only firefly species with more than three specimens in our dataset post rarefying are shown. The relative rarefied abundance of the most abundant bacteria is shown as different colours whereas the abundance of all other bacteria is in light grey. Bacteria classified as from the *Candidatus* Hepatincola, *Mollicutes*, *Rickettsia*, *Rickettsiella*, *Serratia* and *Wolbachia* were excluded from the dataset before generating these graphs. Bacterial colours here approximately match those in Figure 6 and Figure S17.
**Figure S15:** Positive, negative and random co‐occurrence among bacterial taxa associated with fireflies. The heatmaps were generated with the *CooccurrenceAffinity* R package. Red colours correspond to positive co‐occurrence, blue colour is for negative co‐occurrence, and white for random co‐occurrence. The darkest colours, very high or very low alpha MLE values (see Mainali et al., 2022) are for bacterial taxa that almost always or never co‐occur, respectively. Dark borders indicate a positive or negative co‐occurrence pattern supported by inferences from the *cooccur* R package. Asterisks correspond to different levels of statistical significance in the positive or negative co‐occurrence obtained from *CooccurrenceAffinity*, with ‘·’: *p* < 0.1, ‘*’: *p* < 0.05, ‘**’: *p* < 0.01, ‘***’: *p* < 0.001.
**Figure S16:** Microbiome divergence across fireflies from different regions. Non‐metric multidimensional scaling (NMDS) analyses showing the separation of the bacterial communities based on composition and relative abundance across regions for four firefly species and two species groups. All plots are derived from Bray‐Curtis distances calculated on non‐rarefied, compositional relative abundance of taxon‐aggregated bacteria in firefly specimens. The NMDS stress values were 0.130 for *Photinus corruscus*, 0.133 for *Pn. marginellus* group, 0.202 for *Pn. pyralis*, 0.148 for *Pn. scintillans*, 0.244 for *Photuris hebes* and 0.245 for *Pr. versicolor* group. Results from *MaAsLin2* multiple generalized linear mixed models, inferring bacterial differential abundance, can be found in Table S9 and results from PERMANOVAs and pairwise PERMANOVAs comparing regional microbiomes per species can be found in Table S12.
**Figure S17:** Most differentially abundant bacteria across the years in a firefly species collected in a single location. *Photinus pyralis* fireflies were collected in Philadelphia Fairmont Park in the month of July 2021, 2022 and 2023. Bacterial colours here approximately match those in Figure 6 and Figure S14.
**Figure S18:** Significantly differentially abundant bacterial taxa across habitat types in unidentified species from the *Photuris versicolor* group. These bacteria are among those with the most significantly different abundance across habitat in this firefly species group, as assessed through *MaAsLin2* multiple generalized linear mixed models (GLMMs). For each of these bacteria, a univariate GLMM was run to test whether its rarefied abundance differs across these fireflies' habitats, when controlling for sex type, region, day of year and year of capture. The most consistent results were observed for *Rickettsiella costelytrae*. Bacterial taxon abundance is expressed as the log_10_ of the bacterial read depth per firefly specimen in our rarefied amplicon sequencing dataset. Results from *MaAsLin2* runs can be found in Table S10.
**Figure S19:** Male *Photinus* microbiomes are more even than females'. A univariate generalized linear mixed (GLMM) model was used to test whether the index of Evar evenness is different between females and males in *Photinus* and *Photuris* fireflies, when controlling for sampling location, day of year, year of capture and sequencing type. Significant differences are shown with lowercase English letters. Predicted data with 95% confidence intervals were obtained from the GLMMs. Results from the GLMM can be found in Table S12.
**Figure S20:** Bacterial absolute abundance and diversity in adults, larvae and pupae. This figure is similar to Figure 7F, except that the juvenile category was broken down into larvae and pupae. Univariate generalized linear mixed models (GLMMs) were used to test whether bacterial titre, bacterial richness and the Shannon diversity index are different between adults, larvae and pupae, using firefly genus as a covariate and controlling for region, day of year, and year of capture. Bacterial titre is expressed as in Figure 1C. Predicted data with 95% confidence intervals were obtained from the GLMMs. Results from the GLMM can be found in Table S12.
**Figure S21:** Bacterial composition in larvae and adults of three firefly species. The composite heatmaps show bacterial composition and relative abundance for the *Photinus*, *Photuris* and *Pyractomena* genera. Relative abundance values were obtained from compositional transformation of non‐rarefied read depth, at the ‘taxon’ level, along with total absolute bacterial abundance per specimen (expressed as normalized bacterial titre, see Figure 1C) and prevalence of single bacteria across these firefly specimens, calculated without a filter and with a filter at 500 raw reads (i.e., bacterial taxa with an abundance lower than 500 reads in a specimen were considered absent in the calculation of prevalence).
**Figure S22:** Abundance of *Williamsoniiplasma somnilux* in *Pyractomena* fireflies is higher in larvae than in adults and pupae. *Wi. somnilux* abundance is expressed as the log_10_ of *Wi. somnilux* read depth per firefly specimen in our rarefied amplicon sequencing dataset in the first panel and as the relative compositional abundance of *Wi. somnilux* in our non‐rarefied amplicon sequencing dataset in the second panel (more data points). A univariate generalized linear mixed (GLMM) model was used to test whether *Wi. somnilux* abundance is influenced by developmental stage in *Pyractomena* fireflies, when controlling for species, region, day of year and year of collection. Predicted mean with 95% confidence intervals were obtained from the GLMMs. Results from the GLMM can be found in Table S12.
**Figure S23:** Microbiome divergence across firefly tissues. Non‐metric multidimensional scaling (NMDS) analyses showing the separation of the bacterial communities based on composition and relative abundance across tissues for three firefly species and species group. All plots are derived from Bray‐Curtis distances calculated on non‐rarefied, compositional relative abundance (i.e., ‘proportional abundance’) of taxon‐aggregated bacteria in firefly specimens. One outlier was removed from the first and second graphs. The NMDS stress was 0.218 for across species, 0.179 for *Photinus pyralis* , 0.150 for *Pn. corruscus* and 0.175 for *Photuris versicolor* group. Results from *MaAsLin2* multiple generalized linear mixed models, inferring bacterial differential abundance, can be found in Table S11 and results from PERMANOVAs and pairwise PERMANOVAs comparing tissue microbiomes per species can be found in Table S12.
**Figure S24:** Bacterial composition in different tissues of *Photinus corruscus* fireflies. The composite heatmap at the top shows bacterial composition and relative abundance, obtained from compositional transformation of non‐rarefied read depth, at the ‘taxon’ level, along with total absolute bacterial abundance per specimen (expressed as normalized bacterial titre, see Figure 1C) and prevalence of single bacteria across specimen tissues, calculated without a filter and with a filter at 500 raw reads (i.e., bacterial taxa with an abundance lower than 500 reads in a specimen were considered absent in the calculation of prevalence). Photographs of dissections with anatomical annotations are shown at the bottom.
**Figure S25:** Bacterial composition in different tissues of *Photinus pyralis* fireflies. The composite heatmap at the top shows bacterial composition and relative abundance, obtained from compositional transformation of non‐rarefied read depth, at the ‘taxon’ level, along with total absolute bacterial abundance per specimen (expressed as normalized bacterial titre, see Figure 1C) and prevalence of single bacteria across specimen tissues, calculated without a filter and with a filter at 500 raw reads (i.e., bacterial taxa with an abundance lower than 500 reads in a specimen were considered absent in the calculation of prevalence). Photographs of dissected organs with anatomical annotations are shown at the bottom.
**Figure S26:** Bacterial composition in different tissues of unidentified species from the *Photuris versicolor* group. The composite heatmap at the top shows bacterial composition and relative abundance, obtained from compositional transformation of non‐rarefied read depth, at the ‘taxon’ level, along with total absolute bacterial abundance per specimen (expressed as normalized bacterial titre, see Figure 1C) and prevalence of single bacteria across specimen tissues, calculated without a filter and with a filter at 500 raw reads (i.e., bacterial taxa with an abundance lower than 500 reads in a specimen were considered absent in the calculation of prevalence). Photographs of mouth parts and dissections with anatomical annotations are shown at the bottom.

**Table S1:** Firefly sample and specimen information, including total bacterial titers. For Sex type, L: larva, F: female, M: male, P: pupa. Collection habitat type is given as a negative urbanization intensity gradient, with (1) City or city park a few metres away from a busy road; (2) Private lawn that is regularly mowed, near a house and road; (3) Park that has limited light pollution, dense vegetation, but signs of human activity (litter), and is a few hundred metres away from a busy road; (4) Undisturbed meadow with low tree density and vast grassland; (5) Undisturbed area made of a combination of meadows, woodlands and/or streams and ponds; (6) Undisturbed land with low to high forest density. Temperature and rainy day was obtained from https://www.wunderground.com which collects meteorological data from nearest weather stations. Pictures of photographed specimens are available in data repository. Bacterial absolute abundance is given at the end, with raw bacterial titre, DNA concentration and normalized abundance.
**Table S2:** Firefly dissection information. All specimens were surface‐sterilized with 10% bleach and 99% ethanol, and rinsed in ultra pure, sterile PCR water. Most specimens were photographed before dissection. Photographs are available in Supporting Information and as data repository. Dissection blanks, highlighted in yellow here, consisted of a firefly leg left in a water droplet on the side of the dissection area. Dissection blank was usually changed every one or two samples.
**Table S3:** Raw qPCR plate data. Each DNA sample was run in two or three technical replicates (i.e., plates). An average of the replicate was used as the estimate of bacterial titre. Standards were diluted PCR products containing a known quantity of 16S rRNA genes. This PCR product was obtained from the DNA of a *Caballeronia* symbiont (Lep1A1) isolated from a *Leptoglossus zonatus* individual. The number of 16S rRNA genes in the PCR product was calculated from DNA concentration in this product, measured with Qubit. Eight ten‐fold standard dilution samples, from 10^1^ to 10^8^, and 4 μL of template DNA (for volume in each reaction = 20 μL) were used in each plate. The qPCR efficiency index was calculated as (10^((−1)^/(slope of standard curve)))‐1 in each plate. Positive controls included DNA extracted from late instar and adult *L. phyllopus* . To correct for potential contamination, raw titre of all samples in a plate was first subtracted by the titre obtained in DNA extraction blank samples. For dissected samples, titre obtained was then subtracted by titre obtained in dissection blank samples. Extraction and dissection blank samples are highlighted in yellow here. Raw qPCR data are available in data repository.
**Table S4:** NextSeq sequencing information and quality filtering. Quality filtering was run with the *dada2* R package.
**Table S5:** List of potential bacterial contaminants. Bacteria in this list passed the decontamination filtering of potential contaminant ASVs from the *decontam* R package but had their relative abundance negatively correlating with total bacterial abundance in firefly species, potentially indicative of contamination. Results were obtained from *MaAsLin2* multiple GLMM runs with negative binomial distribution and controlling for day of year, region and sex type. This test was only computed on species and genera with a good number of specimens: *Pn. corruscus*, *Pn. pyralis*, *Pn. marginellus* group, *Pn. scintillans*, *Photuris* sp. and *Pyractomena* sp. Column D: N gives the total number of samples that were originally in the dataset. Next column: N.not.0 shows the number of samples that do not have a zero relative abundance for a bacterial taxon. Parameters used for within‐species comparison: distribution method = negative binomial, transform = no, normalization = none, minimum prevalence threshold = 0.01, maximum significance threshold = 0.05, formula for random effects = expr ~ (1 | yday) + (1 | Region) + (1 | Sex), with ‘yday’ being day of year. The number of bacterial taxa filtered before run is shown in column B: Total filtered features. References for studies cited in the Note column (J) can be found below the table. A list of contaminant removed using the decontam R package is available in our data repository.
**Table S6:** Normalized stochasticity ratio (NST) and comparisons with and without specific bacterial groups. The NST index was obtained and tested thanks to the *NST* R package. Note that the comparisons to the ‘Only Mollicutes’ group (relative abundance of all bacteria removed from microbiome dataset of firefly species except for that of mollicute taxa) is biassed due to a much lower number of non‐zero values in this group compared to complete microbiome or microbiomes with only mollicutes, endosymbionts or both removed, and is not represented in Figure 2. The *W* value in columns F and H was obtained from two‐tailed Wilcoxon signed‐rank statistical tests. The last two columns show statistics for tests performed on data without outliers, which were determined as values higher or lower than 1.5‐fold of the interquartile range. Bold font correspond to significant results, with *p* < 0.05.
**Table S7:** Alpha diversity metrics calculated on whole body firefly microbiomes. Abundance was rarefied at 30,000 reads. Indices of alpha diversity were calculated using the *microbiome* R package. The criteria to compute the core abundance index (i.e., relative proportion of the core species) were manually modified, with detection level of 0.1% in over 20% of the samples.
**Table S8:** Significantly differentially abundant bacterial taxa across firefly subfamilies, genera and species and between fireflies and non‐firefly beetles. Multiple GLMMs were run with the *MaAsLin2* R package. Only bacteria with a negative coefficient (relatively higher abundance in reference firefly taxon) and a *Q*‐value (i.e., FDR‐adjusted *p*‐values) < 0.001 are reported here, except for the comparison firefly vs. non‐firefly where the *Q*‐value threshold was 0.01 instead. The last column indicates whether the bacterial taxon is of high abundance in the firefly species given in the Reference column (C). Only firefly species level comparisons were shown in that column and only the 33 most abundant bacterial taxa shown in Figure S13 are considered here. Examples of parameters used, here for subfamily‐level comparison: distribution method = negative binomial, transform = no, normalization = none, minimum prevalence threshold = 0.01, maximum significance threshold = 0.001, formula for random effects = expr ~ (1 | Date_capture) + (1 | Address) + (1 | Sex). The same parameters were used in the other tests, except in our genus‐ and species‐level comparisons, where for genera and species with low sample size, including *Lucidota*, *Lu. atra*, *Photinus consanguineus* group, *Photuris lucicrescens* , *Pyractomena* and *Py. borealis*, the minimum prevalence threshold was reduced to 0.001 to keep all bacterial taxa in the analysis. The number of bacterial taxa filtered is shown in column E: Total filtered features. For species‐level comparisons, the random factor Date of capture was replaced by day of year. Column H: N gives the total number of samples that were originally in the dataset. Next column: N.not.0 shows the number of samples that do not have a zero relative abundance for a bacterial taxon.
**Table S9:** Significantly differentially abundant bacterial taxa across regions within different firefly species. Multiple GLMMs were run with the *MaAsLin2* R package. Only bacteria with a *Q*‐value (i.e., FDR‐adjusted *p*‐values) < 0.05 are reported here. Parameters used for within‐species comparison: distribution method = negative binomial, transform = no, normalization = none, minimum prevalence threshold = 0.01, maximum significance threshold = 0.05, formula for random effects = expr ~ (1 | Year_capture) + (1 | yday) + (1 | Sex), with ‘yday’ being day of year. For comparisons where the reference region had low sample size, the minimum prevalence threshold was reduced to 0.001 to keep a higher number of bacterial taxa in the analysis. The number of bacterial taxa filtered is shown in column D: Total filtered features. Column F: N gives the total number of samples that were originally in the dataset. Next column: N.not.0 shows the number of samples that do not have a zero relative abundance for a bacterial taxon.
**Table S10:** Significantly differentially abundant bacterial taxa across different factors. Multiple GLMMs were run with the *MaAsLin2* R package. Only bacteria with a *Q*‐value (i.e., FDR‐adjusted *p*‐values) < 0.05 are reported here, except for the diet comparison where the *Q*‐value of 0.0001 threshold was used. Parameters used for habitat type comparison: distribution method = negative binomial, transform = no, normalization = none, minimum prevalence threshold = 0.01, maximum significance threshold = 0.05, formula for random effects = expr ~ (1 | Year_capture) + (1 | yday) + (1 | Sex) + (1 | Region), with ‘yday’ being day of year. Similar parameters were used for stage comparisons with the following exceptions: minimum prevalence threshold = 0.001 and formula for random effects = expr ~ (1 | Date of capture) + (1 | Address) + (1 | Species). For the sex and stage comparisons in Pyractomena, the term species.sex was used as a covariate fixed effect while sex was the main fixed effect. For these comparisons, minimum prevalence threshold = 0.001 and formula for random effects = expr ~ (1 | Date of capture) + (1 | Address). Finally, for bioluminescent and diet comparisons, *Q*‐value shown is < 0.0001 (no significant results for bioluminescence, always > 0.5), the only fixed effect in the models was either bioluminescence or diet, and the formula for random effects = expr ~ (1 | Year_capture) + (1 | yday) + (1 | Region) + (1 | Species). The number of bacterial taxa filtered is shown in column E: Total filtered features. Column H: N gives the total number of samples that were originally in the dataset. Next column: N.not.0 shows the number of samples that do not have a zero relative abundance for a bacterial taxon.
**Table S11:** Significantly differentially abundant bacterial taxa across tissues. Multiple GLMMs were run with non‐rarefied data using the *MaAsLin2* R package. Only bacteria with a *Q*‐value (i.e., FDR‐adjusted *p*‐values) < 0.05 are reported here. Parameters used for within‐species tissue comparisons: distribution method = negative binomial, transform = no, normalization = cumulative sum scaling (CSS), minimum prevalence threshold = 0.01, maximum significance threshold = 0.05, formula for random effects = expr ~ (1 | Date of capture) + (1 | Address) + (1 | Sex). The formula for the tissue:sex interaction comparison in *Photuris versicolor* group only includes date of capture and address, but all parameters are the same as in the previous tests. For these interactions tests, only results with Full gut as the reference are reported here. The number of bacterial taxa filtered is shown in column D: Total filtered features. Column G: N gives the total number of samples that were originally in the dataset. Next column: N.not.0 shows the number of samples that do not have a zero relative abundance for a bacterial taxon.
**Table S12:** Model description and statistical results for linear mixed models, PERMANOVAs, pairwise PERMANOVAs, Mantel tests and PGLSs/OLSs used to test the difference in bacterial abundance and diversity across multiple factors and correlations between microbiome variables and other factors. Column A: Specimens indicates which host taxonomic groups were used in the analysis. Column B: Response corresponds to the dependent variable being tested. Column C: Main fixed effect shows which independent variable(s) is/are tested and how (e.g., polynomial, interaction). Column D: Type of analysis shows the type of statistical test used and what is displayed in the next column. Column E: Output shows the raw output from R, with, in bold font, the relevant significant results at *p* < 0.05.

**Data S1:** mec70473‐sup‐0003‐DataS1.

## Data Availability

All 16S rRNA gene amplicon sequencing data have been deposited in the NCBI Short Read Archive under BioProject PRJNA1237523. The COI DNA barcoding sequences produced from Sanger sequencing are available from the NCBI GenBank database with accession numbers PV844642–PV844700. Supporting Information can be found in our data repository at: https://doi.org/10.6084/m9.figshare.32599836.
