# Supplementary material for: Species‐Specific Bacterial Associations Emerge From Stochastically Assembled Microbiomes in Northeastern American Fireflies
Source: Mol Ecol. 2026 Jul 18;35(14):e70473. doi: 10.1111/mec.70473 (PMC13380324; doi:10.1111/mec.70473)
Supplement: Supplementary file 1 — Figure S1: Host phylogenies. The first tree depicts a maximum likelihood phylogeny of the cytochrome c oxidase subunit 1 (COI) gene among firefly species. The tree was rooted with a click beetle Melatonus depressus COI sequence (EF546384.1). The tree contains, in addition to reference firefly COI sequences downloaded from NCBI, COI barcoding sequences from our study, shown here with labels in orange font and identifier starting with ‘FF’. Otherwise, branch and label colours correspond to the firefly clade colour coding used throughout the study. The first three collapsed clades shown as grey triangles correspond to, from top to bottom, the Luciolinae subfamily, the Lamprohizinae subfamily and the Cheguevaria genus. The second tree at the bottom focuses on the main firefly species and species groups represented in our study. It was built from a maximum likelihood 5‐locus phylogenetic analysis and contains more robust distances among firefly taxa. This tree was used to calculate phylogenetic distances among hosts (see Figure S7). The tree scales represent the number of nucleotide substitutions per site. All bootstrap values are shown on the tree nodes. Figure S2: Overview of the approaches used to study firefly microbiomes. Following DNA extraction, samples were used in quantitative PCRs and Qubit DNA quantification to obtain bacterial titers and in Illumina amplicon sequencing of the 16S rRNA gene. The bioinformatic approach to process amplicon sequencing reads is given in rectangles with blue colours. Dark blue rectangles correspond to data processed through targeted taxonomic refinement (bacterial ‘taxon’ level). The main statistical analyses performed are shown on the right in pink rectangles. Figure S3: Classification of strains from the Mollicutes. (A) Complete Mollicutes 16S rRNA phylogenetic tree. The tree was obtained from a maximum likelihood phylogenetic analysis on the 16S rRNA gene, rooted with a Bacillus cereus sequence (NR_074540.1), and contains 230 le [file MEC-35-e70473-s002.docx]

**Species-specific bacterial associations emerge from stochastically assembled microbiomes in Northeastern American fireflies**

Benoît Béchade*^1^, Sarah E. Lower^2^, Sierra R. Nichols^1^, Tanner J. Dabbert^1^, and Alison Ravenscraft^1^

^1^: Department of Biology, University of Texas at Arlington, 501 S. Nedderman Dr., Arlington, TX 76019, USA

^2^: Department of Biology, Bucknell University, One Dent Drive, Lewisburg, PA 17837, USA.

*: Corresponding author; Email: [ben.bechade@gmail.com](mailto:ben.bechade@gmail.com).

**Supplementary Figures S1–S26**

**
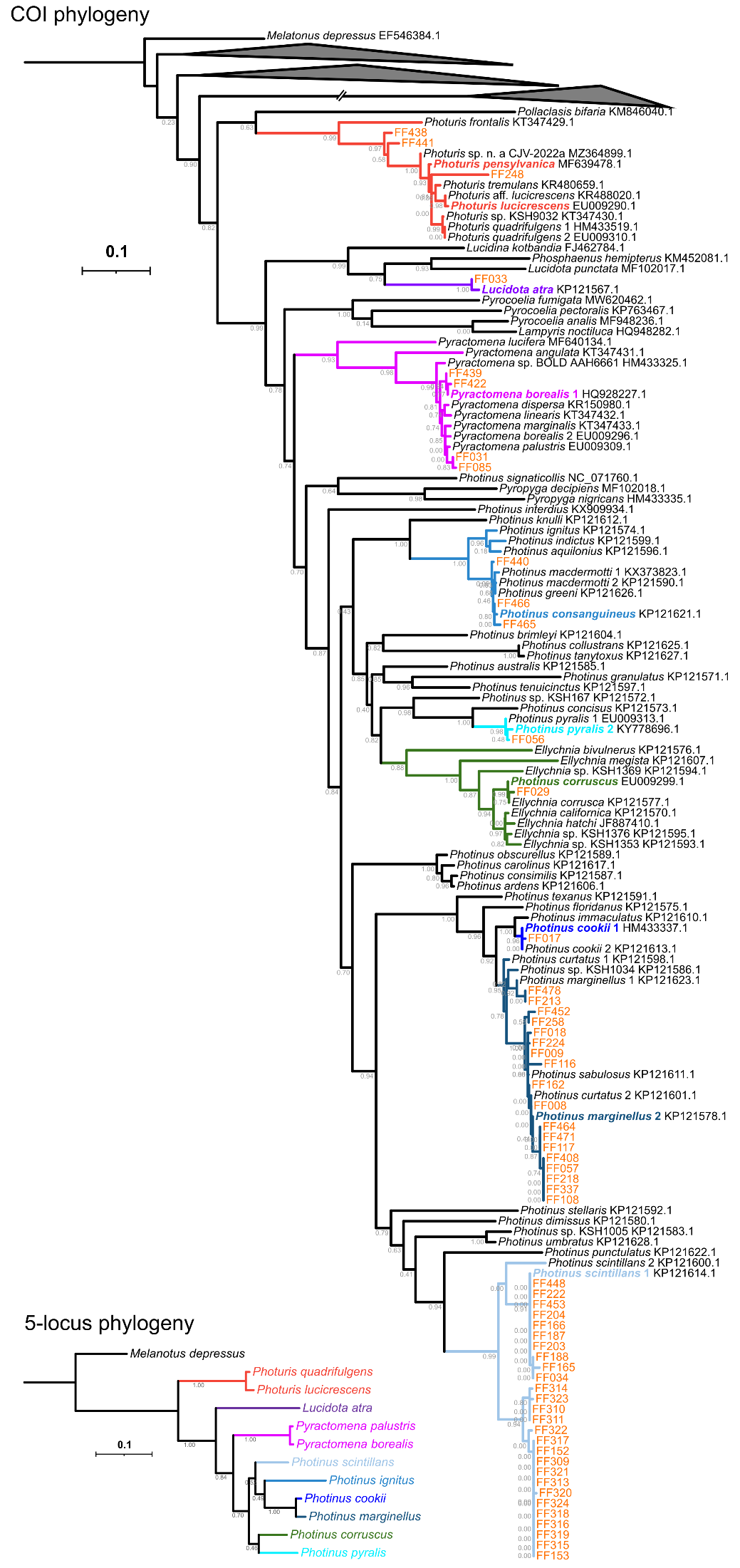
**

**Figure S1: Host phylogenies**. The first tree depicts a maximum likelihood phylogeny of the cytochrome c oxidase subunit 1 (COI) gene among firefly species. The tree was rooted with a click beetle *Melatonus depressus* COI sequence (EF546384.1). The tree contains, in addition to reference firefly COI sequences downloaded from NCBI, COI barcoding sequences from our study, shown here with labels in orange font and identifier starting with “FF”. Otherwise, branch and label colors correspond to the firefly clade color coding used throughout the study. The first three collapsed clades shown as gray triangles correspond to, from top to bottom, the *Luciolinae* subfamily, the *Lamprohizinae* subfamily, and the *Cheguevaria* genus. The second tree at the bottom focuses on the main firefly species and species groups represented in our study. It was built from a maximum likelihood 5-locus phylogenetic analysis and contains more robust distances among firefly taxa. This tree was used to calculate phylogenetic distances among hosts (see Figure S7). The tree scales represent the number of nucleotide substitutions per site. All bootstrap values are shown on the tree nodes.

**
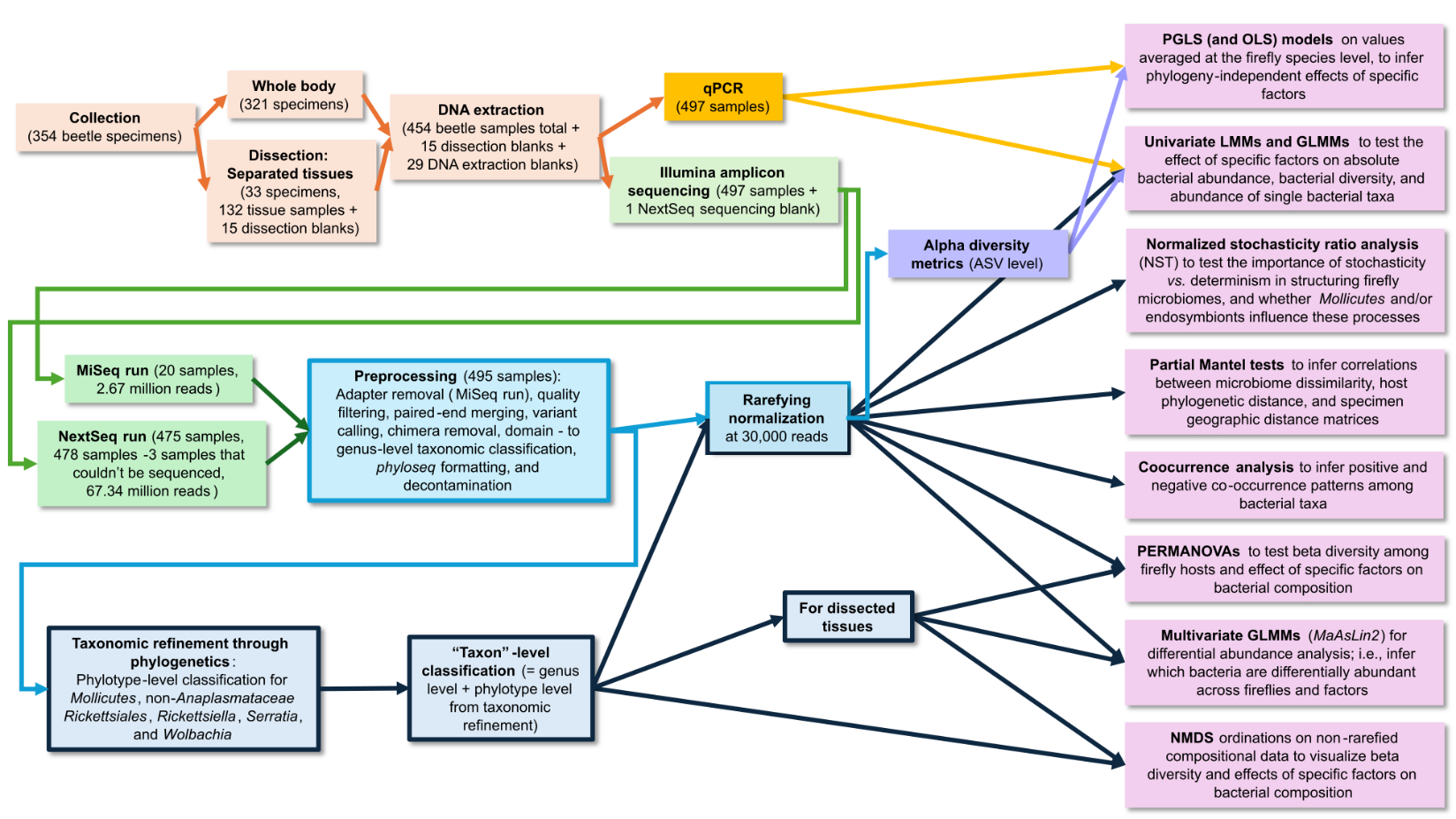
**

**Figure S2: Overview of the approaches used to study firefly microbiomes**. Following DNA extraction, samples were used in quantitative PCRs and Qubit DNA quantification to obtain bacterial titers and in Illumina amplicon sequencing of the 16S rRNA gene. The bioinformatic approach to process amplicon sequencing reads is given in rectangles with blue colors. Dark blue rectangles correspond to data processed through targeted taxonomic refinement (bacterial “taxon” level). The main statistical analyses performed are shown on the right in pink rectangles.

**
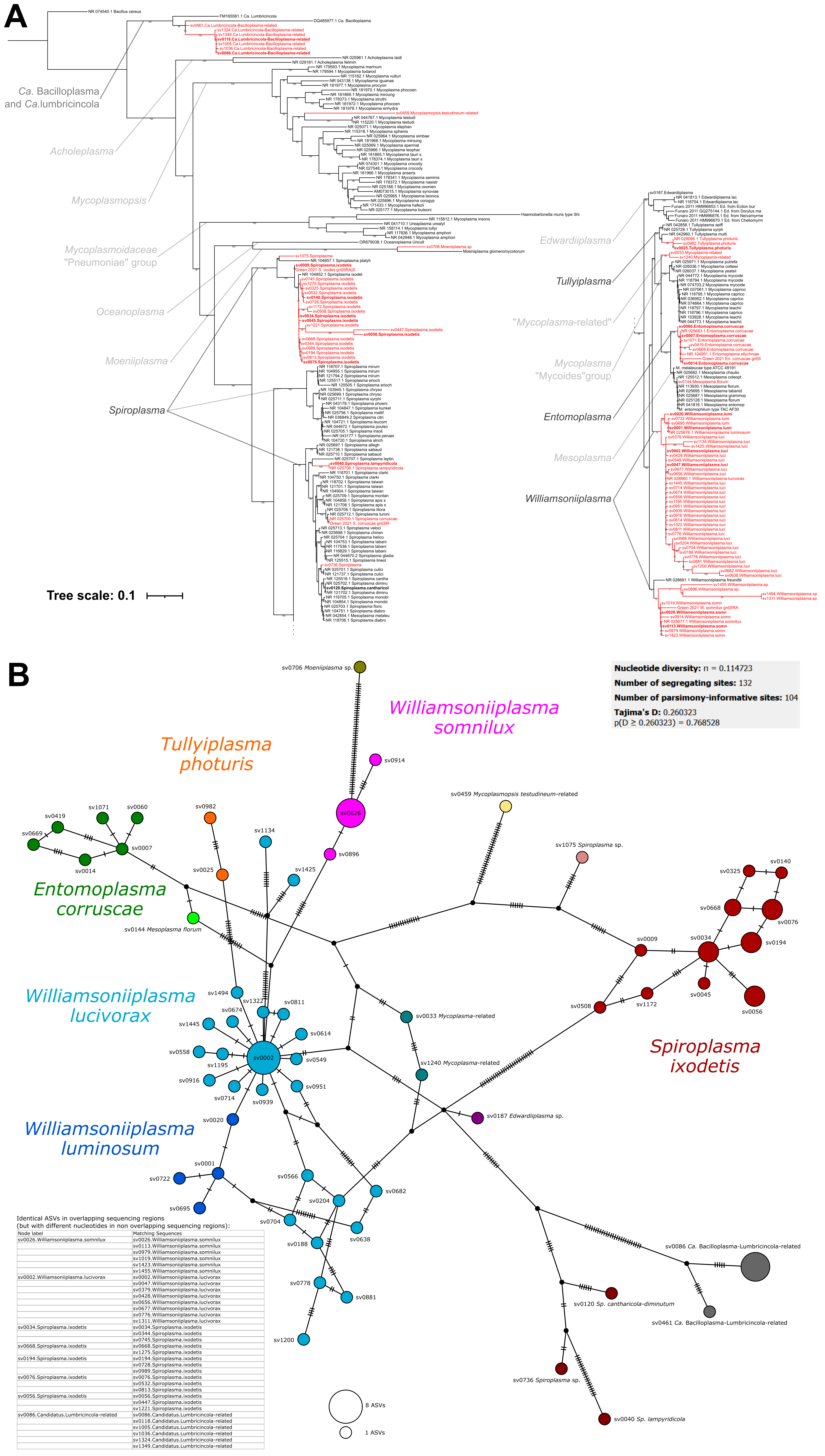
**

**Figure S3: Classification of strains from the *Mollicutes***. **A**. Complete *Mollicutes* 16S rRNA phylogenetic tree. The tree was obtained from a maximum likelihood phylogenetic analysis on the 16S rRNA gene, rooted with a *Bacillus cereus* sequence (NR_074540.1), and contains 230 leaves of which 88 are 16S rRNA amplicon sequences from this study, four are amplicon sequences from another firefly microbiome study (Green et al. 2021), and four are amplicon sequences from another study on insect-associated *Mollicutes* (Funaro et al. 2011). All the other leaves are full or near-full length 16S rRNA gene sequences and were obtained either through NCBI searches to acquire reference sequence of mollicute species or through NCBI BLASTn runs to find the most similar sequences to our amplicon sequences. Red branches contain bacterial ASVs sampled from fireflies (from the present and previous studies). ASV labels in bold are among the most abundant in our dataset (SV identifier number lower than 151). Gray triangles correspond to collapsed monophyletic groups containing more than two non-firefly associated strains. Genus and species names are from Gupta et al. (2019). Genera with dark gray fonts are commonly sampled from fireflies. The tree scale represents the number of nucleotide substitutions per site. All bootstrap values are shown on the tree. **B**. Sequence dissimilarities among firefly-associated ASVs from the *Mollicutes*. The Templeton, Crandall, and Sing (TCS) network was produced via the PopART software using an alignment of all firefly-derived *Mollicutes* ASV sequences with an SV identifier below 1,500 (i.e., among the 1,500 most abundant ASVs in our dataset). Hatch marks between nodes represent the number of mutations between two ASVs. Population genetics statistics are shown on the upper right of the figure. Identical ASVs (when considering only overlaps across sequences that were obtained from the two sequencing strategies) are shown on the lower left of the figure.

**
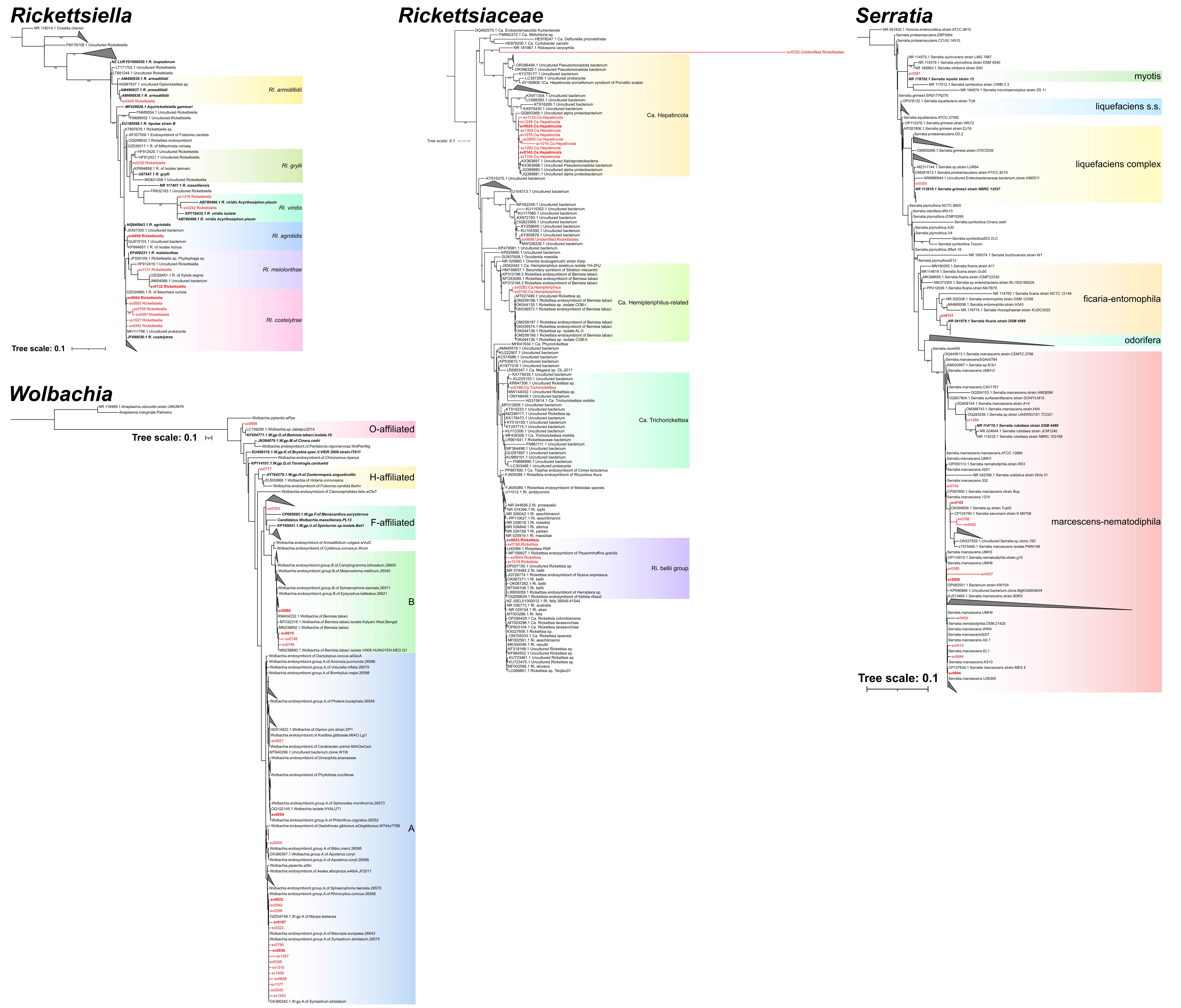
**

**Figure S4: Phylogenetic trees of four bacterial groups important to insects including sequences from fireflies**. All trees were obtained from a maximum likelihood phylogenetic analysis on the 16S rRNA gene except for the *Wolbachia* tree which was produced through a multi-locus phylogeny with fragment-insertion of 16S rRNA amplicon sequencing variants (ASVs) from our sequencing. For all trees, red branches contain bacterial ASVs sampled from fireflies (from the present study). ASV labels in bold are among the most abundant in our dataset (SV identifier number lower than 151). For the *Rickettsiella*, the *Rickettsiaceae*, and the *Serratia* trees, all the leaves that are not labeled as “sv” are full, near-full length, or on a few occasions, fragments of the 16S rRNA gene sequences that were obtained either through NCBI searches to acquire reference sequence of bacterial species or through NCBI BLASTn runs to find the most similar sequences to our amplicon sequences. The *Rickettsiella* tree was rooted with a *Coxiella cheraxi* sequence (NR_116014.1) and contains 63 leaves of which 13 are 16S rRNA amplicon sequences from this study. The *Rickettsiaceae* tree was rooted with a *Candidatus* Endoecteinascidia sequence (DQ482575.1) and contains 163 leaves of which 19 are 16S rRNA amplicon sequences from this study, eight from the *Ca.* Hepatincola genus, four from the *Rickettsia* genus, two from the *Ca.* Hemipteriphilus genus, one from the *Ca.* Trichorickettsia genus, and two unidentified *Rickettsiales*. Note that *Ca.* Hepatincola is not always classified as from the *Rickettsiaceae* family but was through our classifications using the SILVA database. The *Serratia* tree was rooted with a *Yersinia enterocolitica* sequence (NR_041832.1) and contains 161 leaves of which 15 are 16S rRNA amplicon sequences from this study. For the *Wolbachia* tree, a backbone tree containing 160 leaves was first built from a maximum likelihood phylogenetic analysis based on a MAFFT alignment of seven genes (*coxA*, *fbpA*, *ftsZ*, *gatB*, *hcpA*, *recA*, and the 16S rRNA gene) from *Wolbachia* and *Anaplasma* (outgroup) genomes (backbone tree available in our Data Repository). Our 16S rRNA amplicon sequences from abundant ASVs in our study were then inserted into the backbone tree which was rooted at the two *Anaplasma* leaves (one from multi-locus another one from just the 16S rRNA gene (NR_118489.1)). The final tree contains 220 leaves of which 25 are 16S rRNA amplicon sequences from this study. The other leaves that were not part of the multi-locus, backbone tree are full or near-full length 16S rRNA gene sequences which were obtained through the same process as for the other trees. For all trees, gray triangles correspond to collapsed monophyletic groups containing more than two non-firefly associated strains. Due to relatively short branch length, gray triangles my not always appear for collapsed clades, but the absence of labels should indicate that a clade has been collapsed (e.g. collapsed clade in the *Ca.* Hemipteriphilus-related group from the *Rickettsiaceae* tree). The tree scales represent the number of nucleotide substitutions per site. All bootstrap values are shown on the *Rickettsiella* tree, only bootstrap values above 70 are shown for the *Rickettsia* and *Serratia* trees, and for the *Wolbachia* tree, no bootstrap values were available for fragment-insertion trees (bootstrap support displayed on the backbone tree available in our Data Repository).

**
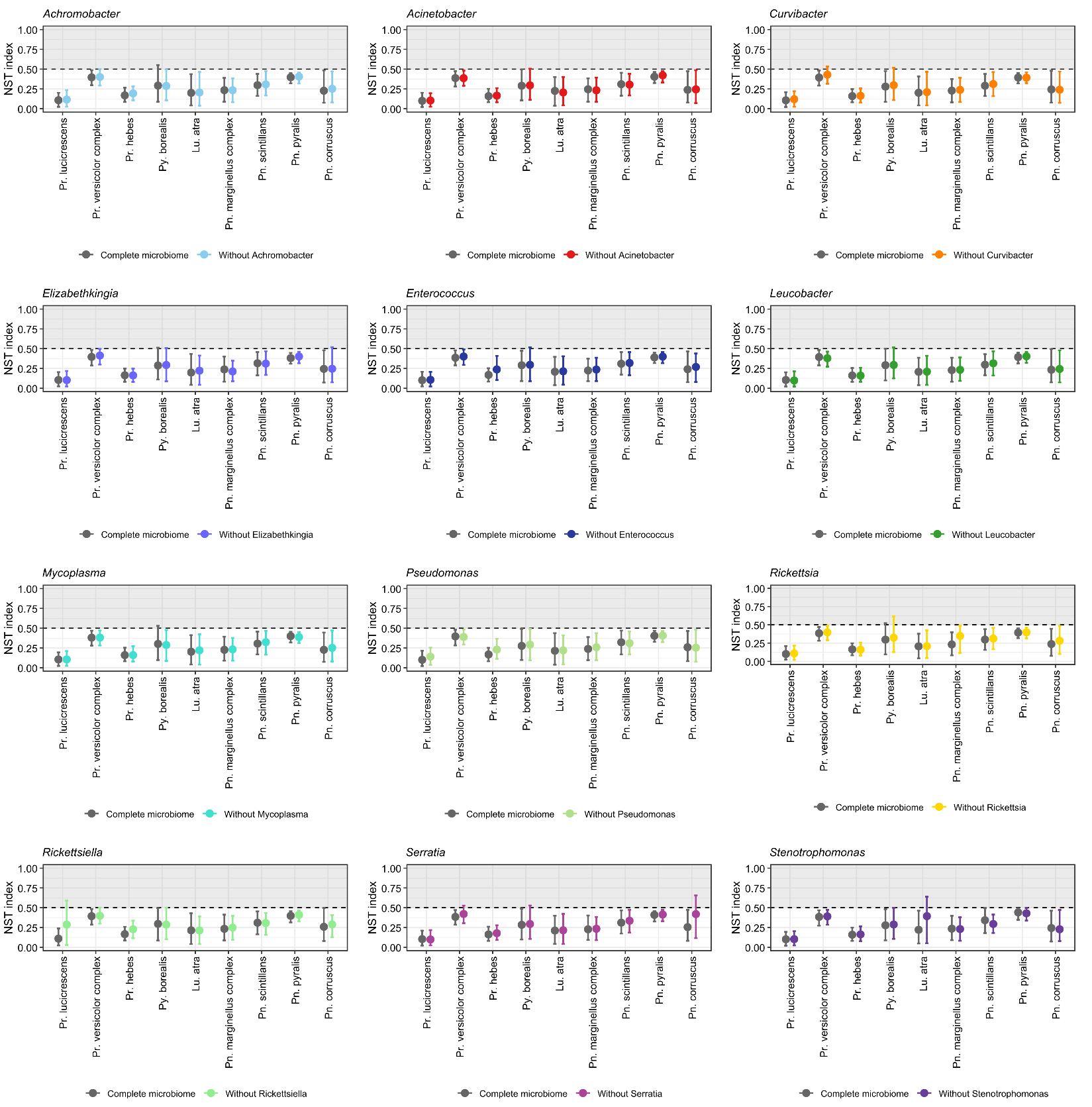
**

**Figure S5: Normalized stochasticity ratio (NST) for 12 prevalent bacterial genera across firefly species**. NST index values higher than 0.5 indicate a higher importance of stochastic forces governing bacterial community assembly, whereas values lower than 0.5 point to deterministic forces as the main drivers of community assembly. For each firefly species or species group the balls correspond to the index value and the error bars are 95% confidence intervals obtained from bootstrapping. Dark grey color is for the complete, untouched microbiome, and colored colors correspond to microbiomes in which specific genera were removed. No significantly different NST values between the complete and modified microbiomes were found for either of these 12 bacterial genera. Two-tailed Wilcoxon signed-rank statistical tests were used to test the significance of these differences. Index values and statistical test results can be found in Table S6.

**
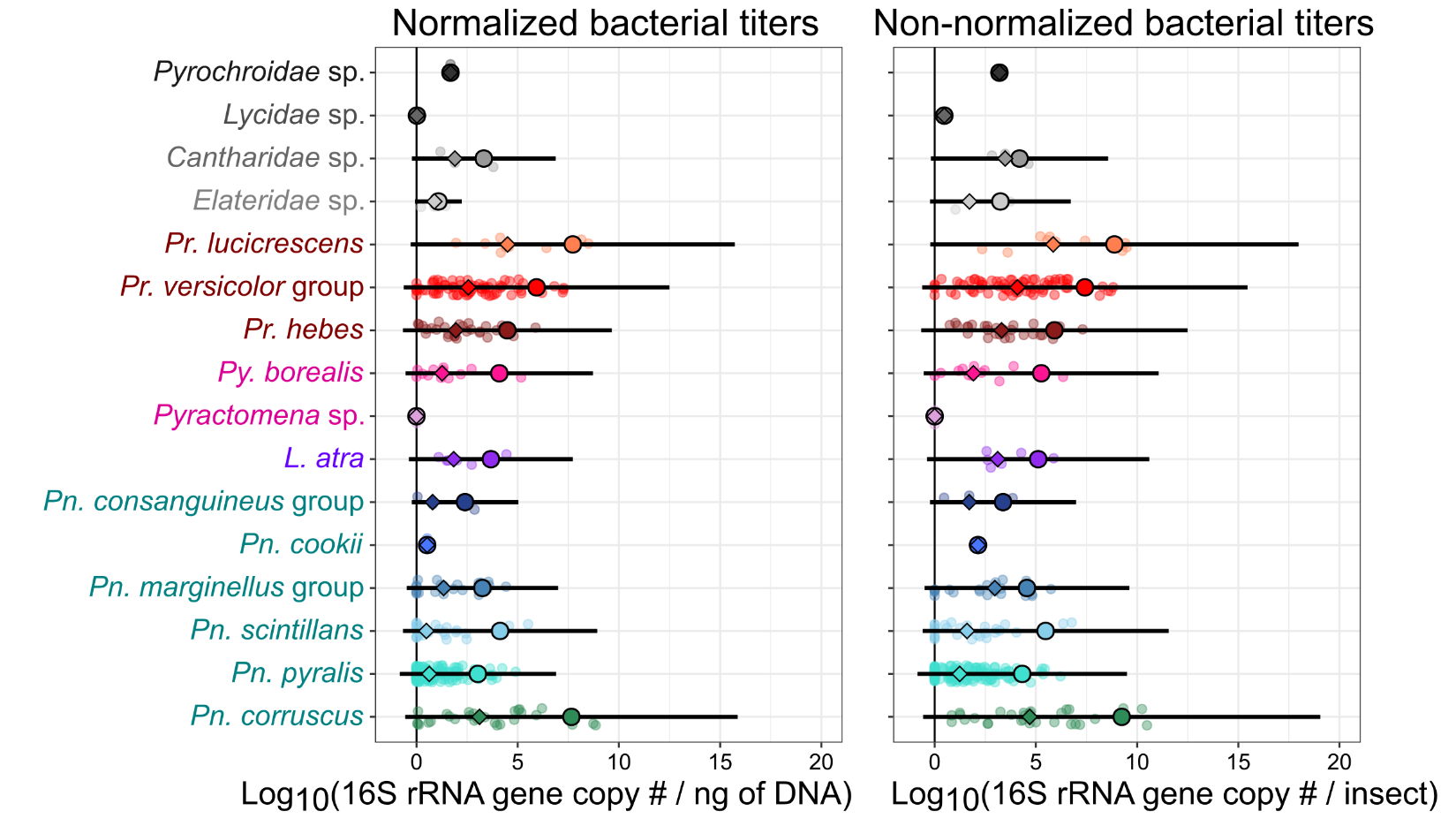
**

**Figure S6: Normalized and non-normalized absolute bacterial abundance across all firefly and related insect samples**. Values are expressed as the log_10_ of the number of 16S rRNA gene copies in a sample, measured through qPCR, minus the number of 16S rRNA gene copy in the DNA extraction blank sample from the same batch, and then, only for normalized, divided by the total DNA concentration in the sample, which was measured via Qubit. Balls, diamonds, and error bars represent the average, median and standard deviation, respectively. Raw qPCR data can be found in Table S3.

**
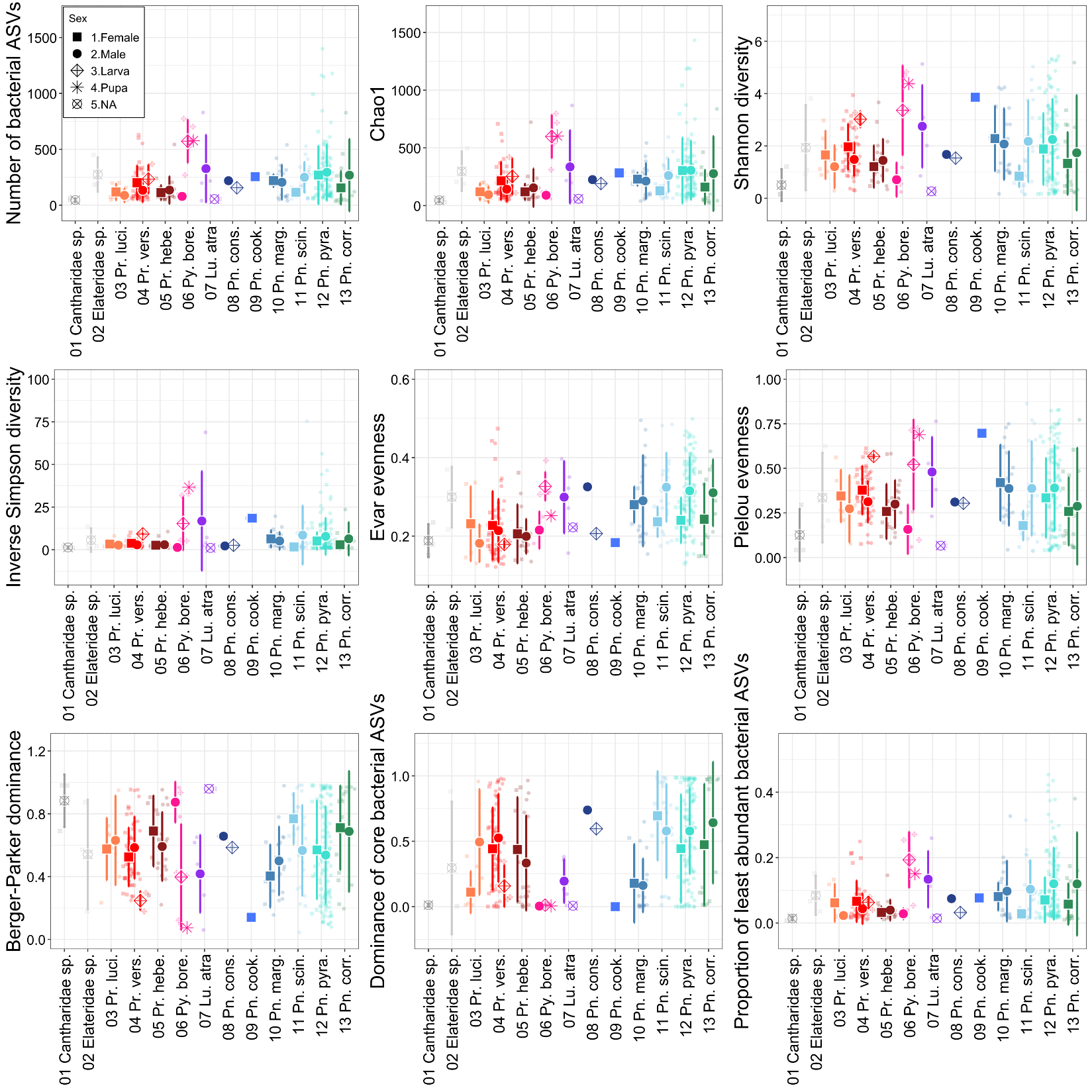
**

**Figure S7: Alpha diversity indices calculated across firefly species, stages, and sexes**. The indices include the ASV richness, the Chao1index, the Shannon diversity index, the inverse Simpson diversity index, the Smith and Wilson’s Evar evenness index, the Pielou evenness index, the Berger-Parker dominance index, the dominance of core bacterial ASVs (those with detection level of 0.1% in over 20% of the samples), and the proportion of the least abundant bacterial ASVs (those below detection level of 0.2% determined separately in each sample) for each firefly species and across sexes and stages. Large size shapes and error bars represent the average and standard deviation, respectively, whereas the dots are the individual index values for each specimen. All alpha diversity metrics can be found in Table S7.

**
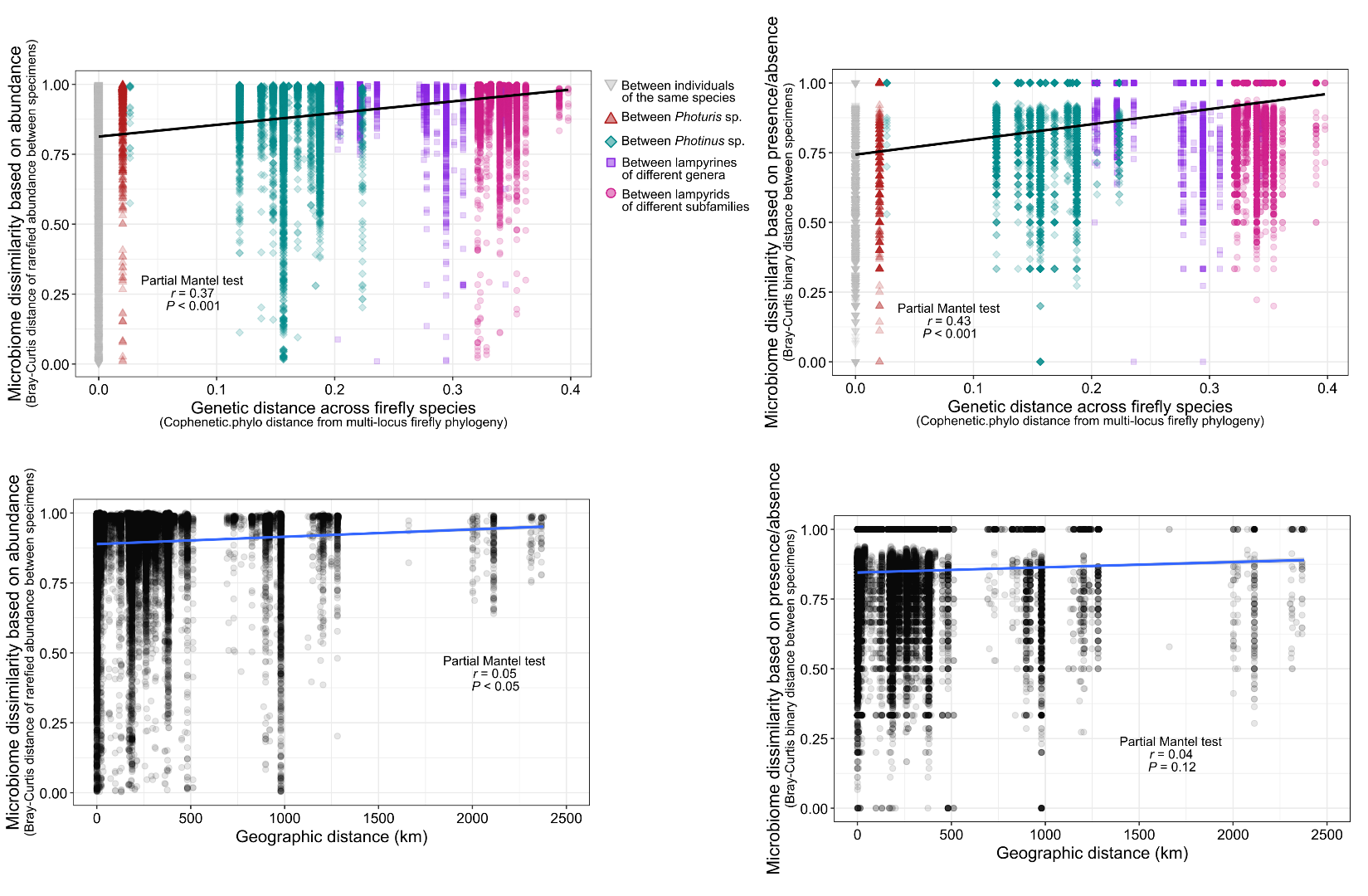
**

**Figure S8: Microbiome dissimilarity increases with genetic and geographic distances**. The graphs at the top show the positive correlation between pairwise phylogenetic distance across firefly species on the x-axis and pairwise microbiome dissimilarity across firefly specimens on the y-axis, calculated from relative rarefied abundance (graph on the left) and presence absence (graph on the right). Colors and shapes of data points represent the level of species comparisons: between individuals of the same species in down-pointing grey triangles, between species from the *Photuris* genus in up-pointing red triangles, between species from the *Photinus* genus in sea green diamonds, between species from the *Lampyrinae* subfamily in purple squares, and between species from different subfamilies in pink circles. The graphs at the bottom show the weak positive correlation between pairwise geographic distance across firefly collection sites on the x-axis (expressed in kilometers) and pairwise microbiome dissimilarity across firefly specimens on the y-axis, calculated from relative rarefied abundance (graph on the left) and presence absence (graph on the right).

**
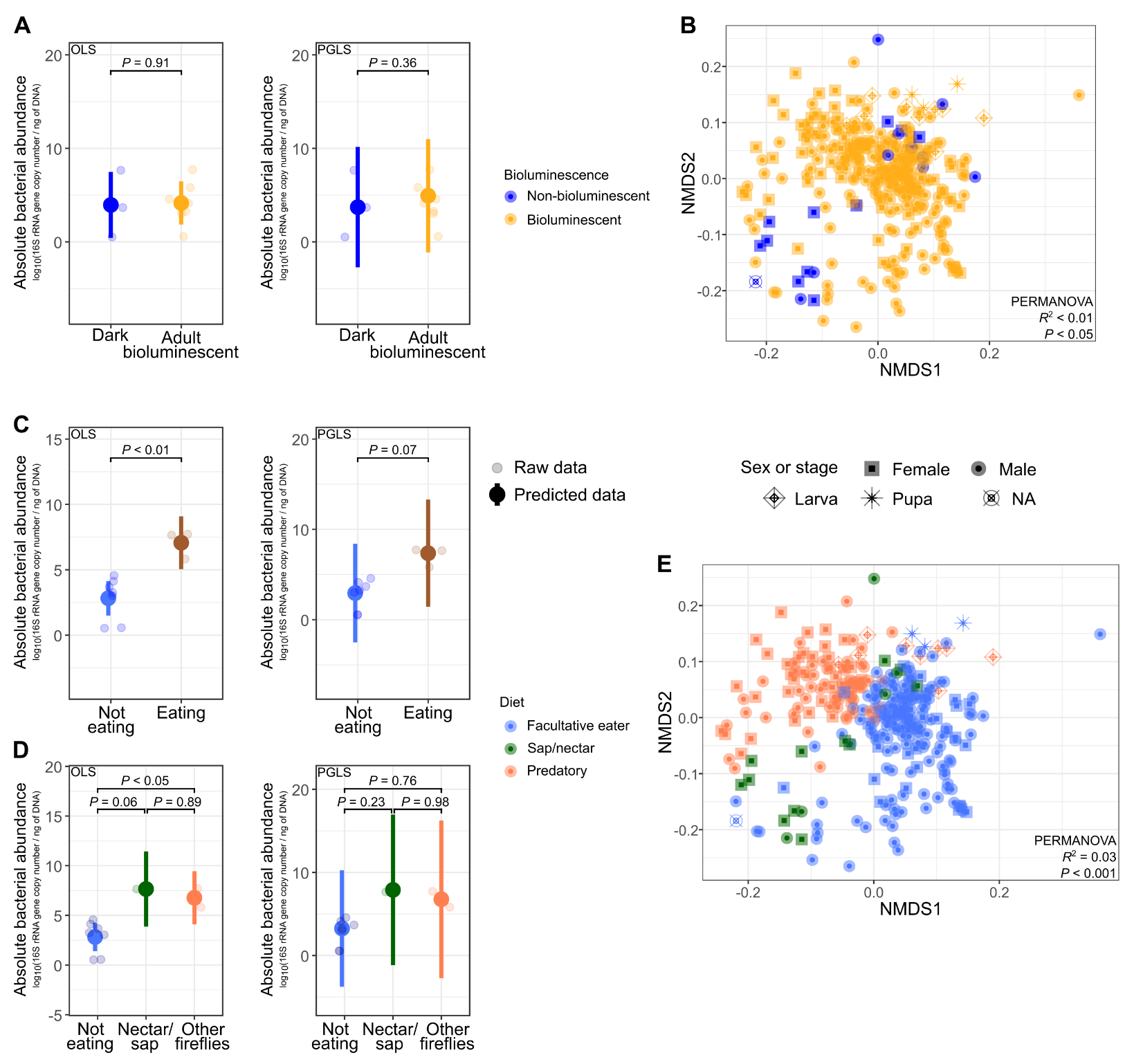
**

**Figure S9: Adult diet, not bioluminescence, influences the microbiome of fireflies**. **A.** Absolute bacterial abundance is not different between bioluminescent and non-bioluminescent species. The Y-axis shows absolute bacterial abundance (expressed as in Figure 1C) and against firefly species grouped as bioluminescent at the adult stage or not on the x-axis. All values were averaged per firefly species. Opaque balls and error bars correspond to predicted averages per group and 95% confidence intervals, respectively, obtained from ordinary least square (OLS) on the left graph and phylogenetic generalized least square (PGLS), which corrects for phylogenetic similarity biases due to the degree of genetic relatedness between firefly species, on the right graph. **B.** Non-metric multidimensional scaling (NMDS) analysis showing separation of the bacterial communities based on composition and relative abundance between bioluminescent and non-bioluminescent fireflies. Four outgroup specimens were removed from these analyses. The NMDS is derived from Bray-Curtis distances calculated on non-rarefied, compositional relative abundance of taxon-aggregated bacteria in firefly specimens. Permutational multivariate analyses of variance (PERMANOVA) were used for statistical testing of group similarities, computed on read depths rarefied at 30,000 reads. **C**. Absolute bacterial abundance (expressed as in Figure 1C) *vs.* firefly species grouped as eaters (*Photinus corruscus*, *Photuris hebes*, *Pr. lucicrescens*, and *Pr. versicolor* group) or non-eaters (all other species). **D**. Absolute bacterial abundance and bacterial richness *vs.* firefly species grouped based on their adult diet: nectar (*Pn. corruscus*), eating other fireflies (*Pr. hebes*, *Pr. lucicrescens*, and *Pr. versicolor* group), or not eating (all other species). **E**. NMDS analysis showing separation of the bacterial communities based on composition and relative abundance across firefly diets. In B and E, the NMDS stress was 0.262.

**
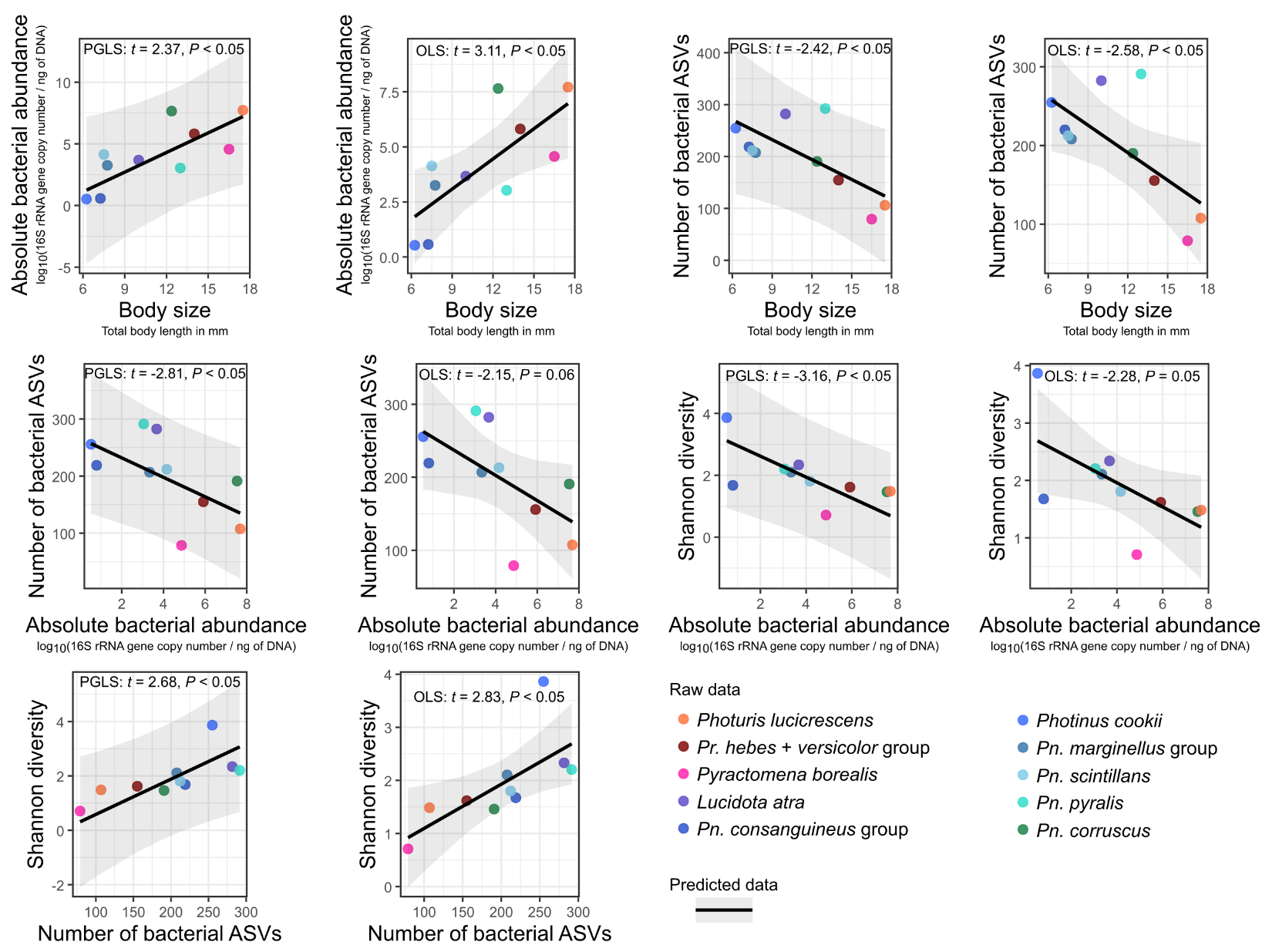
**

**Figure S10: Relationships between firefly body size, absolute bacterial abundance, richness, and diversity**. The first four graphs (from left to right) show the correlation of absolute bacterial abundance (expressed as in Figure 1C) and bacterial richness *vs.* total firefly body length. The other graphs show how absolute bacterial abundance, bacterial richness, and bacterial diversity correlate with one another in fireflies. All values were averaged per firefly species. Predicted correlation lines and 95% confidence interval shades were obtained from ordinary least square (OLS) and phylogenetic generalized least square (PGLS) models, which correct for phylogenetic similarity biases due to the degree of genetic relatedness between firefly species. Dot colors correspond to firefly species.

**
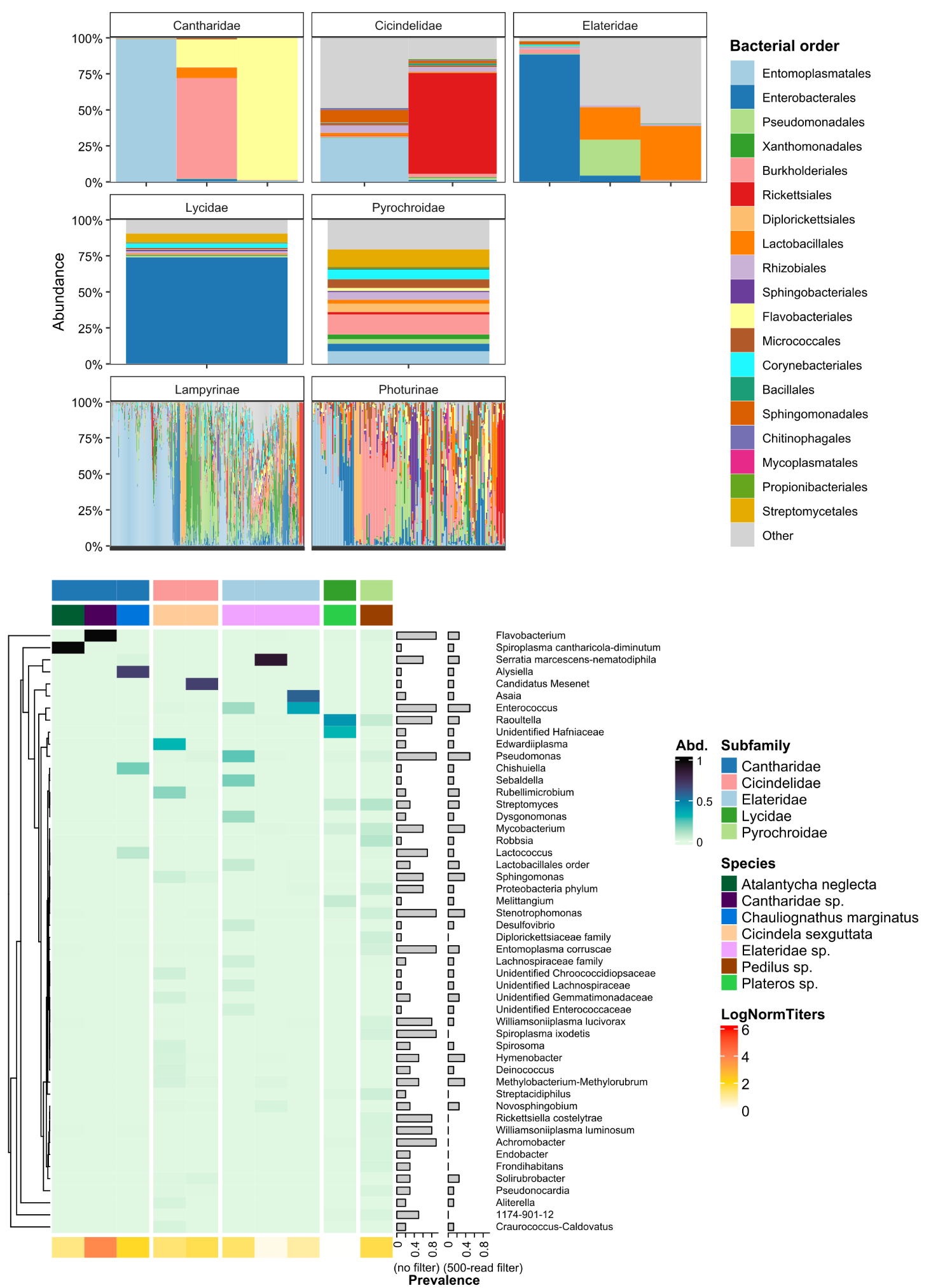
**

**Figure S11: Bacterial composition in non-lampyrid beetles**. The bar graphs at the top represent non-rarefied bacterial composition at the order level in five beetle groups, in addition to two firefly subfamilies. Abundance is the proportion of bacteria per specimen, obtained from compositional transformation of read depth. The composite heatmap at the bottom shows bacterial composition and relative abundance at the “taxon” level, along with total absolute bacterial abundance per specimen (expressed as normalized bacterial titer, see Figure 1C) and prevalence of single bacteria across these beetles, calculated without a filter and with a filter at 100 raw reads (i.e. bacterial taxa with an abundance lower than 100 reads in a specimen were considered absent in the calculation of prevalence). Note that the two tiger beetles (*Cicindelidae*) were already dead at the time of collection.


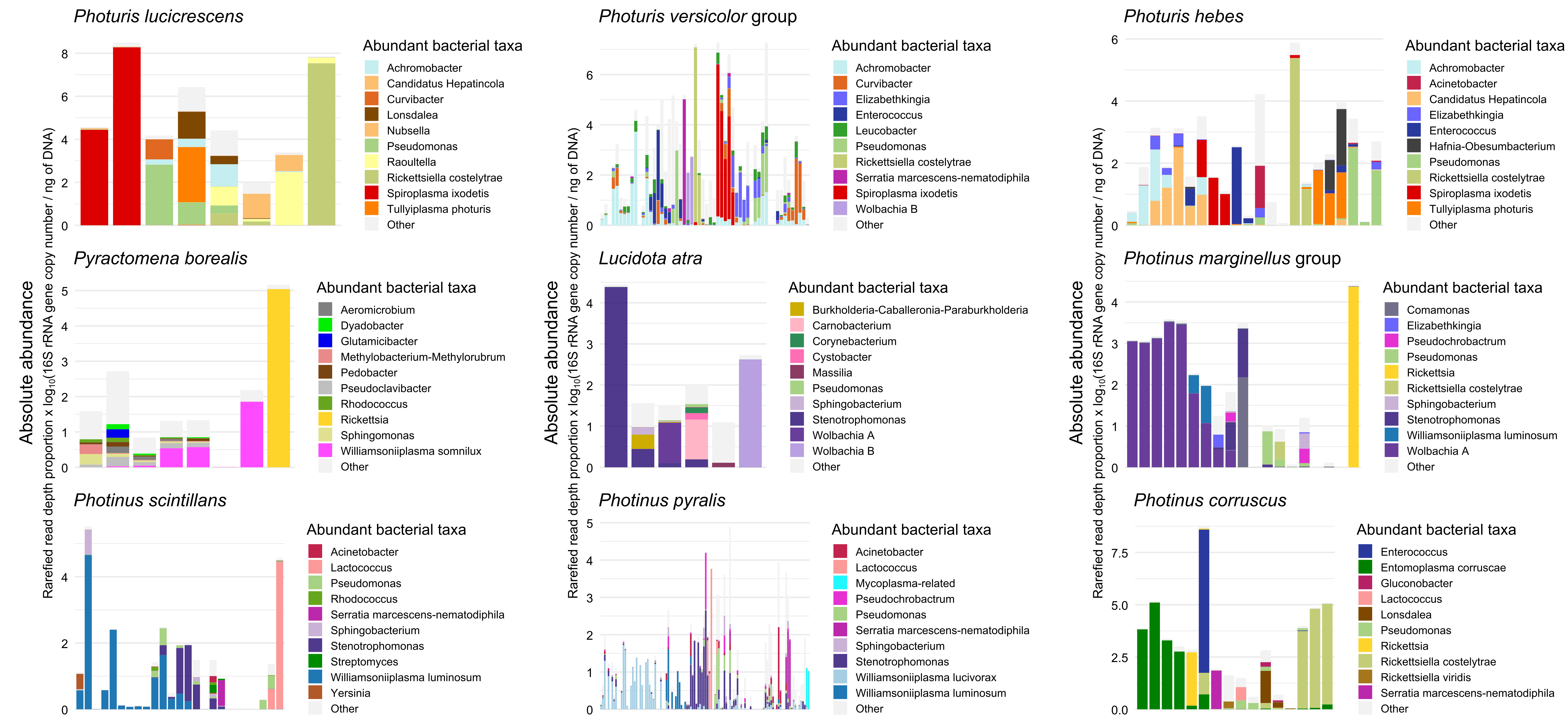


**Figure S12: Absolute abundance of bacterial taxa across firefly species**. In each bargraph the absolute abundance of the top 10 most abundant bacterial taxa per firefly specimen (x-axis) is represented on the y-axis. Absolute abundance was calculated as the relative bacterial taxon abundance (rarefied read depth proportion) was multiplied by total bacterial titer in a specimen (log_10_ of 16S rRNA gene copy number divided by total DNA concentration in specimens).

**
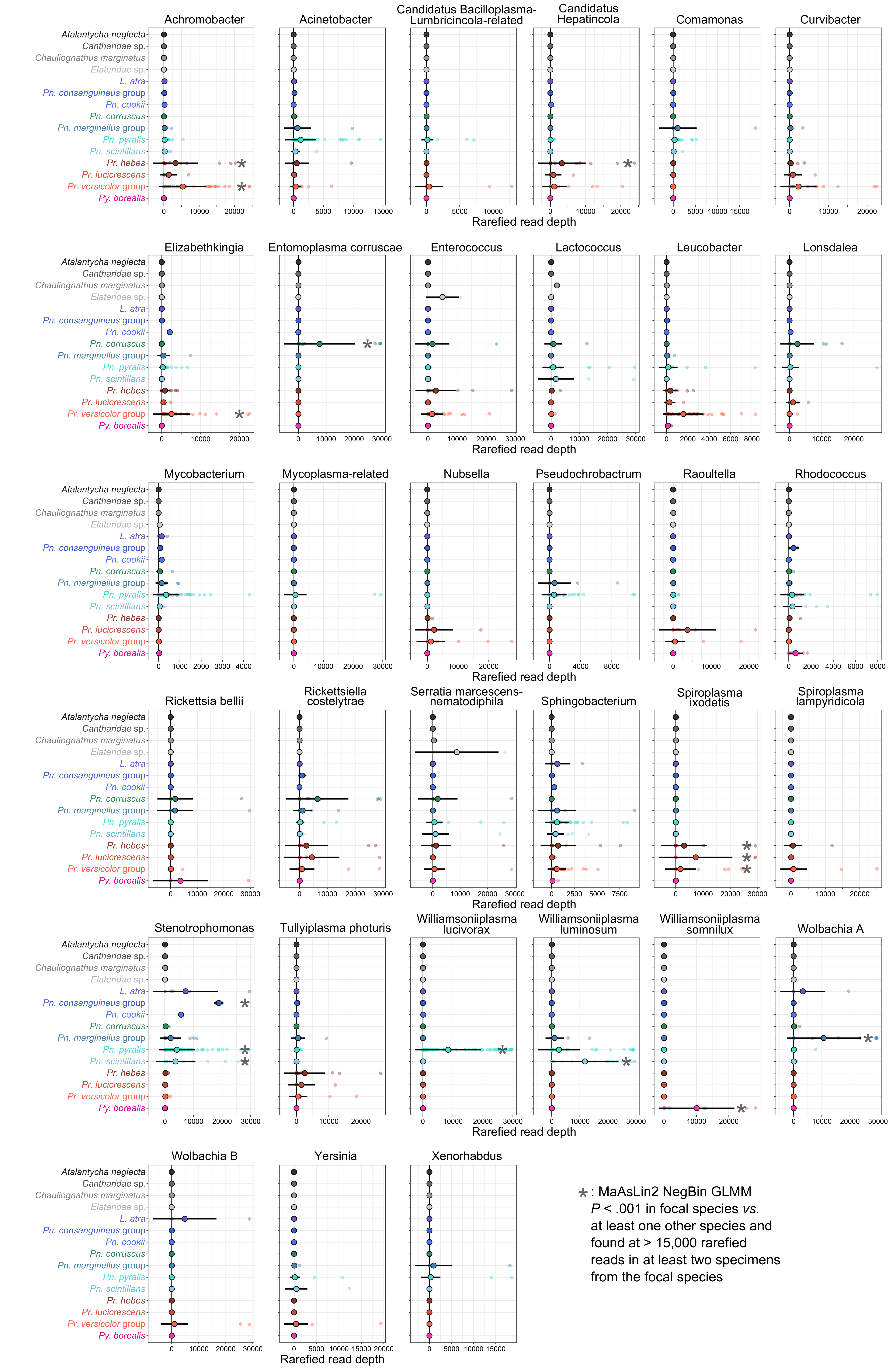
**

**Figure S13: Abundance of 33 bacterial taxa across firefly species and other elateroid beetles**. These bacteria are among the most abundant and prevalent in fireflies from our dataset. For each of them, rarefied read depth (at 30,000 reads) is shown on the x-axis. Firefly species are on the y-axis. Balls and error bars represent the per species average and standard deviation, respectively. The asterisks indicate when a bacterium was considered as a characteristic bacterial taxon in a given firefly species. To account for prevalence, abundance, and other random effects, we classified bacterial taxa as characteristic of a firefly species if (1) *MaAsLin2* multiple generalized linear mixed models indicated that they were significantly more abundant in a given species than in at least another one with a *P* < 0.001, and (2) they were found with an abundance of over 15,000 rarefied read depth in at least two specimens from the focal species. A summary of the *MaAsLin2* results is available in Table S8.

**
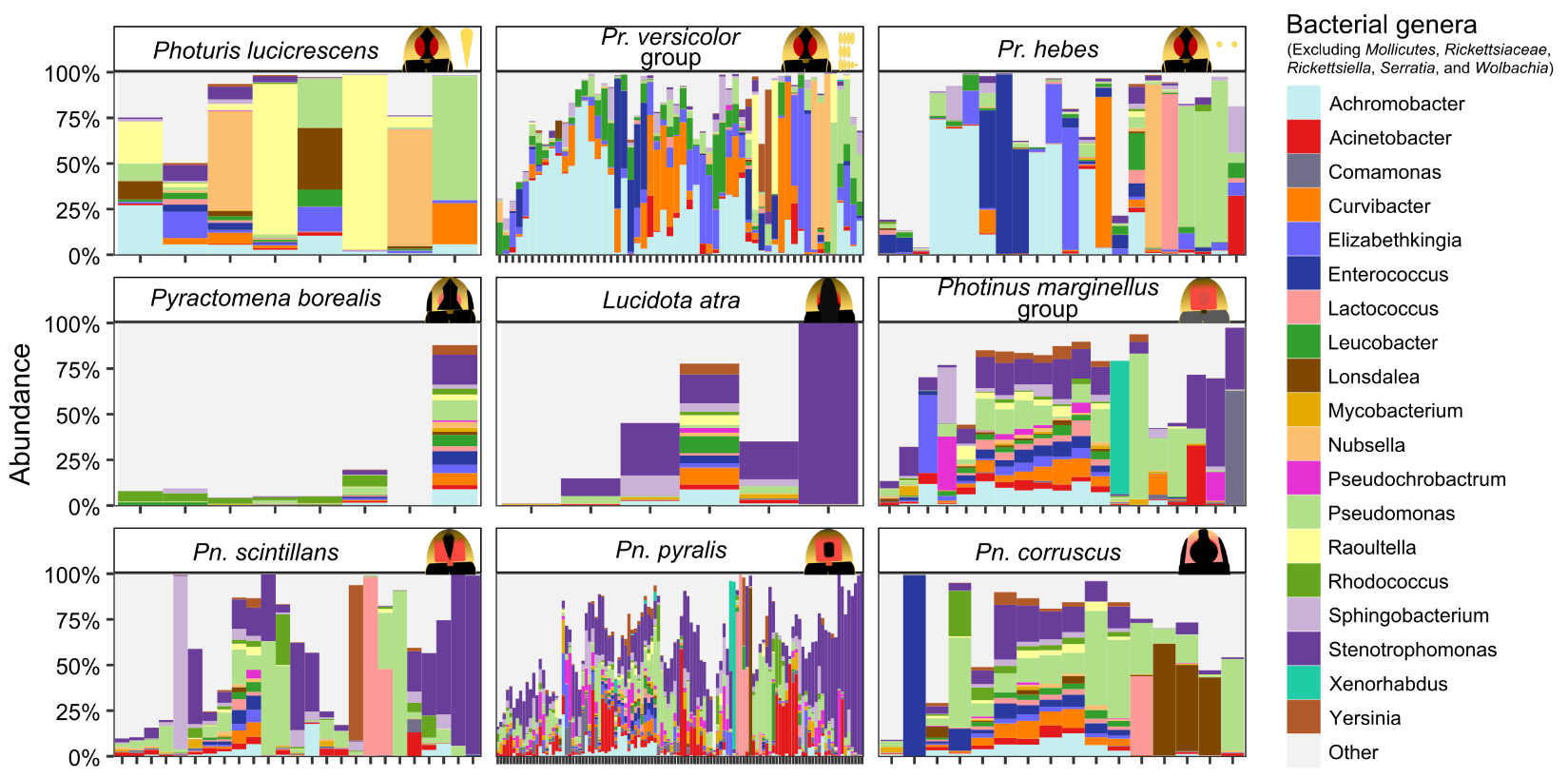
**

**Figure S14: Composition of bacterial taxa across firefly species after excluding mollicutes and putative endosymbionts**. Only firefly species with more than three specimens in our dataset post rarefying are shown. The relative rarefied abundance of the most abundant bacteria is shown as different colors whereas the abundance of all other bacteria is in light grey. Bacteria classified as from the *Candidatus* Hepatincola, *Mollicutes*, *Rickettsia*, *Rickettsiella*, *Serratia*, and *Wolbachia* were excluded from the dataset before generating these graphs. Bacterial colors here approximately match those in Figure 6 and Figure S17.

**
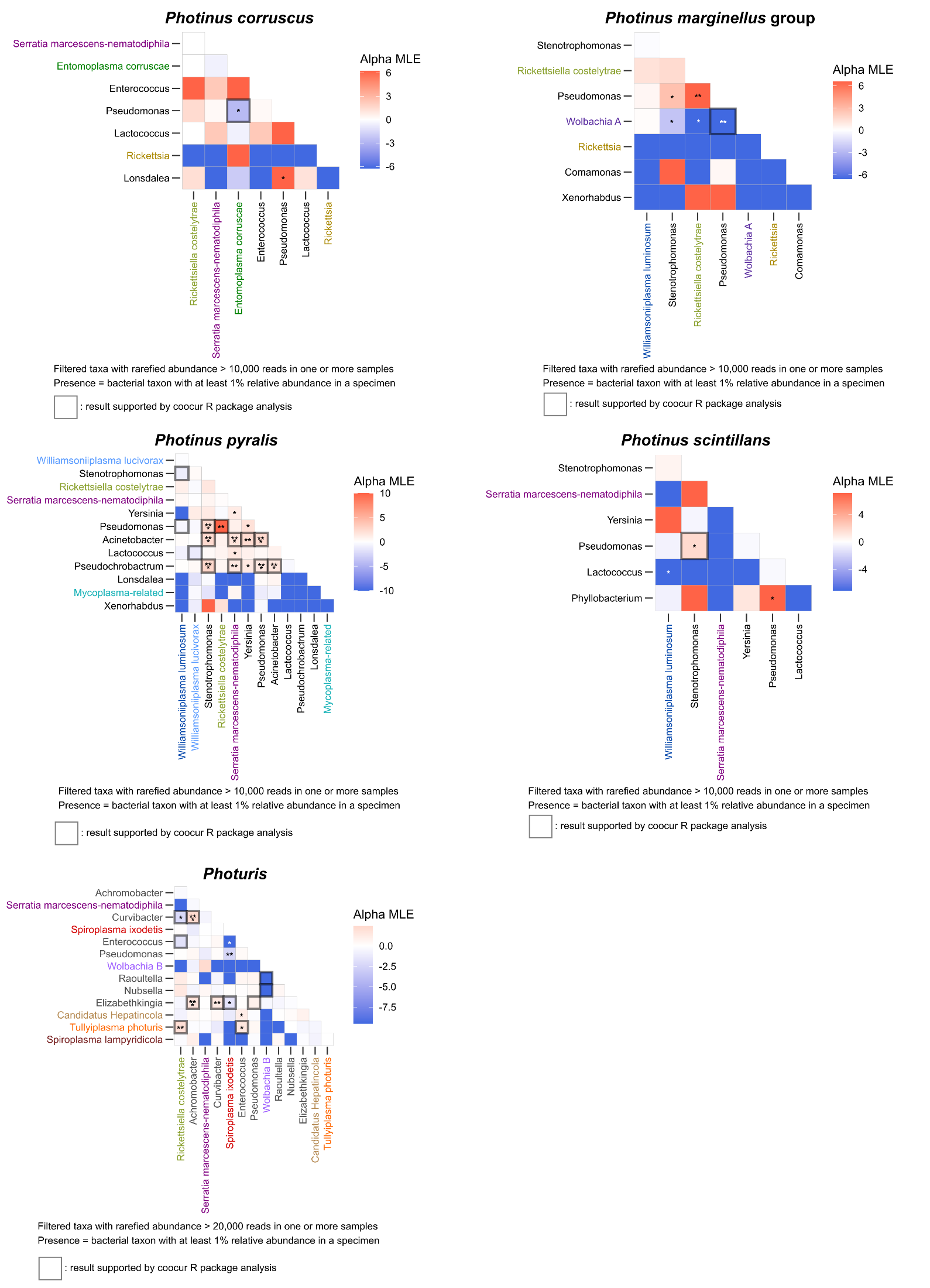
**

**Figure S15: Positive, negative, and random co-occurrence among bacterial taxa associated with fireflies**. The heatmaps were generated with the *CooccurrenceAffinity* R package. Red colors correspond to positive co-occurrence, blue color is for negative co-occurrence, and white for random co-occurrence. The darkest colors, very high or very low alpha MLE values (see Mainali et al., 2022) are for bacterial taxa that almost always or never co-occur, respectively. Dark borders indicate a positive or negative co-occurrence pattern supported by inferences from the *cooccur* R package. Asterisks correspond to different levels of statistical significance in the positive or negative co-occurrence obtained from *CooccurrenceAffinity*, with “.”: *P* < 0.1, “*”: *P* < 0.05, “**”: *P* < 0.01, “***”: *P* < 0.001.

**
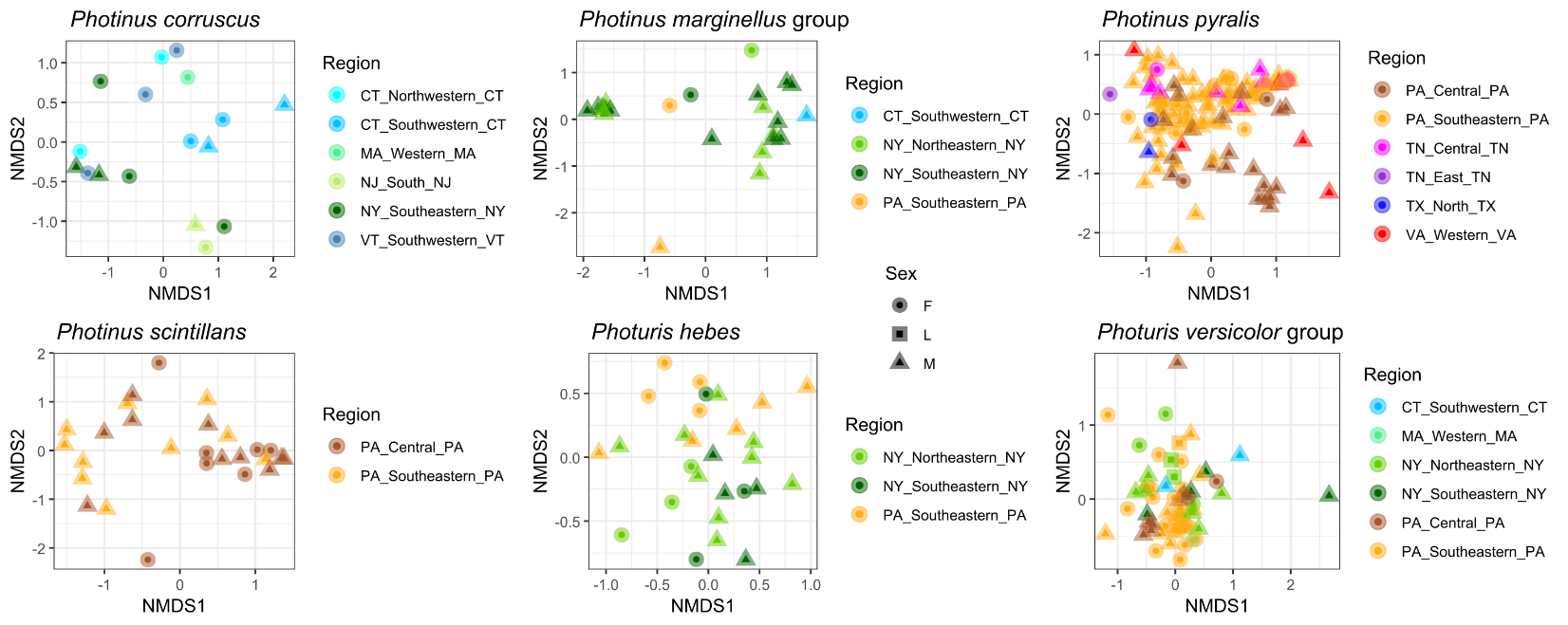
**

**Figure S16: Microbiome divergence across fireflies from different regions**. Non-metric multidimensional scaling (NMDS) analyses showing the separation of the bacterial communities based on composition and relative abundance across regions for four firefly species and two species groups. All plots are derived from Bray-Curtis distances calculated on non-rarefied, compositional relative abundance of taxon-aggregated bacteria in firefly specimens. The NMDS stress values were 0.130 for *Photinus corruscus*, 0.133 for *Pn. marginellus* group, 0.202 for *Pn. pyralis*, 0.148 for *Pn. scintillans*, 0.244 for *Photuris hebes*, and 0.245 for *Pr. versicolor* group. Results from *MaAsLin2* multiple generalized linear mixed models, inferring bacterial differential abundance, can be found in Table S9 and results from PERMANOVAs and pairwise PERMANOVAs comparing regional microbiomes per species can be found in Table S12.

**
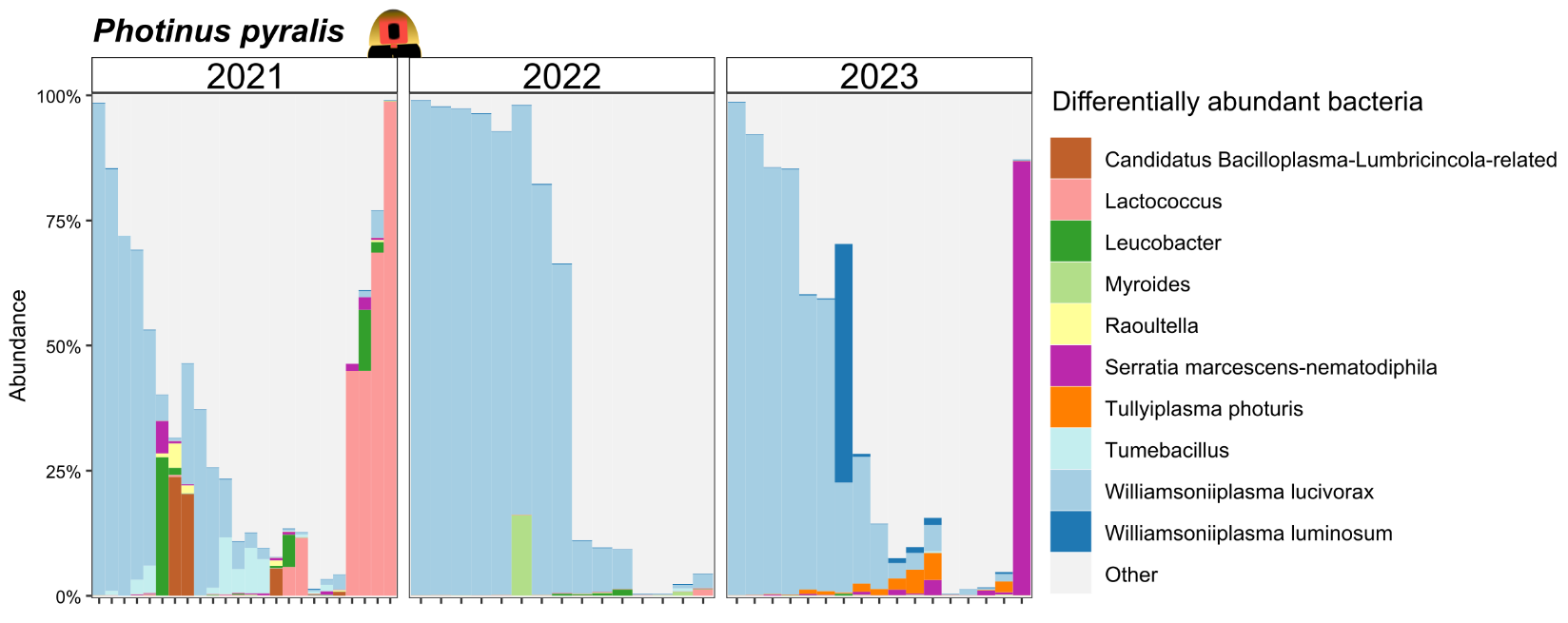
**

**Figure S17: Most differentially abundant bacteria across the years in a firefly species collected in a single location**. *Photinus pyralis* fireflies were collected in Philadelphia Fairmont Park in the month of July 2021, 2022, and 2023. Bacterial colors here approximately match those in Figure 6 and Figure S14.

**
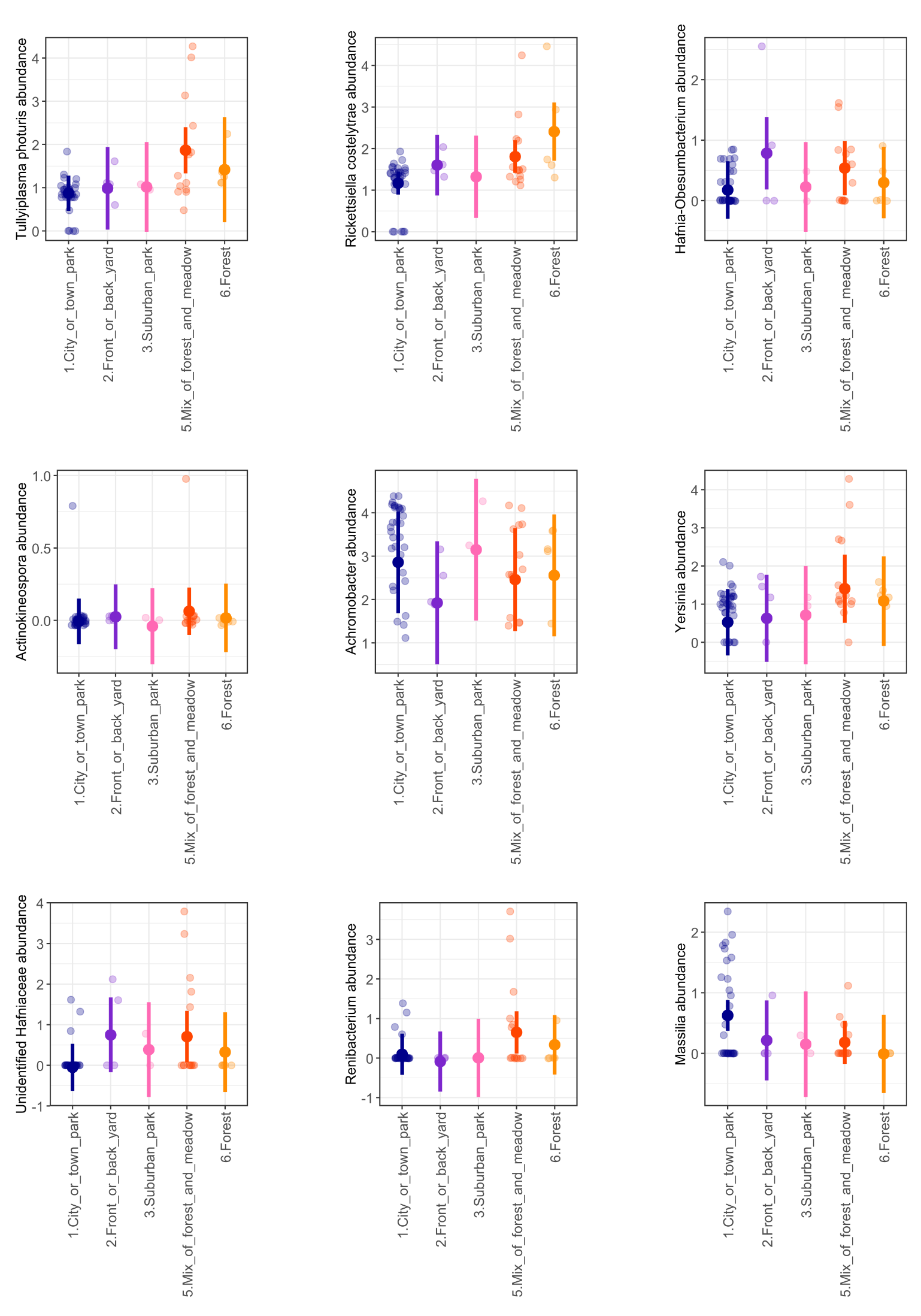
**

**Figure S18: Significantly differentially abundant bacterial taxa across habitat types in unidentified species from the *Photuris versicolor* group**. These bacteria are among those with the most significantly different abundance across habitat in this firefly species group, as assessed through *MaAsLin2* multiple generalized linear mixed models (GLMMs). For each of these bacteria, a univariate GLMM was run to test whether its rarefied abundance differs across these fireflies’ habitats, when controlling for sex type, region, day of year, and year of capture. The most consistent results were observed for *Rickettsiella costelytrae*. Bacterial taxon abundance is expressed as the log_10_ of the bacterial read depth per firefly specimen in our rarefied amplicon sequencing dataset. Results from *MaAsLin2* runs can be found in Table S10.

**
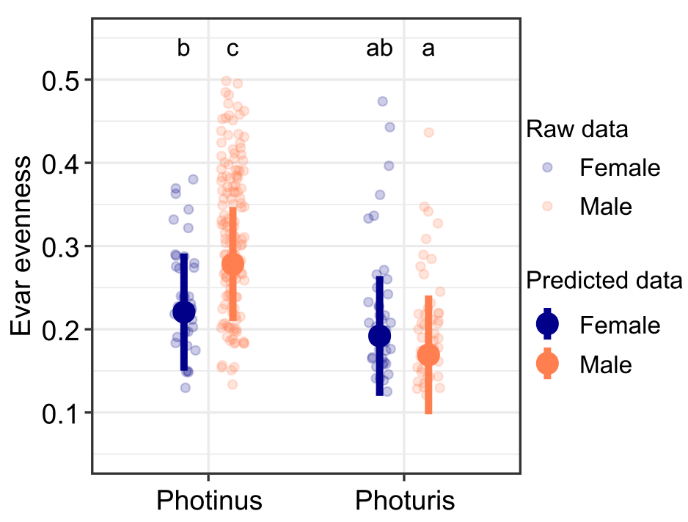
**

**Figure S19: Male *Photinus* microbiomes are more even than females’**. A univariate generalized linear mixed (GLMM) model was used to test whether the index of Evar evenness is different between females and males in *Photinus* and *Photuris* fireflies, when controlling for sampling location, day of year, year of capture, and sequencing type. Significant differences are shown with lowercase English letters. Predicted data with 95% confidence intervals were obtained from the GLMMs. Results from the GLMM can be found in Table S12.

**
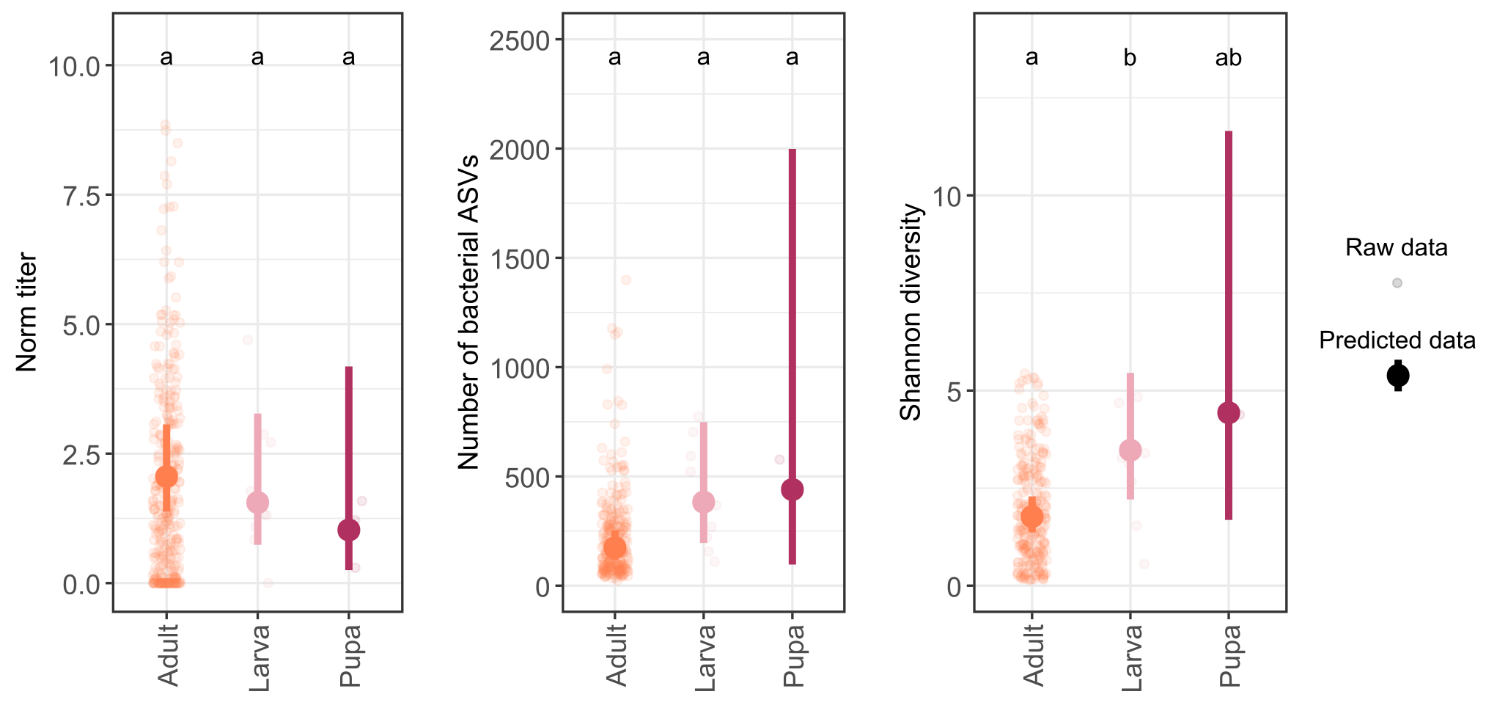
**

**Figure S20: Bacterial absolute abundance and diversity in adults, larvae, and pupae**. This figure is similar to Figure 7F, except that the juvenile category was broken down into larvae and pupae. Univariate generalized linear mixed models (GLMMs) were used to test whether bacterial titer, bacterial richness, and the Shannon diversity index are different between adults, larvae, and pupae, using firefly genus as a covariate and controlling for region, day of year, and year of capture. Bacterial titer is expressed as in Figure 1C. Predicted data with 95% confidence intervals were obtained from the GLMMs. Results from the GLMM can be found in Table S12.

**
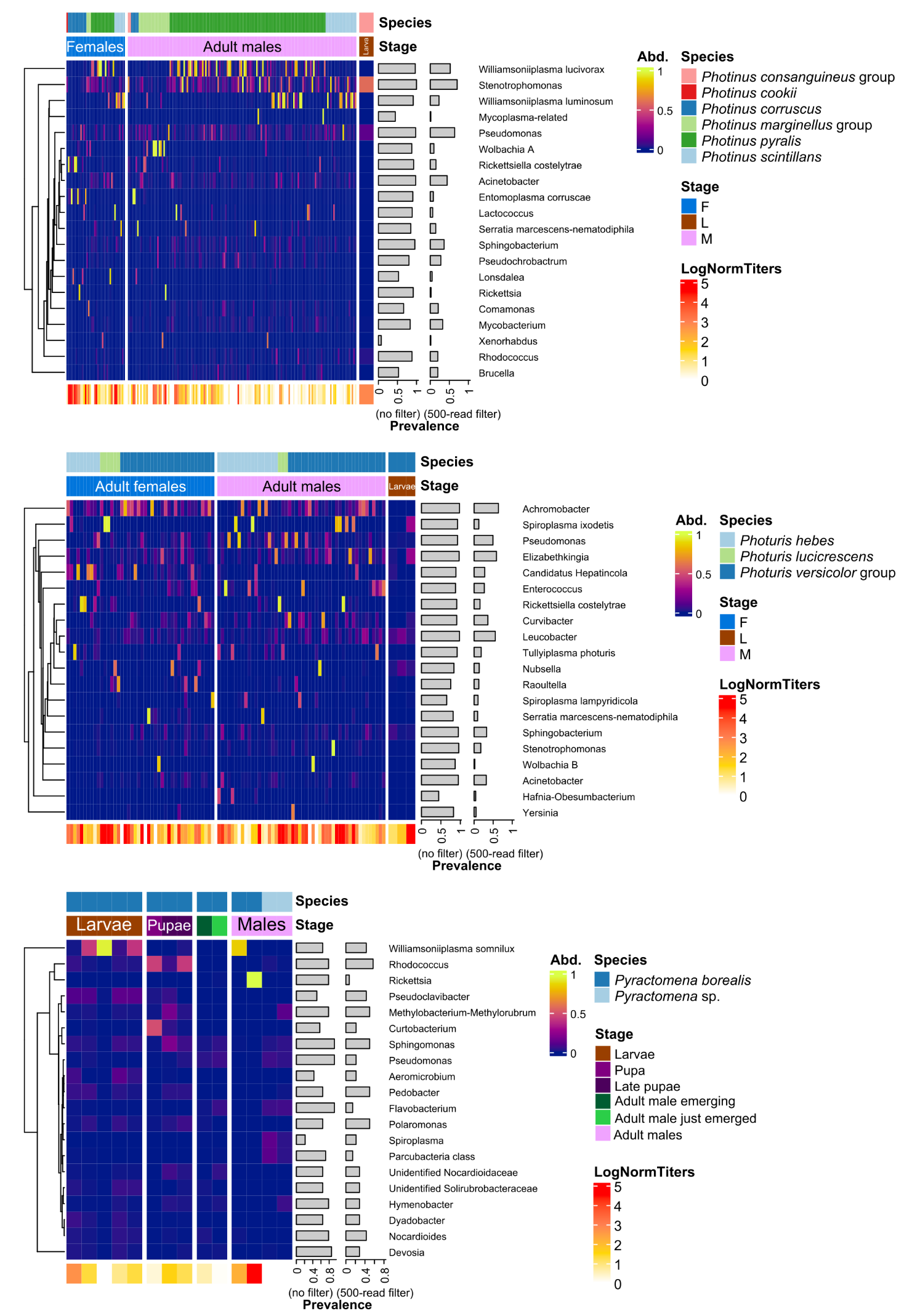
**

**Figure S21: Bacterial composition in larvae and adults of three firefly species**. The composite heatmaps show bacterial composition and relative abundance for the *Photinus*, *Photuris*, and *Pyractomena* genera. Relative abundance values were obtained from compositional transformation of non-rarefied read depth, at the “taxon” level, along with total absolute bacterial abundance per specimen (expressed as normalized bacterial titer, see Figure 1C) and prevalence of single bacteria across these firefly specimens, calculated without a filter and with a filter at 500 raw reads (i.e. bacterial taxa with an abundance lower than 500 reads in a specimen were considered absent in the calculation of prevalence).

**
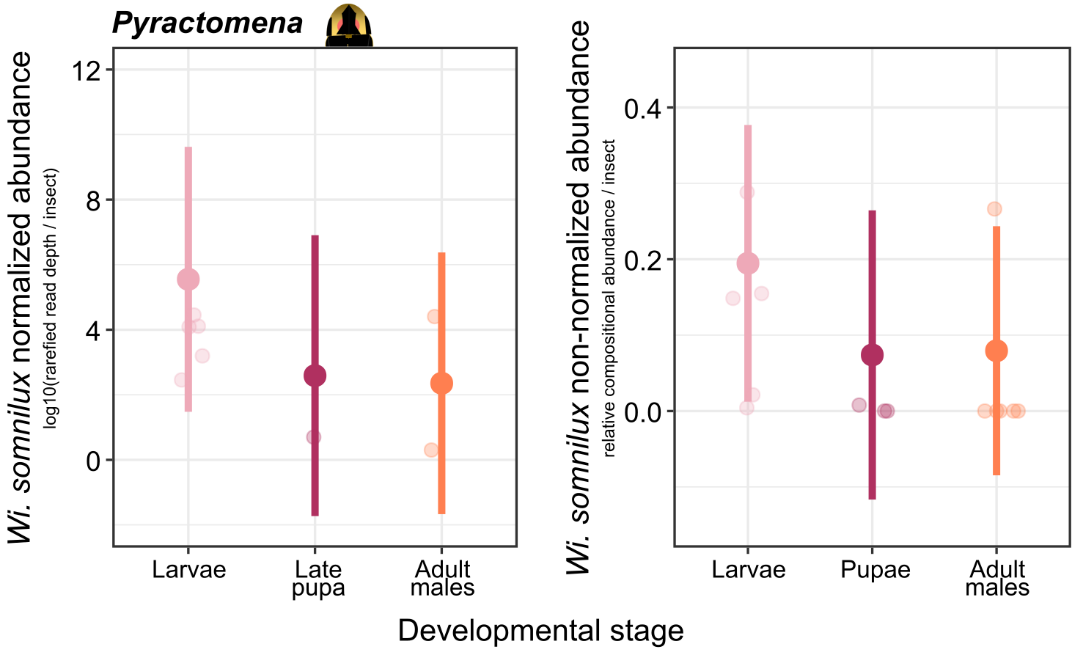
**

**Figure S22: Abundance of *Williamsoniiplasma somnilux* in *Pyractomena* fireflies is higher in larvae than in adults and pupae**. *Wi. somnilux* abundance is expressed as the log_10_ of *Wi. somnilux* read depth per firefly specimen in our rarefied amplicon sequencing dataset in the first panel and as the relative compositional abundance of *Wi. somnilux* in our non-rarefied amplicon sequencing dataset in the second panel (more data points). A univariate generalized linear mixed (GLMM) model was used to test whether *Wi. somnilux* abundance is influenced by developmental stage in *Pyractomena* fireflies, when controlling for species, region, day of year, and year of collection. Predicted mean with 95% confidence intervals were obtained from the GLMMs. Results from the GLMM can be found in Table S12.

**
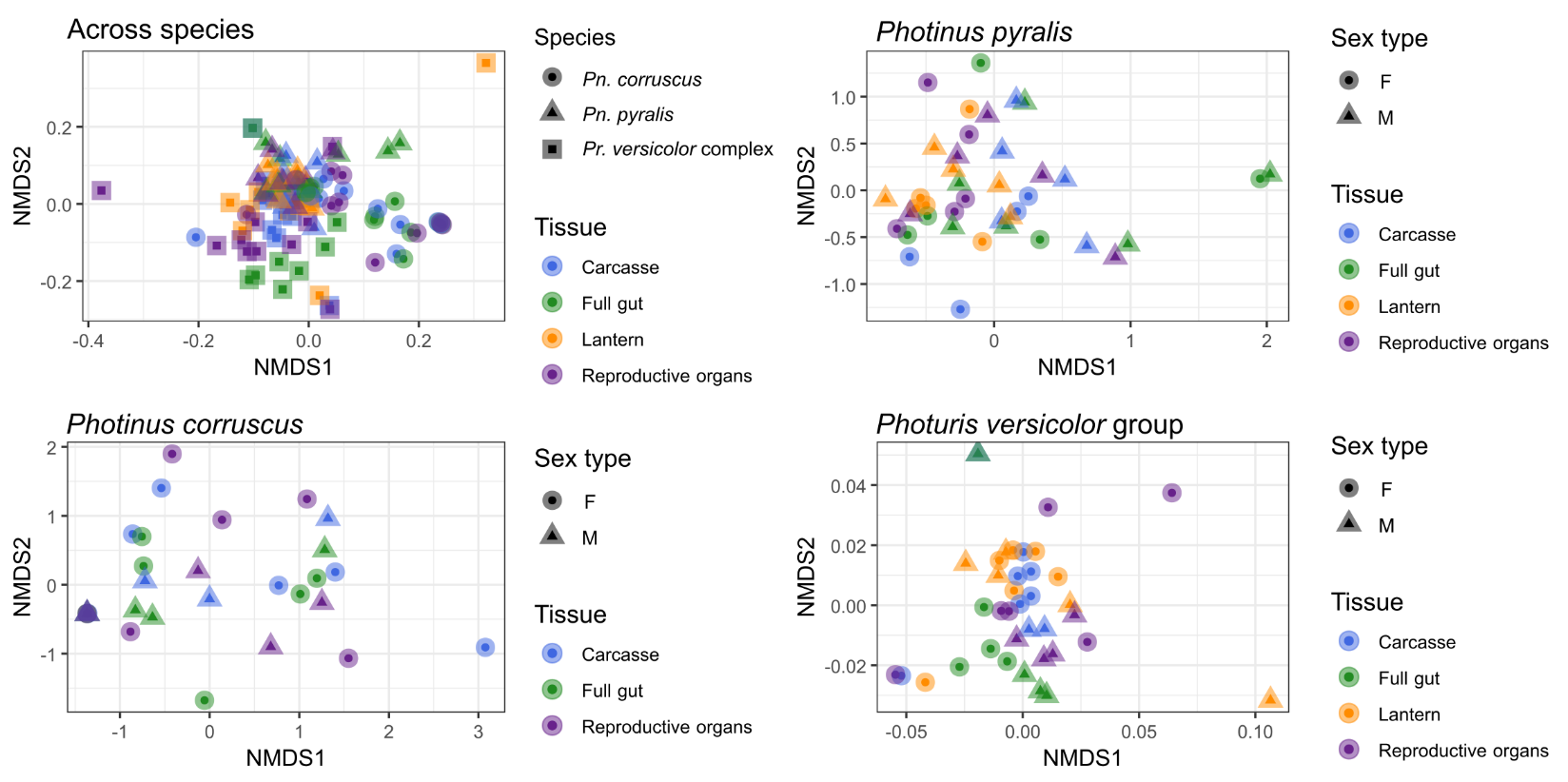
**

**Figure S23:** **Microbiome divergence across firefly tissues**. Non-metric multidimensional scaling (NMDS) analyses showing the separation of the bacterial communities based on composition and relative abundance across tissues for three firefly species and species group. All plots are derived from Bray-Curtis distances calculated on non-rarefied, compositional relative abundance (i.e., “proportional abundance”) of taxon-aggregated bacteria in firefly specimens. One outlier was removed from the first and second graphs. The NMDS stress was 0.218 for across species, 0.179 for *Photinus pyralis*, 0.150 for *Pn. corruscus*, and 0.175 for Photuris *versicolor* group. Results from *MaAsLin2* multiple generalized linear mixed models, inferring bacterial differential abundance, can be found in Table S11 and results from PERMANOVAs and pairwise PERMANOVAs comparing tissue microbiomes per species can be found in Table S12.

**
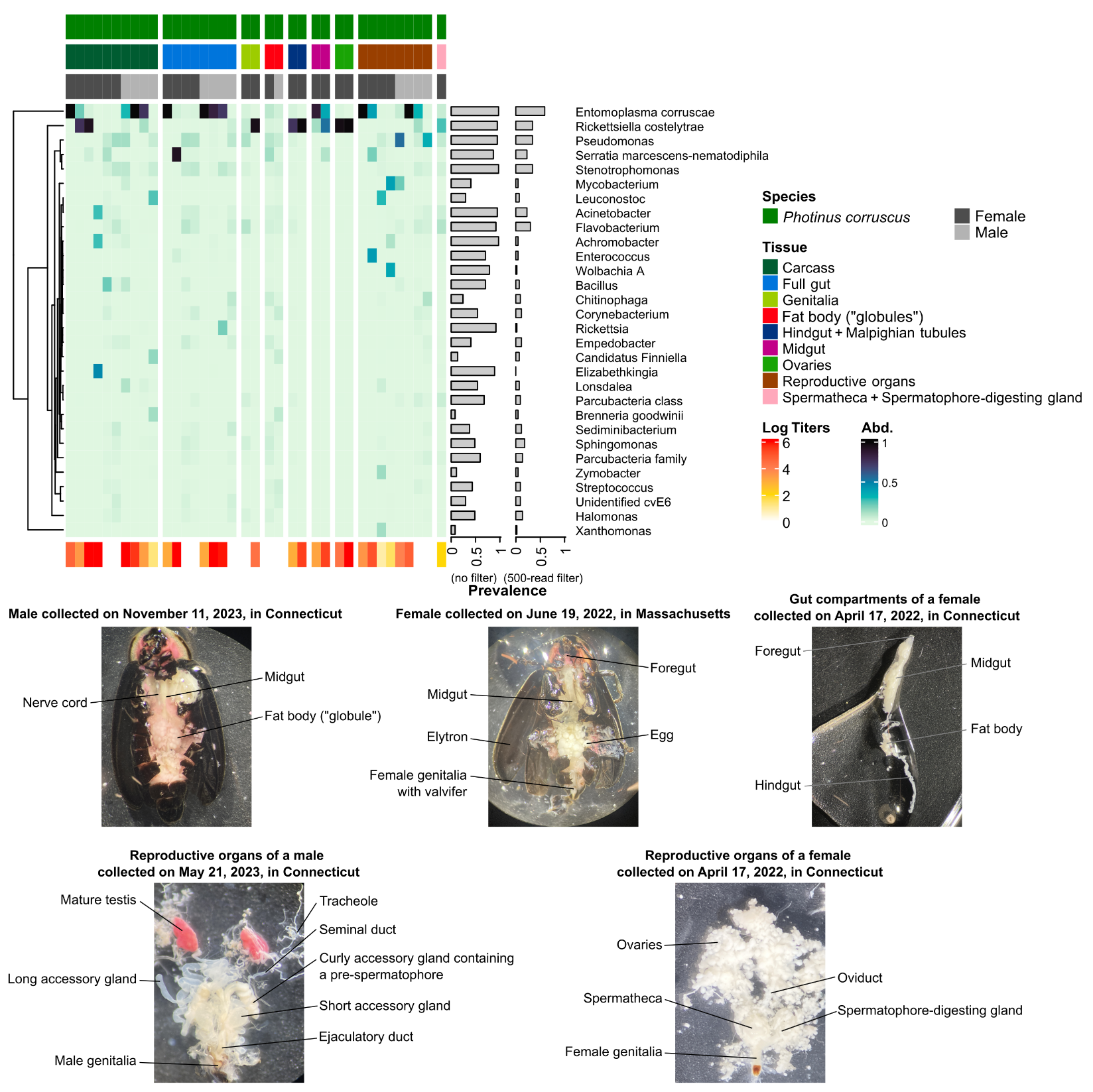
**

**Figure S24:** **Bacterial composition in different tissues of *Photinus corruscus* fireflies**. The composite heatmap at the top shows bacterial composition and relative abundance, obtained from compositional transformation of non-rarefied read depth, at the “taxon” level, along with total absolute bacterial abundance per specimen (expressed as normalized bacterial titer, see Figure 1C) and prevalence of single bacteria across specimen tissues, calculated without a filter and with a filter at 500 raw reads (i.e. bacterial taxa with an abundance lower than 500 reads in a specimen were considered absent in the calculation of prevalence). Photographs of dissections with anatomical annotations are shown at the bottom.

**
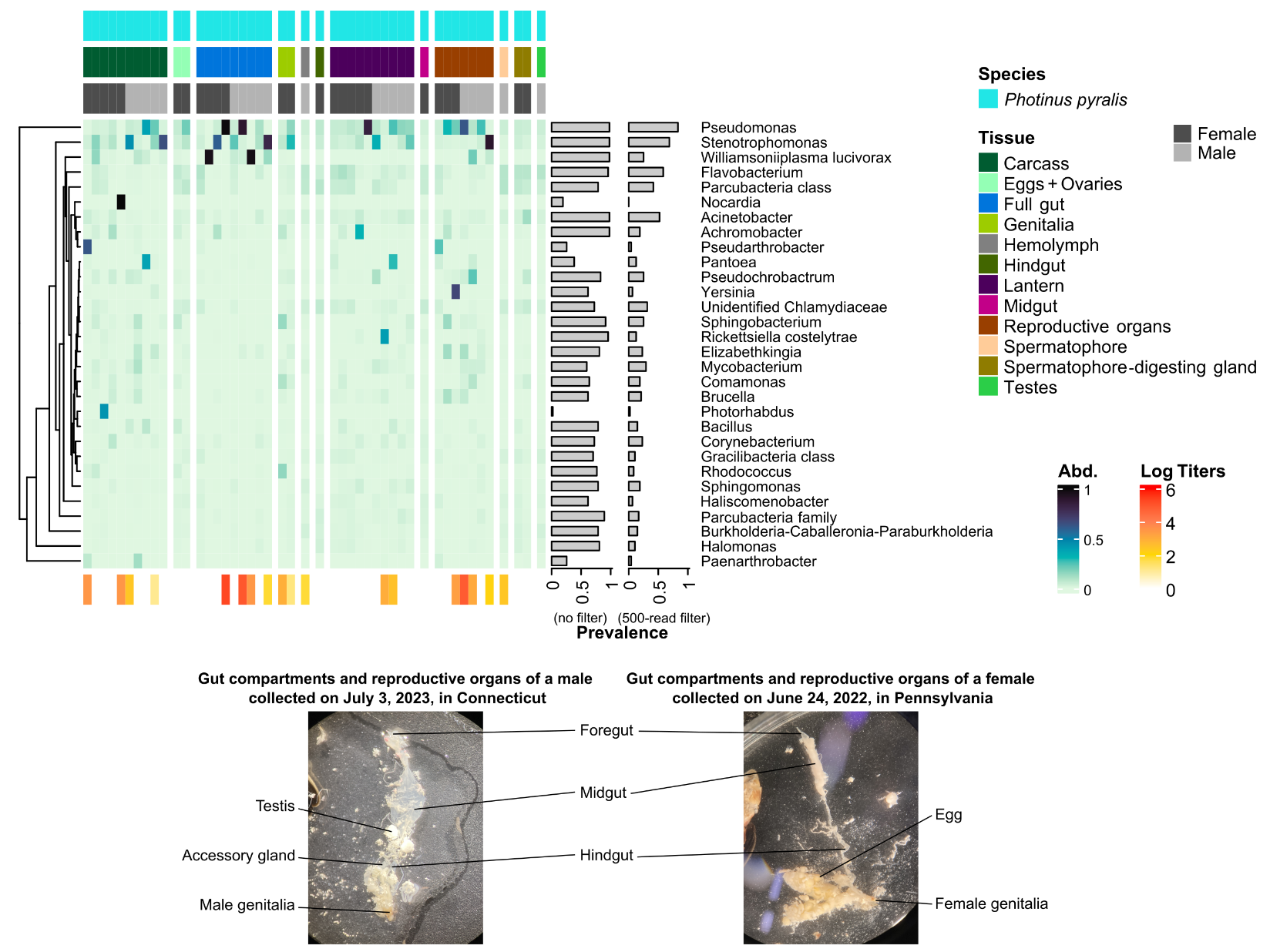
**

**Figure S25:** **Bacterial composition in different tissues of *Photinus pyralis* fireflies**. The composite heatmap at the top shows bacterial composition and relative abundance, obtained from compositional transformation of non-rarefied read depth, at the “taxon” level, along with total absolute bacterial abundance per specimen (expressed as normalized bacterial titer, see Figure 1C) and prevalence of single bacteria across specimen tissues, calculated without a filter and with a filter at 500 raw reads (i.e. bacterial taxa with an abundance lower than 500 reads in a specimen were considered absent in the calculation of prevalence). Photographs of dissected organs with anatomical annotations are shown at the bottom.

**
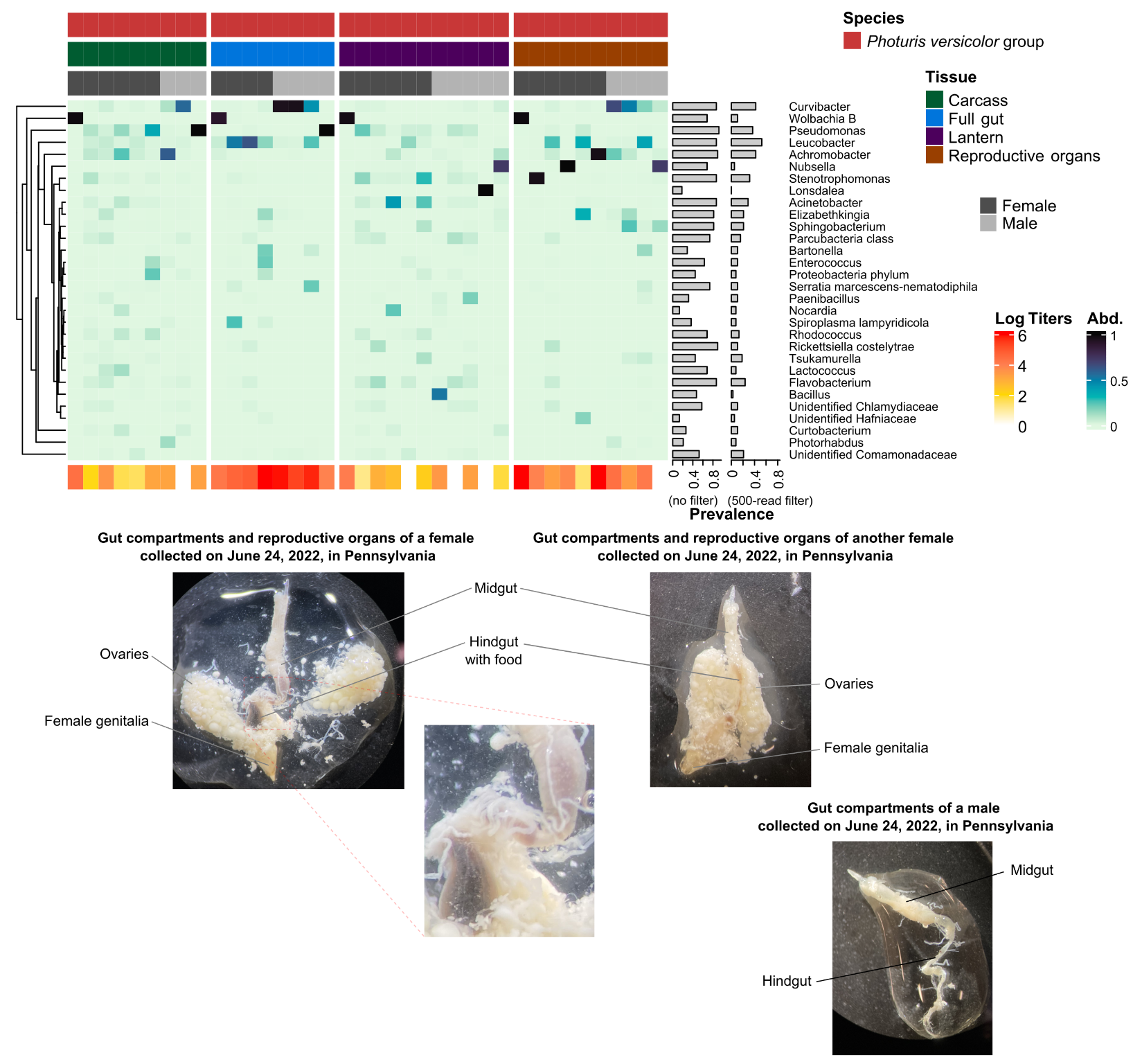
**

**Figure S26:** **Bacterial composition in different tissues of unidentified species from the *Photuris versicolor* group**. The composite heatmap at the top shows bacterial composition and relative abundance, obtained from compositional transformation of non-rarefied read depth, at the “taxon” level, along with total absolute bacterial abundance per specimen (expressed as normalized bacterial titer, see Figure 1C) and prevalence of single bacteria across specimen tissues, calculated without a filter and with a filter at 500 raw reads (i.e. bacterial taxa with an abundance lower than 500 reads in a specimen were considered absent in the calculation of prevalence). Photographs of mouth parts and dissections with anatomical annotations are shown at the bottom.
